# Human Engineered Heart Tissues Reproduce Trigger‐ and Substrate‐Driven Mechanisms of Ventricular Tachycardia

**DOI:** 10.1002/advs.78429

**Published:** 2026-10-26

**Authors:** Matthew Fiedler, Alejandra Vasquez Limeta, Ernesto Reyes Sanchez, Leah Carter, Francisco Altamirano

**Affiliations:** ^1^ Department of Cardiovascular Sciences Houston Methodist Research Institute Houston Methodist Hospital Houston Texas USA; ^2^ Program in Physiology Biophysics & Systems Biology Weill Cornell Medicine New York USA; ^3^ Department of Cardiothoracic Surgery Weill Cornell Medicine New York USA

**Keywords:** arrhythmogenic substrate, cardiac arrhythmia, cardiology, electrophysiology, herg, internal medicine, medicine, optical mapping, reentry, ventricular tachycardia

## Abstract

Human iPSC‑derived engineered heart tissues (EHTs) are used to model cardiac physiology and drug responses, yet whether they reproduce tissue‑scale ventricular arrhythmia mechanisms remains unclear. We combined modified milliPillar EHT fabrication with high‐resolution dual‐channel voltage/Ca^2+^ optical mapping. Baseline electrophysiology showed action potential durations (APD) within the range reported for human ventricles and conduction velocities approximately half of human longitudinal values, with rate‑dependent restitution of APD and Ca^2+^ transient duration, conduction slowing, and physiologic AP‐Ca^2+^ latency. To interrogate a trigger arm of ventricular tachycardia (VT), we modeled acquired long‐QT syndrome (aLQTS; hERG blockade with hypokalemia/hypomagnesemia) in EHTs from two hiPSC lines. aLQTS EHTs developed VT‐like episodes with beat‑to‑beat instability. Early afterdepolarizations propagated heterogeneously, generating triggered activity, tissue acceleration, APD dispersion, wavebreak, and rotor formation. These were short and self‐terminating (median 0.4 s) and clustered at head/neck region curvatures. To test whether a fixed spatial substrate supports prolonged reentry, we developed a reproducible focal cryoinjury model. After injury, all tissues developed VT‐like episodes, and 70% anatomically anchored rotors at the border; three persisted beyond 10 s, a recording‐limited lower bound. Dual voltage/Ca^2+^ mapping at this resolution establishes EHTs as a non‐animal platform for mechanistic study of both trigger‐ and substrate‐driven ventricular arrhythmias.

## Graphical abstract

Engineered heart tissues (EHTs) derived from human iPSCs replicate mechanisms of ventricular tachycardia at the tissue level. Acquired Long‐QT conditions cause early afterdepolarizations, wavebreak, and transient rotors; in contrast, focal cryoinjury forms a stable substrate that supports ongoing reentry. These results position EHTs as a versatile human platform for studying both trigger‐ and substrate‐related ventricular arrhythmias and testing antiarrhythmic therapies.

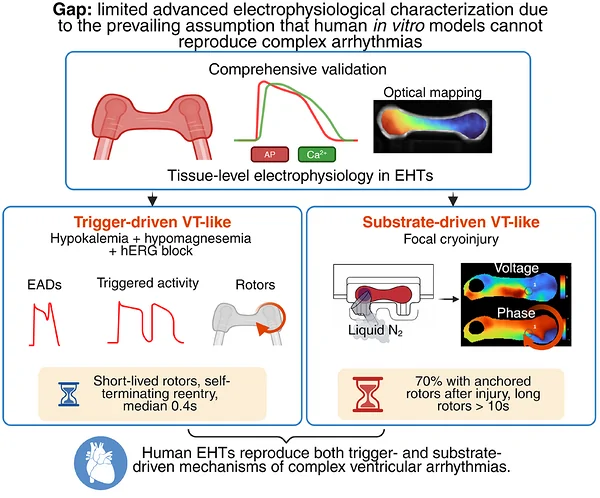

## 1 Introduction

Ventricular tachycardia (VT) and its degeneration into ventricular fibrillation (VF) remain the primary drivers of sudden cardiac death [1]. The clinical presentation is diverse, including genetic disorders such as long QT syndrome (LQTS), and bidirectional interactions with ischemic and non‐ischemic heart failure, where VT leads to heart dysfunction and pathological remodeling that further increases cardiovascular mortality [2, 3]. At the cellular level, VT arises from multiple electrophysiological alterations, including rate‑dependent shortening of the diastolic interval (DI), spatial and temporal heterogeneity of APD, slowed conduction velocity (CV), conduction block, wavebreak, and re‐entrant circuit formation [4]. Early and delayed afterdepolarizations (EADs and DADs) can further aggravate these mechanisms by generating triggered activity, which results in premature beats [5, 6]. High‑resolution optical mapping of voltage or Ca^2+^ signals in animal hearts and human donor‐heart preparations has been indispensable for dissecting these electrophysiological spatiotemporal dynamics and elucidating arrhythmia mechanisms [7]. However, species‐specific baseline electrophysiological differences [8], intrinsic human genetic diversity, and human cardiac remodeling during disease can promote divergent mechanistic paths, motivating human‐based platforms to bridge the translation gap [9, 10, 11].

Human induced pluripotent stem cell‐derived cardiomyocytes (hiPSC‑CMs) provide scalable access to patient‑specific electrophysiology and have revolutionized disease modeling and drug‑cardiotoxicity screening [12, 13]. Yet most hiPSC‑CM electrophysiological studies are confined to single‑cell recordings, monolayers, or organoid assays that rely on measurements which, although informative for cellular mechanisms, lack spatiotemporal resolution [12]. Moreover, these reductionistic platforms lack the structural anisotropy, mechanical load, and multicellular connectivity required to initiate and sustain the tissue‑scale phenomena that underlie sustained VT/VF.

Engineered heart tissues (EHTs) that embed hiPSC‑CMs and fibroblasts in a three‑dimensional format represent an intermediate step between reductionist models and intact hearts. By recapitulating mechanical loading and multicellular composition, EHTs display more physiological excitation‐contraction coupling (ECC) and tissue properties [14, 15, 16]. Nevertheless, high spatiotemporal resolution electrophysiological interrogation of human EHTs remains limited for two principal reasons: (i) the lack of a reproducible, high‑throughput fabrication pipeline that yields tissue constructs compatible with optical mapping studies; and (ii) the difficulty of establishing stable imaging conditions including continuous perfusion, precise temperature control, minimized motion artifacts, and synchronized electrical stimulation required for quantitative voltage and Ca^2+^ mapping at millisecond temporal resolution. A knowledge gap remains regarding whether hiPSC‑CM‑based EHTs can faithfully reproduce the substrates, initiators, and drivers of human arrhythmias that lead to VT/VF, or whether they are limited to artifactual and/or simplified versions of in vitro rhythmic disturbances.

To address this gap, we established a robust workflow for high‐resolution, simultaneous optical mapping of voltage and Ca^2+^ in EHTs. By modifying the open‐source milliPillar EHT platform developed by the Vunjak‐Novakovic Lab [17], we facilitated mold generation for polydimethylsiloxane (PDMS) casting and improved plating conditions for dual‐voltage and Ca^2+^ optical mapping. We developed a compatible 3D‐printed imaging stage that provides perfusion, temperature control, and electrical stimulation while minimizing motion artifacts in buoyant PDMS‐based EHT constructs, using a commercially available optical mapping recording system. Using this system, we first characterized baseline electrophysiological responses in EHTs at 22 µm spatial and 1 ms temporal resolution and observed results similar to those from previously published adult hearts. We then challenged the tissues with a pro‑arrhythmic milieu: low extracellular K^+^/Mg^2+^ concentration (low K^+^/Mg^2+^) combined with selective hERG blockade, which reproduces acquired LQTS (aLQTS) complications leading to VT and the highly lethal arrhythmia Torsades de Pointes (TdP) [18, 19]. Analysis of beat‑to‑beat APD, DI, and upstroke velocity (dV/dt) together with phase‑singularity (PS) and wavelet tracking reveals EHTs repolarization recovery failure, localized conduction slowing, functional block, and wavebreak, culminating in short‑lived re‑entrant rotors. These alterations were driven by localized and transient APD dispersion gradients that created long‐short APD regions, a known substrate for the initiation of human arrhythmias.

These aLQTS observations illustrate the trigger arm of VT. To address the substrate arm independently—a fixed, non‐conducting structural obstacle of the kind created by regional cardiomyocyte loss – we introduced focal cryoinjury in a subset of EHTs and mapped the resulting arrhythmic and rotor phenotype. Cryoinjury places a discrete, reproducible region of cardiomyocyte loss in otherwise healthy EHTs, allowing us to ask whether a structural discontinuity alone can anchor reentry in EHTs on an acute timescale. This is the classical use of the ex vivo perfused‐heart cryoinjury preparation, in which a fixed non‐conducting obstacle is created within continuously perfused myocardium, isolating anatomically anchored reentry from ischemia, inflammation, and fibrosis [20, 21, 22]. Together, the trigger and substrate arms demonstrate that hiPSC‐derived EHTs reproduce tissue‐scale ventricular arrhythmia mechanisms previously accessible only in intact hearts, while also highlighting the size‐related limits on sustained VF modeling in millimeter‐scale constructs.

## 2 Results

### 2.1 Implementation of Modified Individual milliPillar Modules

To increase throughput and compatibility with optical mapping, we made minor modifications to the milliPillar platform [17]. Comprehensive details of mold modifications, print orientation, post‐processing, and the STL file are in the Supplemental data (Tables S1 and S2, STL File 1, and Figure 1). These changes allow multiple molds to be printed on a low‐cost consumer resin printer (Figure 1A). After PDMS casting, individual milliPillar modules were trimmed and attached to multi‐well plates, facilitating plating, media exchange, and detachable intact EHTs for optical mapping (Figures 1B and S2; Video S1). This modularity enabled rapid iteration of handling and imaging configurations (e.g., longitudinal electrical pacing with C‐Pace, IonOptix followed by terminal optical mapping) without affecting tissue structure or function.

**FIGURE 1:**
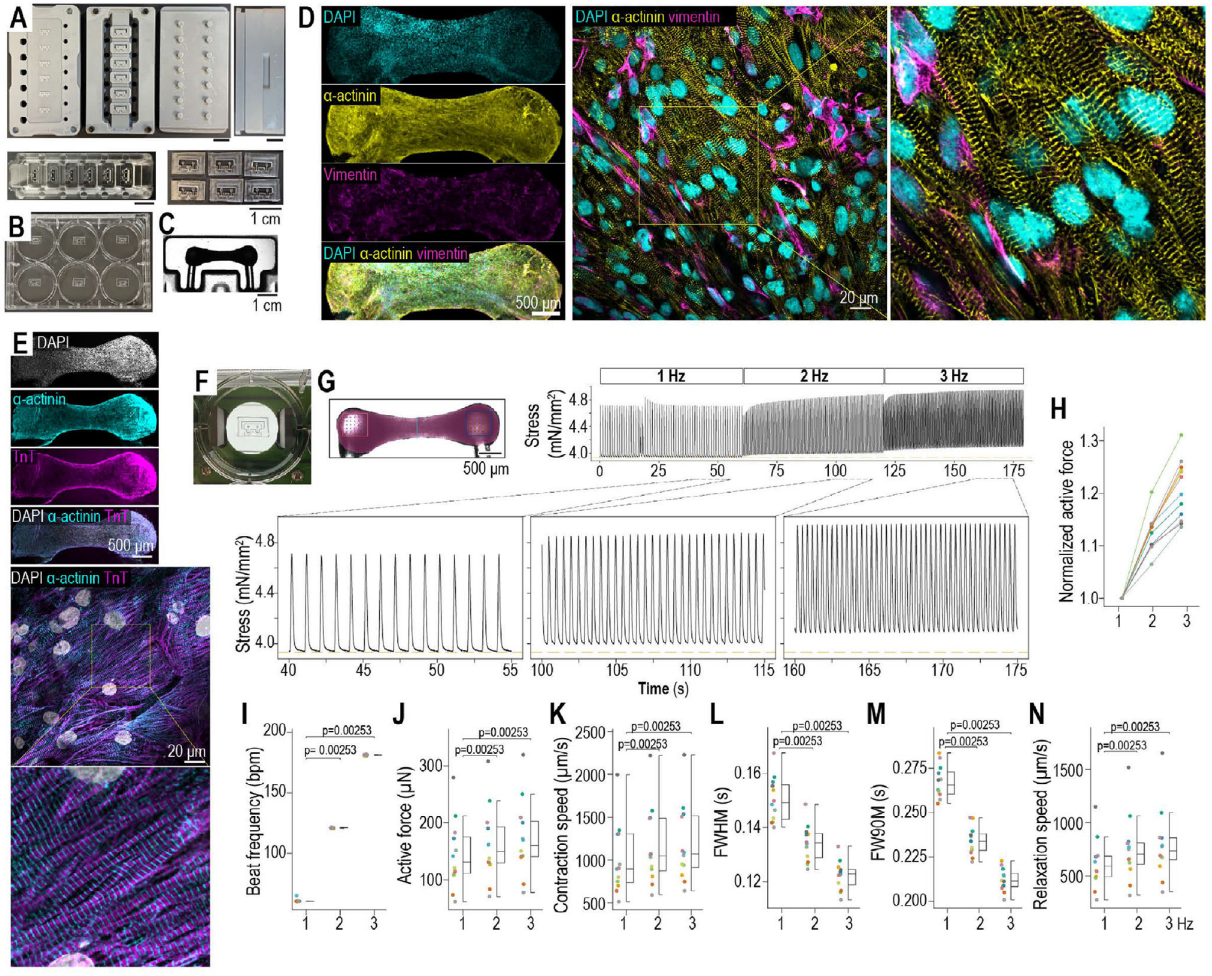
Reproducible fabrication of EHTs using a modified milliPillar platform. (A) Modified milliPillar molds were 3D‐printed in temperature‐resistant resin and used to cast PDMS. (B) Individual modules were attached to multi‐well dishes, increasing flexibility and compatibility with standard electrophysiology and cell culture workflows. (C) Representative brightfield image of an EHT 1 month after plating. (D) Confocal section stained for DAPI, α‐actinin, and vimentin; magnified insert shows longitudinal cardiomyocyte alignment with interspersed fibroblasts (vimentin‐positive). (E) Confocal section stained for DAPI, α‐actinin, and troponin T (TnT). Magnified insert reveals aligned sarcomeric banding. (F) An IonOptix C‐Pace insert was used to deliver field electrical stimulation. (G) Contractility was assessed from 200‐fps brightfield videos under stimulation at 37°C in maturation media; the final 15 s of each pacing frequency plus a 5 s padding were analyzed with BeatProfiler. A positive force‐frequency relationship was observed in all EHTs (H). (I–N) Quantifications are shown as box plots (median, interquartile range (IQR), and 1.5X IQR whiskers) overlaid with scatter points for N = 12 independent EHTs from 2 batches, paired by frequency. Several endpoints were non‐normally distributed (Shapiro‐Wilk test), so the Friedman test followed by a paired Wilcoxon test versus the 1 Hz condition was applied for (I‐N) Repeated p‐values reflect identical signed‐rank patterns across outcomes.

We fabricated EHTs by mixing hiPSC‐CMs (∼95% pure, Figure S3) and primary human ventricular cardiac fibroblasts (Lonza) at a 3:1 ratio in collagen hydrogel, as previously validated [15, 17, 23, 24]. Using the original protocol [17], we initially detached tissues with a 26‐gauge needle at 1 and 24 h post‐plating; in our hands, however, needle scoring slowed plating, introduced user‐ and batch‐dependent variability, increased assembly failures, and left PDMS strands in the tissue. To eliminate manual scoring, we applied corona plasma treatment followed by KnockOut Serum Replacement (KOSR) blocking to reduce PDMS‐hydrogel adhesion (Table S3). With this conditioning, 100% of EHTs self‐detached spontaneously within 24 h post‐plating (Figure S2F). We then transitioned to DMEM‐based metabolic maturation media supplemented with KOSR as a lipid source, with additional supplements as described [25]. After transition to metabolic maturation media, self‐assembly began within 24 h, and spontaneous contractions appeared after ∼72 h. One‐week EHT successful assembly was 79 ± 9.3% (four batches). Failures were exclusively unilateral detachment of the compacting tissue from one of the two pillar heads at the onset of synchronized contraction, producing spheroidal tissues around the still‐anchored pillar; the tissue itself remained viable and contractile but could not be used for the optical mapping assays that follow. No further tissue rupture or failure to develop contractile activity was observed in bipolarly anchored tissues.

To improve electrophysiological responses, we applied hormonal maturation with triiodothyronine (T3) and dexamethasone for 2 weeks [26] starting at the end of week 1, after which EHTs returned to DMEM‐based metabolic maturation media until experimentation. Transcriptional maturation was assessed by a 34‐gene qPCR panel comparing EHTs at day 7 versus day 23 of culture (n = 5 EHTs per timepoint; Figure S4 and Table S4). Coordinate up‐regulation was observed across ion‐channel (SCN5A, KCNJ2, KCNJ12), ECC (ATP2A2, PLN, RYR2, JPH2, CAV3), sarcomeric (MYH6/7, MYL2/7, MYBPC3, TNNI3, TNNT2, TCAP, TTN, VCL, ACTC1), and OXPHOS‐complex (NDUFB8, SDHB, UQCRH, COX5A, ATP5F1A) transcripts, with isoform‐selective shifts toward the adult ventricular program (MYL2/MYL7, TNNI3/TNNI1).

To validate EHT organization and function, we immunostained and measured contractility in 1‐month‐old EHTs. Immunostaining confirmed longitudinal cardiomyocyte alignment with interspersed, elongated fibroblasts, resembling the anisotropic organization of adult myocardium (Figure 1D,E). To evaluate Cx43 organization at the protein level, we performed confocal immunostaining with troponin I counterstain (Figure S5), which showed Cx43 signal predominantly at the sarcolemma surface with some residual lateralization. For contractility assays, we paced EHTs in culture media at 1, 2, and 3 Hz for 60 sec intervals using an IonOptix C‐Pace, with brightfield recordings at 200 frames per second (Figure 1F). Contractility and kinetics were assessed using BeatProfiler [27], and Figure 1G shows successful pillar head detection (Video S2). EHTs exhibited a positive force‐frequency relationship with consistent 1:1 capture and a failure rate of <5% at 1 Hz stimulation (Figure 1G–I). Active force and contraction/relaxation kinetics were comparable to previously published milliPillar data (Figure 1J–N) [17, 23, 27]. These data indicate that EHTs reach a stable, reproducible contractile phenotype by 1 month, prompting electrophysiological characterization at this time point.

### 2.2 Development of a Reproducible, High‐Resolution, Simultaneous Voltage and Ca^2+^ Optical Mapping System

High spatiotemporal‐resolution optical mapping requires unobstructed optical access to the entire tissue preparation, minimized motion artifacts, stable temperature control, and near‐constant perfusion to maintain tissue viability. To meet these requirements, we developed a simple, adaptable imaging workflow for milliPillar‐based EHT modules. Dye loading was optimized to maximize signal‐to‐noise ratio while minimizing phototoxicity; the potentiometric dye RH‐237 (30 µM) and Ca^2+^‐sensitive dye Rhod‐2 AM (10 µM) were co‐loaded with 0.1% Pluronic F‐127 in blebbistatin‐containing (5 µM) Tyrode's Solution. Loading for 20 min at 37°C yielded robust voltage and Ca^2+^ signals, while longer incubations increased phototoxicity and rendered tissues unresponsive to electrical pacing.

We used an Arduino‐controlled transistor‐transistor logic (TTL) signal generator to control electrical stimulation, LED illumination, camera acquisition, and to pause perfusion immediately before and during recording to minimize motion artifacts. We performed simultaneous dual‐channel optical mapping using a SciMedia MiCAM03 system (Figure 2A,B). To secure the tissue during imaging, we designed a 3D‐printed perfusion stage to stabilize individual milliPillar modules, maintain a constant level of physiological buffer at a constant temperature, and provide electrical stimulation to the tissues. A centrally positioned divot with adjustable EHT holders secures milliPillar modules. At the same time, stable temperatures are maintained by a dual‐channel ThermoClamp (AutoMate Scientific) heating perfused buffer and the imaging chamber (Figure 2C). Electrical stimulation was delivered via platinum wires mounted on a slidable holder, enabling point stimulation of the right EHT head (Figure 2D, STL Files 2–7).

**FIGURE 2:**
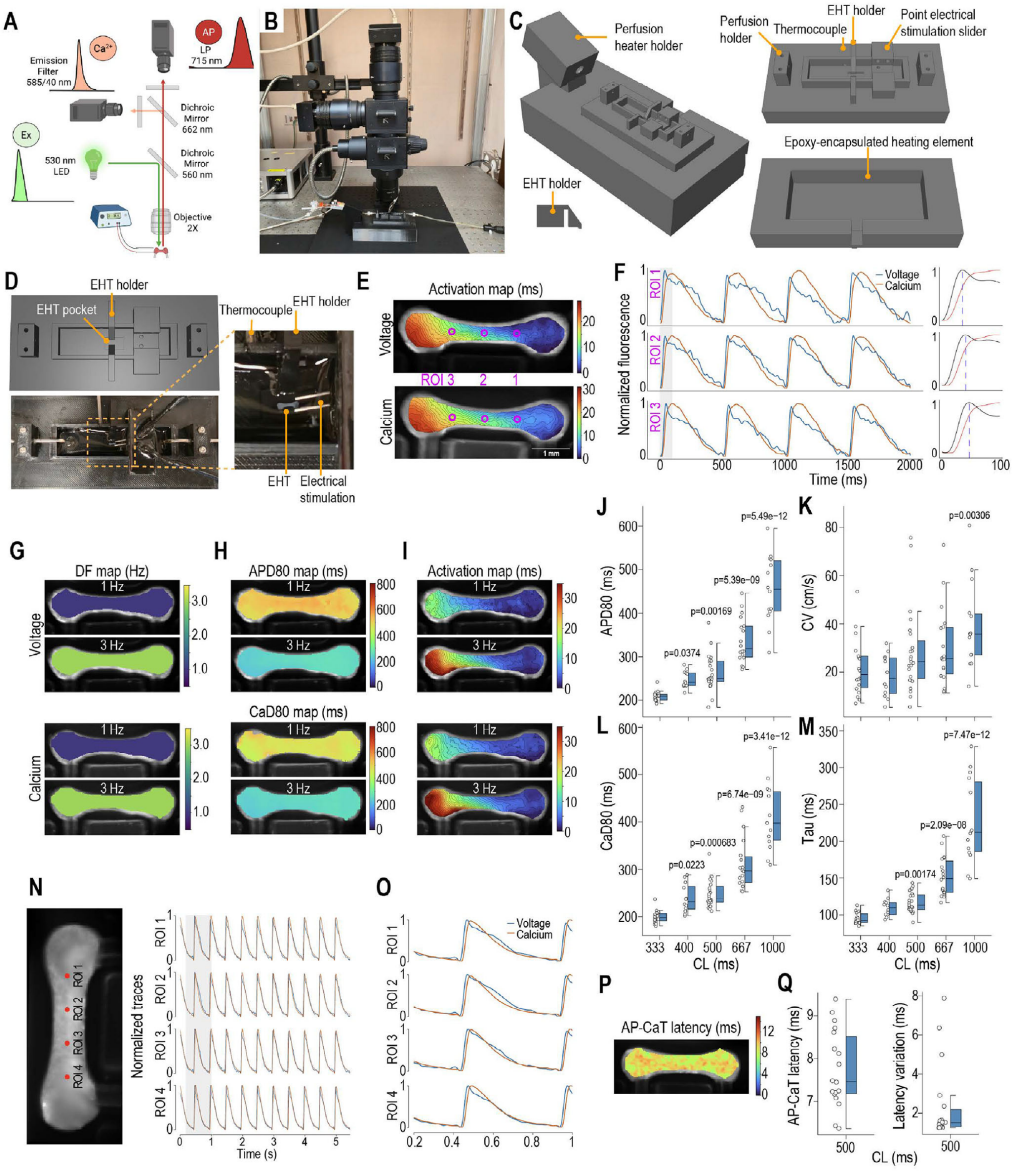
Optical mapping setup and EHT electrophysiology. (A) Schematic representation. “Created in BioRender. Altamirano, F. (2026) https://BioRender.com/harfohp”. (B) Photograph showing LED, mesoscope, camera, and optics. (C) 3D rendering of STL files for the imaging stage: a perfusion heater holder and epoxy‐encapsulated heating elements maintain temperature during recording, while EHT holders and pocket minimize motion and flotation. An adjustable slider with two platinum wires provides point‐ or field‐electrical stimulation. (D) Top‐down view showing milliPillar module positioning for a point‐electrical stimulation experiment. (E) Ensemble voltage (top) and Ca^2+^ (bottom) activation maps from a representative EHT paced at 2 Hz. Purple circles denote ROIs shown in panel (F) showing normalized voltage (blue) and Ca^2+^ (orange) signals illustrating consistent propagation, with voltage preceding Ca^2+^. (G–I) Dominant frequency (DF) and ensemble APD_80_, CaD_80,_ and activation maps at 1 and 3 Hz. (J–M) Quantifications of APD_80_/CV for voltage and CaD_80_/tau for Ca^2+^ signals reveal rate‐dependent restitution curves. Data are expressed as cycle length (CL = (1/frequency) × 1000 ms). N = 11–18 EHTs from 4 batches. Several endpoints were non‐normally distributed (Shapiro‐Wilk), thus nonparametric testing was used for consistency. Because not all tissues were recorded at identical pacing frequencies (some experiments included 1–3 Hz with or without 0.5 intervals or had one excluded timepoint due to noise or artifacts), comparisons employed the Kruskal‐Wallis test with Dunn's post hoc test versus the 333 ms CL condition. (N, O) Voltage (blue) and Ca^2+^ (orange) traces from ROIs across a representative EHT paced at 2 Hz; the magnified region (0.2‐1 s) on the right shows AP‐CaT latency across multiple ROIs. (P) AP‐CaT latency map generated with KairoSight shows uniform latency across the representative EHT from N‐O. (Q) Mean AP‐CaT latency and variation (SD) indicate highly consistent ECC efficiency across tissues. Latency was calculated from all recordings presented in (J–M) obtained at 2 Hz (500 ms CL; N = 18 EHTs, 4 batches). All quantifications are shown as box plots (median, IQR, and 1.5X IQR whiskers) with overlaid scatter points.

Simultaneous voltage and Ca^2+^ recordings achieved a temporal resolution of 1 ms and a spatial resolution of ∼22 µm. Signal processing was performed using spatial and temporal denoising, asymmetric least‐squares baseline correction, and 0–1 normalization (see Methods). EHTs exhibited uniform voltage and Ca^2+^ wave propagation from the right EHT head (point stimulation site) toward the left side, and a consistent action potential (AP) upstroke preceding the rise in Ca^2+^ transient (CaT) at every measured location (Figure 2E,F; Videos S3 and S4).

### 2.3 Rate‐Dependent Restitution Curves and Fast AP‐Ca^2+^ Coupling in EHTs

Voltage and Ca^2+^ signals revealed a homogeneous tissue response to 1–3 Hz pacing and expected physiological frequency‐dependent changes (restitution curves) in action potential duration at 80% repolarization (APD_80_), head‐to‐head longitudinal conduction velocity (CV), Ca^2+^ duration at 80% baseline (CaD_80_), and CaT decay time constant (Tau); with all parameters decreasing as cycle length (CL = (1/frequency) × 1000) shortened, confirming rate‐dependent electrophysiological adaptation (Figure 2G–M). At a 1000 ms cycle length, APD_80_ fell within the range reported for adult human ventricular myocardium, whereas longitudinal conduction velocity was approximately half of reported adult human values (see Discussion)

We also spatially evaluated ECC efficiency by measuring the latency between AP and CaT upstrokes (AP‐CaT Latency) for the first beat of the recordings obtained at CL 500 ms (Figure 2N,O). Mean AP‐CaT latency of 7.7 ± 0.2 ms at a 500 ms CL was of comparable magnitude to values reported for adult human ventricular myocardium [28], supporting physiologically relevant ECC timings in EHTs (Figure 2P,Q). Collectively, these findings indicate partial electrophysiological maturation; further evaluation of structural and metabolic maturation parameters is needed, as addressed in the Discussion.

### 2.4 Pharmacological Sensitivity to E4031

We performed pharmacological validation with the selective hERG blocker E‐4031 (100 nM) at a baseline pacing CL of 500 ms (2 Hz). Exposure to E‐4031 produced a reproducible prolongation of APD_80_ (Figure S6). Ensemble activation maps showed modest, non‐significant elongation of activation time after drug treatment. Representative voltage traces from individual regions of interest illustrate the clear AP prolongation induced by E‐4031.

### 2.5 Modeling Acquired Long‐QT Syndrome (aLQTS) in EHTs

VT arises from the interaction of arrhythmogenic triggers with a permissive tissue substrate. To interrogate the trigger arm of this framework in EHTs, we modeled aLQTS, a clinically important source of triggered activity, increased risk of life‐threatening ventricular arrhythmias, and sudden cardiac death [29]. The combination of high‐dose hERG inhibition (500 nM E‐4031) with hypokalemia and hypomagnesemia is an established pro‐arrhythmic intervention that reliably induces VT‐like phenomena in animal models of aLQTS [18, 19, 30]. We used this model to determine whether EHT reproduces these arrhythmogenic alterations at the tissue scale (Figure 3A). A modified Tyrode's solution containing 2 mM K^+^ and 0.5 mM Mg^2+^ (reductions of 60% and 50%, respectively) supplemented with 500 nM E‐4031 was used to induce the aLQTS condition.

**FIGURE 3:**
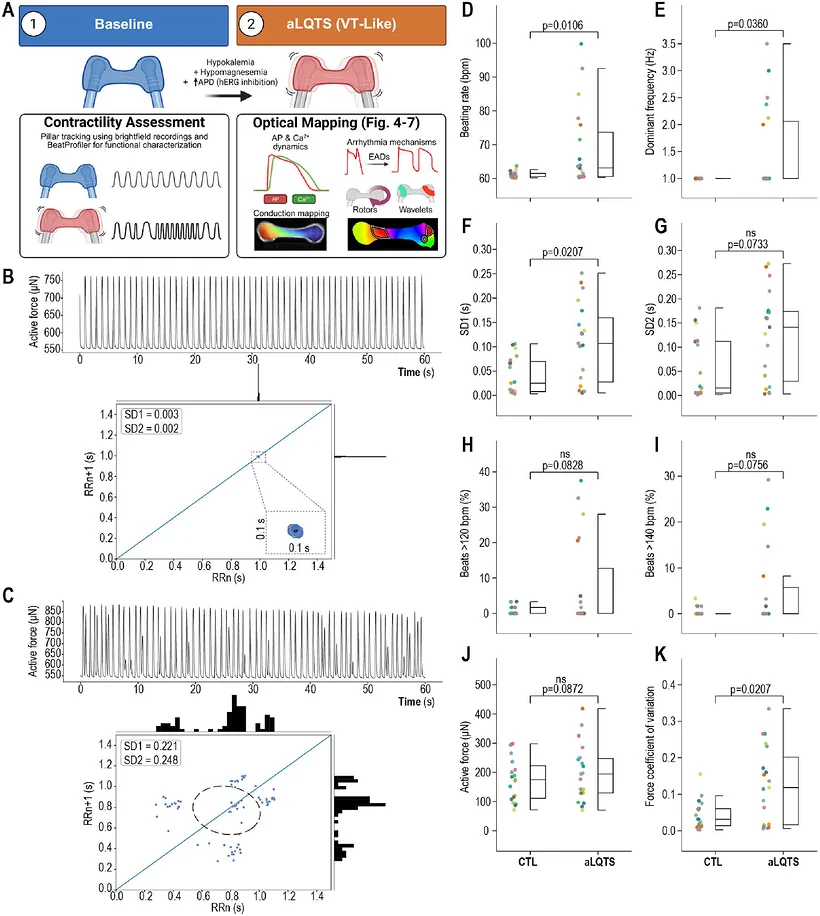
Generation of VT‐like condition in EHTs. High‐dose E‑4031 (500 nM) combined with low extracellular K^+^ and Mg^2+^ (aLQTS) created a proarrhythmic substrate that emerged within approximately 1 min and persisted for the duration of the observation period (<5 min). (A) Diagram of contractility and optical mapping assessments under baseline and aLQTS conditions. “Created in BioRender. Altamirano, F. (2026) https://BioRender.com/btrdquu”. (B) Representative active force trace and Poincaré plot from a control (CTL) EHT at 1 Hz pacing demonstrates 1:1 capture and consistent beat‐to‐beat synchrony. (C) Representative active force trace and Poincaré plot from an EHT following acute exposure to low K^+^/Mg^2+^ modified Tyrode with 500 nM E‐4031 (aLQTS condition) shows transient increases in beating rate and beat‐to‐beat variability. (D–K) Summary quantifications of N = 19 EHTs (4 batches) recorded under CTL and then aLQTS conditions. All data are shown as box plots (median, IQR, and 1.5X IQR whiskers) with overlaid scatter points. Paired data were compared using a two‐sided paired Wilcoxon signed‐rank test because several metrics showed non‐normal paired differences.

Field electrical stimulation delivered by C‐Pace (IonOptix) produced regular, synchronous contractions at 1 Hz in culture media (Figure 3B, CTL condition). The same tissues were then exposed to low K^+^/Mg^2+^ plus E‐4031 solution and, within 1 min, exhibited periods of rate acceleration beyond the pacing frequency, resulting in variable force generation and increased inter‐beat variability (Figure 3C, aLQTS condition). We observed significant increases in beating rate, dominant frequency, and inter‐beat variability (SD1 and SD2 Poincaré analysis) (Figure 3D–I). While average force generation was preserved in aLQTS, the coefficient of force variation increased significantly, likely due to arrhythmic beating behavior (Figure 3J,K). These findings suggest that combining hERG inhibition with hypokalemia and hypomagnesemia initiates VT‐like episodes in EHTs (aLQTS condition); therefore, we performed optical mapping studies to dissect the underlying mechanisms.

### 2.6 Optical Mapping Resolves the aLQTS‐Driven VT‐Like Phenotype

Dual voltage and Ca^2+^ optical mapping was performed on EHTs from two hiPSC lines under 1.0 or 1.5 Hz field stimulation; recordings at both pacing rates were pooled given equivalent arrhythmic behavior (N = 27 CTL and N = 28 aLQTS EHTs; six batches total). CTL tissues synchronized to pacing with minor spontaneous deviations (< 5%), whereas aLQTS EHTs randomly accelerated beyond the commanded rate and transiently became unresponsive to pacing. Time spent beating at >120 and >140 bpm, calculated from all recordings, was significantly increased in aLQTS vs CTL (Figure 4A,B).

**FIGURE 4:**
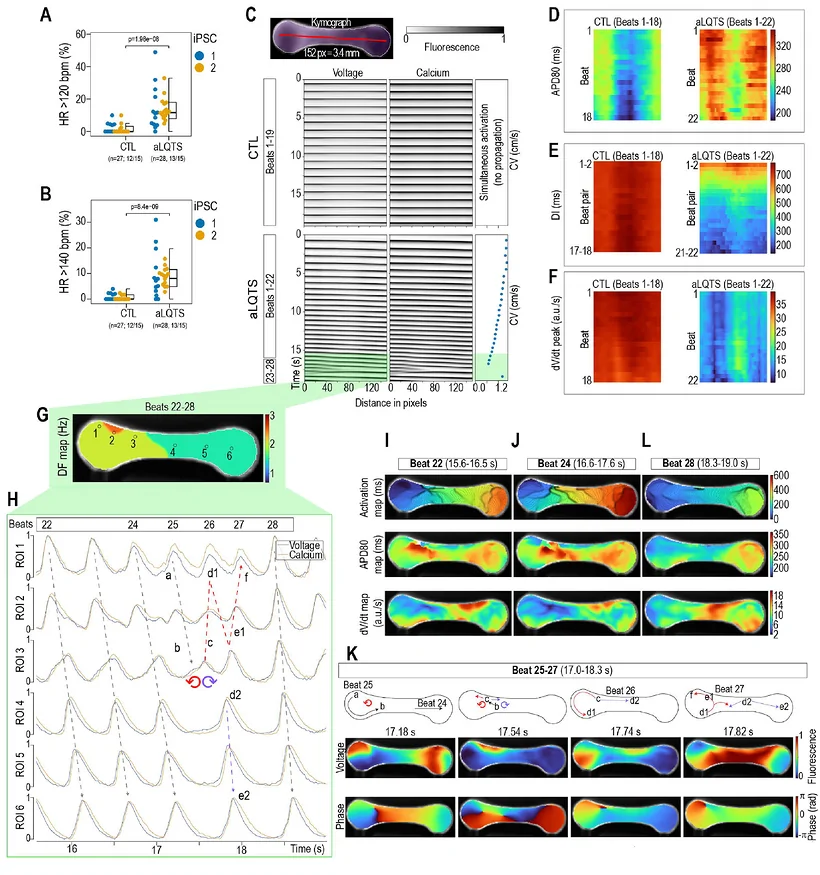
aLQTS condition prolongs APD_80_, increases dispersion, and promotes wavebreak, reentry, and a VT‐like phenotype in EHTs. Multiple dual voltage and Ca^2+^ optical mapping recordings were obtained per condition. Fraction of recording time spent above 120 (A) and 140 (B) bpm for EHTs paced at 1.0 Hz. Five EHTs from iPSC line 1 were paced at 1.5 Hz; these recordings were pooled with the 1.0 Hz data because the results were comparable. Shapiro‐Wilk indicated non‐normality for several distributions; unpaired two‐sided Mann‐Whitney U tests were applied (CTL N = 27, aLQTS = 28, from 4 batches for iPSC 1 and 2 batches for iPSC2s). Box‐and‐whisker plots show median and interquartile range; dots are individual EHTs. C. Voltage (left) and Ca^2+^ (right) kymographs from a CTL (top) and aLQTS (bottom) recording showing simultaneous activation under pacing in the CTL and progressive loss of capture, beat acceleration, and slowed CV under aLQTS (Video S5 and S6). D‐F. Heatmaps showing pixel‐wise changes across beats for APD_80_, DI, and dV/dt from a representative EHT under CTL (left) and aLQTS (right) conditions. APD_80_ becomes prolonged and spatiotemporal localized heterogeneity increases under aLQTS condition, while DI shortens and dV/dt slows, likewise becoming more heterogeneous. G. Dominant frequency (DF) maps from beats 22–28 outlined in green highlight regions of increased frequency leading to wavebreak and reentry across the EHT. H. ROI‐based voltage and Ca^2+^ traces transition from slow but continuous propagation to slowed conduction causing conduction block and reentry before returning to slow but continuous propagation. Activation, APD_80_, and dV/dt maps for beats 22 (I) and 24 (J) show decreased CV and increased spatiotemporal APD_80_ heterogeneity, producing dV/dt minima and conduction delays preceding wavebreak. K. Diagrams (top), voltage fluorescence (middle), and phase (bottom) maps for beats 25–27. Signals emerge from the left head, move in a downward circular motion (a‐b), slow down within the head‐neck space (b‐c), and bifurcate onto areas that exhibited low APD (prior beat, J), while avoiding high‐APD areas. Two PS/rotor arise: one moving leftward, initiating a second rotation in the left head (c‐d1), and the other propagating on the upper side towards the right head (c‐d2). After rotation, the left wave (d1‐e1) collided with a bidirectional wave initiated near the right neck (from d2), and both wavefronts extinguished shortly after. These events lead to the restoration of continuous but slow propagation by beat 28 (L), the dissipation of APD gradients, and the normalization of dV/dt (K). iPSC line 1, Gibco Episomal iPSC Line; A18945; iPSC line 2, Axol differentiated cardiomyocytes, hiPSC‐CMs; AXCC‐VC01.

We used kymographs to evaluate beat‐resolved electrophysiology and propagation dynamics along the longitudinal axis in CTL and aLQTS recordings. A representative CTL recording shows activation in straight lines, consistent with synchronized capture throughout the entire longitudinal axis due to field stimulation (Figure 4C and Video S5). After switching to low K^+^/Mg^+2^ plus E‐4031 (aLQTS), tissues progressively lost pacing capture, exhibited EADs, accelerating and decelerating bursts, and developed spatiotemporally fragmented activation consistent with wavebreak and transient reentry (Figure 4C). Spatial APD heterogeneity can promote wavebreak, which occurs when the AP wavefront fails to propagate in regions of cardiac tissue that have not fully recovered from previous excitation. When a wavebreak occurs in a localized area, it can lead to reentry and fibrillation [31]. The representative aLQTS recording shown in Figure 4C displays an activation starting from the top left corner of the EHT that progressively slows down (CV from 1.6 to 0.7 cm/s) as beating frequency accelerates before wavebreak and partial recovery toward the end of the recording (Video S6 shows events occurring between 15–19 sec). We used kymographs to quantify beat‐to‐beat changes in APD_80_, DI, and dV/dt, presented as heatmaps with distance along the tissue on the x‐axis and successive beats on the y‐axis. Under CTL conditions, EHTs showed a clear APD gradient that is prolonged toward the heads, but stable beat‐to‐beat APD_80_, consistent with 1:1 capture during field electrical stimulation (Figure 4D, left panel). In contrast, the aLQTS condition shows APD prolongation with more severe regional APD_80_ gradients that evolved over the recording period (Figure 4D, right panel). DI and dV/dt, which are highly consistent under CTL conditions, become spatially and temporally variable in the aLQTS condition (Figure 4E,F). Across recordings, conduction block consistently occurred in regions with pronounced APD prolongation and depressed dV/dt, suggesting repolarization delay as a potential mechanistic substrate for the functional conduction barriers observed under aLQTS conditions.

Dominant frequency mapping during activation fragmentation (beats 22–28 in the representative aLQTS recording) revealed localized high‐frequency activity toward the left EHT head, where frequencies rose to 2–2.5 Hz (Figure 4G). Voltage and Ca^2+^ traces in ROIs across the frequency gradient demonstrate a transition from organized wavefront propagation to regions of conduction block, accompanied by transient PS and rotors during wavebreak‐associated beats (Figure 4H and Video S6). Activation maps show progressively delayed activation times (from ∼200 during initial beats to ∼600 ms on beat 24), with larger APD_80_ gradients around the left EHT head. These changes coincided with local minima in dV/dt around the left EHT head on the beat preceding conduction block, wavebreak, and rotor formation (Figure 4I,J). During wavebreak, AP propagation avoided areas that had longer APD_80_ and low dV/dt (Figure 4J), resulting in a rotor in the left head. In contrast, on the right, propagation entered the EHT shaft, generating a beat that merged with the left‐head rotor (Figure 4K). By beat 28, a more organized propagation pattern was restored with faster activation and dissipation of APD_80_ and dV/dt gradients around the left EHT head (Figure 4L and Video S6).

Prior work in animal models has shown that the combination, but neither component alone, drives VT‐like arrhythmias [19, 30]. Optical mapping of a sequential three‐condition exposure in EHTs (Tyrode → low K^+^/Mg^2+^ → low K^+^/Mg^2+^ + E‐4031, N = 6 tissues, matched 1 Hz pacing) was consistent with this dependence: neither Tyrode's baseline nor low K^+^/Mg^2+^ alone exhibited VT‐like episodes or rotors, whereas the combined aLQTS condition triggered rotors in every tested tissue (0/6, 0/6, and 6/6, respectively; Figure S7).

### 2.7 Early Afterdepolarizations in aLQTS EHTs Exhibit a Spectrum of Propagation Phenotypes and Contribute to Functional Conduction Barriers

Repolarization instability in the aLQTS condition manifested as early afterdepolarizations (EADs) that displayed heterogeneous propagation behaviors across regions and beats within the same tissue. Simultaneous dual‐channel optical mapping of a representative aLQTS EHT revealed three distinct EAD phenotypes: (i) subthreshold EADs that failed to depolarize the surrounding tissue and remained confined to their site of origin; (ii) attenuated EADs that initiated a local depolarization wavefront but decayed before reaching neighboring ROIs; and (iii) fully propagating EADs that generated triggered activity (TA) spreading across the tissue as an ectopic beat (Figure 5 and Video S7). Every voltage EAD was temporally coincident with a CaD prolongation or secondary‐hump component in the same ROI, and no isolated diastolic Ca^2+^ events were observed in the absence of a preceding voltage upstroke, arguing against a DAD‐mediated mechanism.

**FIGURE 5:**
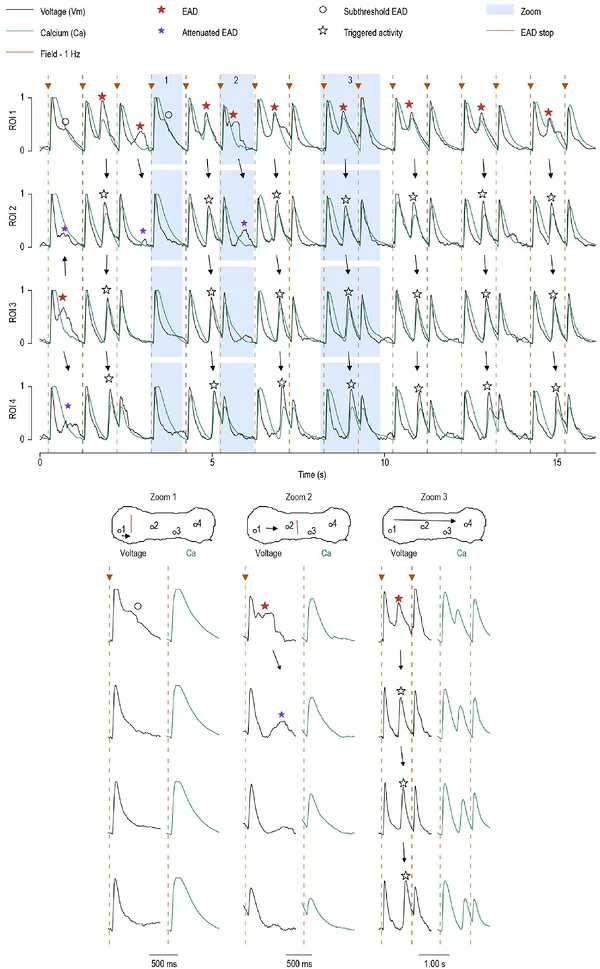
Heterogeneous EAD behavior and propagation in an aLQTS. *Top panel*, Simultaneous dual optical mapping of transmembrane voltage (black) and intracellular calcium (Ca, green) recorded from four regions of interest (ROI 1–4) of aLQTS EHT paced at 1 Hz (field stimuli timestamps indicated by orange dashed lines and arrowheads). Traces are min‐max normalized to the first‐99th percentile range (0‐1). EADs were absent in control and emerged after aLQTS, displaying a spectrum of behaviors across ROIs and beats: subthreshold EADs that fail to depolarize the tissue (open circles), attenuated EADs that propagate locally but decay before reaching neighboring regions (red‐purple stars), and EADs that give rise to triggered activity propagating across the tissue (red star into open stars). Black arrows between ROI rows denote the direction of propagation. Blue shaded windows (1–3) mark the segments expanded on the bottom. *Bottom panel*, Zoom 1–3: magnified Voltage (black) and Ca (green) traces from the four ROIs for each highlighted event, together with a schematic of the EHT indicating the four ROI positions (o1–o4), the propagation direction (black arrow), and, when applicable, the site of conduction block (red bar). Scale bars: 500 ms (Zoom 1, Zoom 2), 1.00 s (Zoom 3). Refer to Video S7. iPSC line 2, Axol differentiated cardiomyocytes, hiPSC‐CMs; AXCC‐VC01.

EAD‐driven TA propagated in two spatial patterns: locally (attenuated EADs that decayed near their site of origin) or from one EHT head, in the latter case sometimes colliding with wavefronts propagating from the opposite head (Figure S8). EADs are also commonly co‐localized with regions of APD dispersion and dV/dt depression immediately before rotor formation (as shown in Figures 4 and 6, see below), suggesting that triggered depolarizations both initiate tachyarrhythmias and contribute to the functional conduction barriers that stabilize reentrant activity.

**FIGURE 6:**
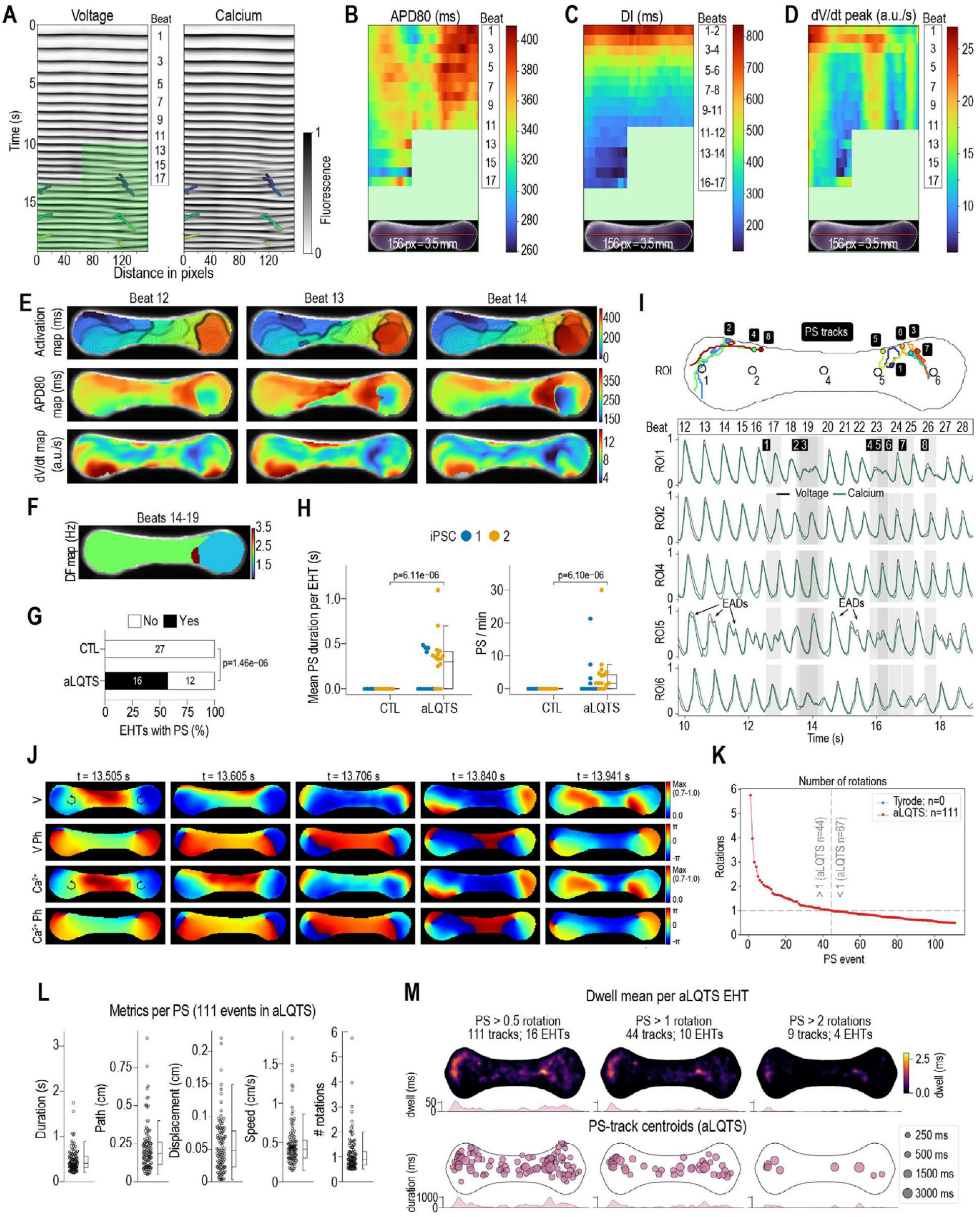
Phase singularities and rotor dynamics in aLQTS‐condition EHTs. (A) Voltage (left) and Ca^2+^ (right) kymographs with PS tracks overlay from a second representative aLQTS recording with VT‐like phenotype during field pacing at 1 Hz. Continuous propagation was analyzed using kymographs, and areas with signal fragmentation were masked (green) and resolved with ROI‐based trace plots (see below). (B) Pixel‐wise longitudinal APD_80_ heatmap demonstrates APD gradients between EHT heads and shaft. Prior to reentry, APD dispersion within the heads progressively increased as DI progressively shortened during spontaneous beating acceleration (C). (D) Areas with reduced excitability (dV/dt minima) increased over time near the EHT head‐neck joints and preceded the formation of PS/rotors. (E) Activation time, APD_80_, and dV/dt maps for beats 12–14 show a progressive increase in activation time and circular propagation around the right EHT head. APD_80_ increased over time and progressively generated high‐low APD areas around the right EHT head, linking the initial slow and circular propagation to a reduction in dV/dt that preceded conduction block, wavebreak, and reentry. (F) Dominant frequency maps show areas beating at 3.5 Hz in the right EHT neck‐head joint caused by reentry driven by rotor formation in the right EHT head. (G) Proportion of CTL tissues (0/27) versus aLQTS (16/28: iPSC1 = 4/13;iPSC2 = 12/15) showing PS. Fisher's exact test was used. (H) Summary quantifications showing mean PS duration and PS/min. Unpaired two‐sided Mann‐Whitney U test was applied. Box‐and‐whisker plots show median and interquartile range; dots are individual EHTs. (I) *Top panel*, PS trajectories observed during the recording shown in A, starting on beat 15. *Bottom panel*, ROI‐based voltage (blue) and Ca^2+^ (orange) traces obtained from 6 ROIs between 10–19 s (masked green area shown in A) show APD prolongation with EADs in the areas preceding PS formation. Gray shading and numbered black boxes indicate when the PS‐tracking algorithm detected PS. (J) Sequential voltage (V) and Ca^2+^ frames with phase maps show simultaneous rotors in the left (counterclockwise) and right (clockwise) EHT heads, generating rotating wavefronts that converge at the center (Video S8). (K) Calculations of the number of rotations show that 44/111 PS exhibit >1 rotation. L. Summary quantifications of all PS events (111 total) across aLQTS EHTs. Box‐and‐whisker plots show median and interquartile range; dots are individual EHTs. (M) Anatomy‐normalized overlay of PS dwell time and centroids for all PS (>0.5 rotation), >1 and >2 rotations. Maps show predominant localization in the tissue head and neck regions. All quantifications except panel M were reported with box plots (median, IQR, and 1.5X IQR whiskers) with overlaid scatter points. Gibco Episomal iPSC Line; A18945; iPSC line 2, Axol differentiated cardiomyocytes, hiPSC‐CMs; AXCC‐VC01.

Across the aLQTS cohort, EADs occurred in 25/28 tissues and were absent from all CTL recordings. EAD occurrence and TA did not always progress to reentry, but they contributed to acceleration and the wavebreak transition (see 2.8). This dissociation between EAD presence and reentry indicates that rotor initiation requires additional substrate elements beyond the cellular trigger, including localized APD dispersion and dV/dt depression that establishes a functional conduction barrier at the reentry site.

### 2.8 Combined Acceleration, EADs, and Short‐Lived PS/Rotors in aLQTS EHTs

aLQTS EHTs exhibited recurrent, short‐lived rotors that co‐occurred with progressive acceleration and EAD‐driven triggered activity. In some recordings, these rotors were scarce and confined to a single tissue head (Figure 4K). In contrast, in other recordings, multiple rotors were observed and coincided with local increases in beating rate. Kymographs from a second representative aLQTS EHT (Figure 6A) showed a fragmented beating pattern along the longitudinal axis that coincides with PS and rotors on both the left and right EHT heads. Initially, APD_80_ was substantially longer but homogeneous in the right head, with persistent APD gradients across the tissue (Figure 6B). Interestingly, the right EHT head exhibited local APD gradients like the ones observed in Figure 4 before rotor initiation, whereas the left head exhibited APD alternans (alternating short‐long APD) together with local APD gradients before rotor initiation. DI shortened substantially during beat‐to‐beat spontaneous acceleration from ∼764 to ∼201 ms on the right side and ∼261 ms on the left side immediately before reentry (Figure 6C). Progressive regional reductions in upstroke velocity dV/dt were observed first around the right EHT neck and then around the left EHT neck, preceding rotor formation (Figure 6D).

To quantify these reentrant events, we computationally identified PS using a Hilbert‐transform winding‐number method, with a 1.8π winding threshold, adaptive periodicity gates, and rotation‐count validation (Suppl. Methods Section). We then manually verified each candidate track using per‐track phase‐map clips to exclude noise‐driven artifacts. Rotors were exclusively observed under aLQTS conditions: 16 of 28 aLQTS EHTs exhibited PS (iPSC1 4/13; iPSC2 12/15), whereas none of 27 CTL EHTs did (Figure 6G). Both mean PS duration and PS/min were significantly increased in aLQTS vs CTL (Figure 6H).

In the same representative aLQTS EHT (Figure 6A), PS trajectories reconstructed from the same recording are shown overlaid on ROI‐based voltage and Ca^2+^ traces from six ROIs (Figure 6I). EADs consistently emerged in the ROIs preceding each PS event, providing a within‐tissue temporal link between the cellular trigger (EAD) and the tissue‐scale rotor emergence. Half rotations started earlier on beat 14 and consolidated into PS on beat 17 (∼12.5 s), with two rotors (counterclockwise in the left head and clockwise in the right head) coexisting and generating wavefronts that converged at the tissue center (Figure 6J and Video S8).

Across all aLQTS EHTs, 111 PS/rotor events were detected, of which 44 completed more than one full rotation (Figure 6K). Per‐track metrics indicated a median duration of ∼0.4 s, a path length of ∼1.9 mm, an end‐to‐end displacement of ∼0.5 mm, and a speed of ∼0.4 cm/s (Figure 6L), consistent with localized, slow, and short‐lived rotor formation. When tissue geometry is normalized using xy principal‐component alignment, PS dwell‐time heatmaps and centroid overlays reveal PS enrichment within the head and neck segments, with travel paths frequently following head curvature (Figure 6M).

### 2.9 VF‐Like Fragmentation in a Subset of aLQTS EHTs

While rare (6/28 of aLQTS EHTs, 2 for iPSC1 and 4 for iPSC2), transient episodes of short‐lived, highly complex activation patterns characterized by the presence of multiple wavelets and PS were observed, with partial AP and Ca^2+^ wavefront segmentation consistent with chaotic signal resembling VF‐like episodes (Figure 7). Voltage and Ca^2+^ kymographs with overlaid PS traces demonstrate spatially separated, simultaneous activation at opposite ends of the tissue, generating up to 3 wavefronts or wavelets (Figure 7A,B and Video S9a iPSC1). These wavelets and PS coexisted throughout the recording (Figure 7C,D) but quickly resolved into a more organized VT‐like phenotype. Only 1/28 maintained a disorganized pattern throughout the recording (Video S9b iPSC2). Figure 6I,J illustrate an intermediate case in which two PS completed full rotations at opposite EHT heads before self‐terminating—a transitional phenotype between focal VT and the multi‐wavelet fragmentation described above.

**FIGURE 7:**
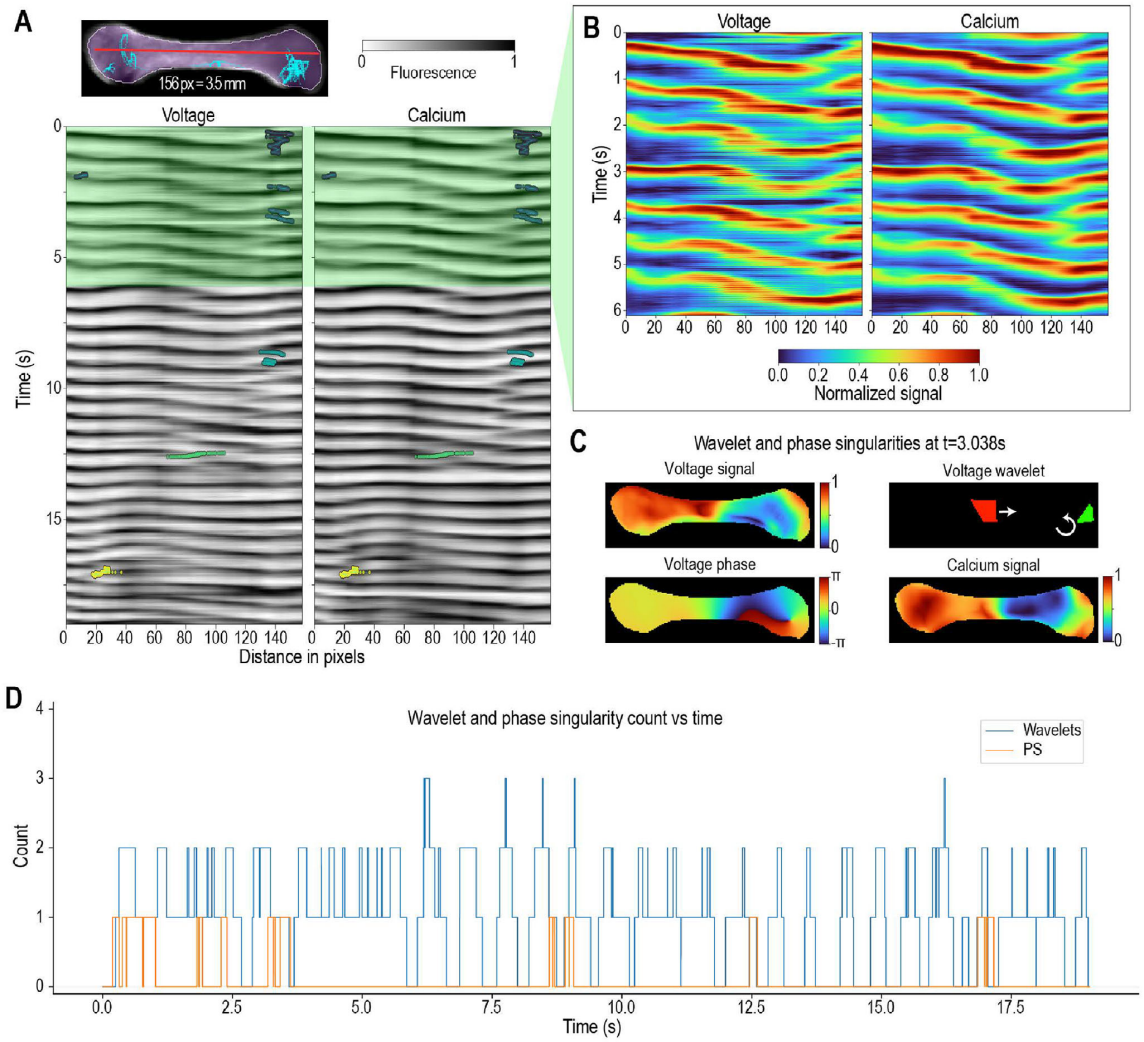
Highly complex activation patterns in a subset of aLQTS‐condition EHTs exhibit VF‐like fragmentation. (A) Voltage (left) and Ca^2+^ (right) kymograph with overlaid PS tracks from an aLQTS EHT. (B) Magnified view highlights the spatiotemporal heterogeneity of voltage and Ca^2+^ signals (Video S9a). (C) Voltage signal and phase map with highlighted voltage wavelets reveal abnormal activation coinciding with a PS and two simultaneous wavelets and signal heterogeneity. (D) Quantification of simultaneous wavelet and PS counts over time in the representative aLQTS EHT demonstrates VF‐like presence of multiple wavelets.

### 2.10 Focal Cryoinjury Generates a Spatial Substrate for Anatomically Anchored Reentry

The aLQTS results described above characterize one example of the trigger arm of ventricular arrhythmogenesis using a pharmacological/metabolic intervention on structurally uninjured tissue. To interrogate the complementary substrate arm – a fixed structural obstacle that anchors reentry immediately after cardiomyocyte loss to promote reentrant VT [20, 21, 22] – and to test the hypothesis that focal damage on EHT heads could promote anatomically anchored reentry, we used focal cryoinjury.

A separate cohort of 10 EHTs was imaged in standard Tyrode's solution (no aLQTS or electrolyte perturbation) both before and within 5 min after localized cryoinjury of the left tissue head, delivered with a liquid‐nitrogen‐chilled 1 mm copper pipe under a dissecting microscope while closely monitoring the freezing halo until *∼*50% of the surface head was frozen (Figure 8A); each tissue therefore served as its own pre‐injury control.

**FIGURE 8:**
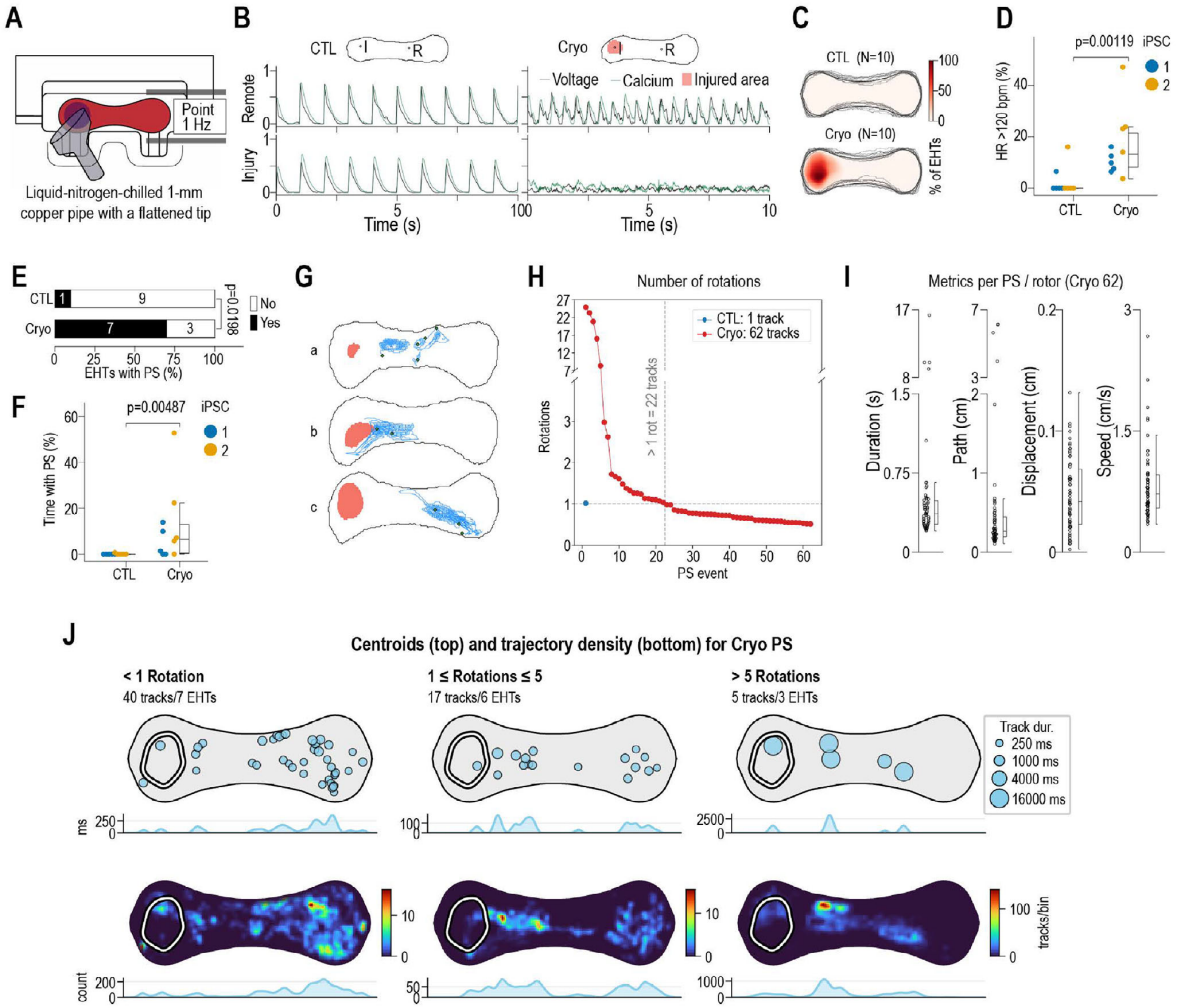
Cryoinjury (Cryo) in EHT heads generates a spatial substrate for reentry. (A) Dual voltage and calcium optical mapping was performed before and after Cryo. Cartoon showing experimental design. Cryo was achieved using a liquid nitrogen‐chilled 1 mm copper pipe. The tip was flattened to cover the surface between the silicone edge and the center of the left EHT head. One edge of the frozen pipe was positioned on the silicone and then gently tilted toward the EHT head until a freezing halo covering around 50% of the head was visible. Optical mapping was performed thereafter within a 5 min period. (B) Voltage and Calcium signals were denoised and normalized from 0 to the 70th percentile 70% to identify damaged areas. The left panel shows the tissue before and after (CTL and Cryo, respectively). (C) A combination of signal‐to‐noise ratio and peak amplitude was used to evaluate injured areas. 10/10 cryoinjured EHTs showed a damaged area within the left head. (D) Cryo promoted tachyarrhythmias characterized by events with HR >120 bpm during a 1 Hz (60 bpm) point stimulation pacing delivered on the right head. (E) Cryo increased (phase singularities) PS compared to CTL (Total 62 events; 1 CTL and 62 in Cryo). N = 10 EHTs per condition from two iPSC lines. (F) Percentage of time in PS between CTL and Cryo (N = 10 EHTs per condition). (G) PS trajectory tracks (light blue) in Cryo EHTs. Red represents the area with low SNR and amplitude that was used to mask the tissue and normalize each pixel 0–1 to avoid noise amplification. (H) Number of rotations for each PS detected in CTL and Cryo. A total of 22/62 tracks in Cryo and 1/1 in CTL had > 1 rotation. (I) Quantification of duration, path, displacement, and speed of Cryo tracks (J) For each Cryo PS track, centroids and trajectory density dwells were calculated for events with < 1 rotation, <1–5 rotations, and >5 rotations. Gibco Episomal iPSC Line; A18945; iPSC line 2, Axol differentiated cardiomyocytes, hiPSC‐CMs; AXCC‐VC01.

Denoised, 0–70th percentile‐normalized global voltage and Ca^2+^ signals identified damaged areas as regions with combined loss of signal‐to‐noise ratio and peak amplitude at the target head (Figure 8B). Across the cryoinjury cohort, 10/10 tissues showed a damaged area confined to the targeted left head (Figure 8C), with the injury mask spatially reproducible across preparations and both hiPSC lines. We used the cryoinjury mask for 0–1 normalization, which prevented noise amplification in injured areas.

Cryoinjury induced more tachyarrhythmia episodes with events above 120 bpm during 1 Hz right‐head point stimulation (Figure 8D) compared to pre‐injury CTL recordings. PS were detected in 7/10 Cryo EHTs (62 events) vs. 1/10 CTL (a single short‐lived event, Figure 8E), and the fraction of recording time containing at least one PS increased markedly (Figure 8F). Rotor tracks were localized preferentially to the border of the injury mask or around the entire tissue (Figure 8G a‐d and Videos S10–S12), consistent with anatomical anchoring of reentry at the damaged/healthy interface.

Track‐level analysis revealed that 22/62 cryoinjury tracks completed more than one full rotation (Figure 8H). Per‐track metrics indicated a median duration of ∼0.4 s, path length of ∼2.6 mm, end‐to‐end displacement of ∼0.4 mm, and speed of ∼0.7 cm/s (Figure 8I), with several rotors persisting across the entire recording window and reaching durations of 10 s or more. Because several such rotors were still present when the recording ended, all reported durations are recording‐limited lower bounds, and no upper bound on rotor lifetime can be established from these data. Anatomy‐normalized dwell heatmaps stratified by rotation count showed centroid clustering at the injury‐mask border and moving toward the other head (Figure 8J), a signature consistent with anatomical reentry.

## 3 Discussion

Non‐animal, human iPSC‐derived EHTs are gaining traction as translational platforms, yet their ability to recapitulate adult ventricular arrhythmia mechanisms remains unclear due to size limitations and tissue immaturity. To date, no study has provided high‐resolution, tissue‐level spatiotemporal electrophysiology to determine whether these models reproduce adult electrophysiological parameters or whether classic initiators and drivers of VT/VF manifest. We therefore adapted an open‐source EHT platform and implemented optical mapping at temporal and spatial resolution previously attainable only in whole hearts. Baseline measurements – rate‐dependent APD and Ca^2+^‐transient restitution, longitudinal conduction velocity, AP‐CaT coupling latency, and hERG pharmacological sensitivity – fell within adult human ventricular ranges for APD_80_ and AP‐CaT, and below them for conduction velocity. By imposing an aLQTS pro‐arrhythmic condition, we reproduced the sequence of substrate evolution underlying VT: localized APD dispersion, loss of excitability, conduction block, wavebreak, and rotor‐mediated reentry. Mapping revealed that repolarization instability creates transient “physical barriers” that anchor rotors and identifies head/neck anatomical hotspots serving as arrhythmic foci. Complementary focal cryoinjury created a fixed structural obstacle that anchored prolonged reentry at the injury border, in contrast to the transient functional rotors observed in the aLQTS condition. Together, these findings demonstrate that EHTs constitute a robust non‐animal platform for mechanistic arrhythmia research, capable of quantifying electrophysiological alterations and detecting major electrical events across a broad range of arrhythmogenic conditions.

### 3.1 Baseline Electrophysiology of EHTs Benchmarked Against Adult Ventricle

The measured baseline APD_80_ (446.9 ± 21.8 ms at 1000 ms CL) lies within the range reported for adult human ventricular myocardium under comparable conditions (305–500 ms; 18 values across nine studies [32, 33, 34, 35, 36, 37, 38, 39, 40], in the upper part of that range; in the last two studies the values at this cycle length are presented graphically and were read from the published figures. No adult human APD_80_ values acquired under comparable conditions were identified at shorter cycle lengths, so the rate dependence reported here cannot be benchmarked against human myocardium^353841^Similar APD_80_ values (∼424 ms at 1 Hz) have been reported for mature engineered cardiopatches [41], indicating that our millimeter‐scale EHTs achieve APD_80_ in the same range as more mature engineered human myocardium.

Longitudinal CV averaged ∼39 cm/s and fell to ∼21 cm/s at fastest pacing, approximately half of the longitudinal conduction velocity reported for adult human ventricle under comparable conditions (92 cm/s [42]; 77.1 cm/s [32]). This difference is maintained at faster rates: at a 500 ms cycle length, our 27.6 cm/s compares with approximately 65 cm/s read from the published figure of the same study [32]. Our values therefore lie between adult human transverse (22–28 cm/s; [32]) and adult human longitudinal conduction velocity. Reported velocities in human ventricular slice preparations vary by more than an order of magnitude [37, 40], and slice‐derived values were therefore not used for this comparison. ^454146^Notably, our CV values approximate those reported in mature engineered human myocardium (∼41 cm/s in large‐format EHT patches [43]; ∼28.5 cm/s in cardiopatches [41], indicating that the platform reaches CV consistent with advanced hiPSC‐EHTs even at the millimeter scale. The remaining gap relative to adult human tissue‐level CV is consistent with the residual Cx43 lateralization documented in our confocal immunostaining (Figure S5), where sarcolemmal Cx43 signal coexisted with lateralization in less organized regions. This feature is distinct from the additional, aLQTS‐driven conduction slowing that emerges only under pharmacologic perturbation.

AP‐CaT latency (7.7 ms) is consistent with the rapid ECC reported in non‐failing human ventricular myocardium (8.0–8.8 ms across transmural regions in coronary‐perfused LV wedges [28], and in line with values reported for adult mouse and rabbit hearts [30, 44, 45].

Collectively, these data demonstrate that millimeter‐scale EHTs reproduce baseline electrophysiological parameters within the range reported for the human ventricle, with conduction velocity approximately half of adult human longitudinal values. We do not claim equivalence to the adult human ventricle—the conduction deficit, incomplete Cx43 intercalated‐disk redistribution, and absence of Purkinje fibers all place tissue‐scale conduction below adult human values—but the baseline behavior is sufficient to support the pro‐arrhythmic mechanistic analyses that follow.

### 3.2 Pro‑Arrhythmic Substrate Generation and Mechanisms

Acute high‐dose hERG blockade with 500 nM E‑4031 under low K^+^/Mg^2+^ created a proarrhythmic substrate that emerged within approximately 1 min and persisted throughout the observation period (<5 min). E‐4031 alone (500 nM) further prolonged APD but did not induce VT‐like arrhythmias (data not shown), consistent with rabbit heart data, in which APD prolongation alone does not create a proarrhythmic substrate. When combined with low K^+^/Mg^2+^, E‐4031 induces heterogeneous repolarization, leading to VT/TdP [19, 30]. In this model, arrhythmias emerge from a combined failure of repolarization reserve and triggered substrates, where low extracellular K^+^ potentiates hERG blockage [46], reduces I_K1_ currents, and inhibits Na^+^/K^+^ ATPase, leading to repolarization heterogeneity and spontaneous ventricular firing [47], and low extracellular Mg^2+^ increases L‐type Ca^2+^ channel (LTCC) currents and SR Ca^2+^ release, amplifying afterdepolarization propensity in ventricular myocytes [48].

Our three‐condition sub‐study (Figure S7) formalizes the mechanistic decomposition: hypokalemia/hypomagnesemia in isolation was insufficient to trigger significant arrhythmia or rotors in any of the six tissues we tested, and rotors were triggered only after the addition of high‐dose E‐4031. This dose has been validated in numerous studies to promote repolarization alterations leading to aLQTS [18, 19, 30]. This matches the clinical observation that hypokalemia and hypomagnesemia are permissive rather than sufficient conditions for TdP; an additional QT‐prolonging insult (drug, ischemia, or genetic repolarization‐reserve defect) is generally required to convert the electrolyte substrate into a manifest arrhythmia [49].

The prevailing mechanistic view underlying VT/TdP in low K^+^/Mg^2+^ plus 500 nM E‐4031 has historically centered on EADs. Previous observations suggest that spontaneous Ca^2+^ events can precede voltage changes at EAD initiation sites, supporting a Ca^2+^‐overload contribution to EAD generation and polymorphic activity [18]. Building on this, Maruyama et al. reported that this phenomenon is dominated by phase‐3 EAD‐mediated triggered activity arising at borders between long‐ and short‐APD regions; in some TdP episodes, they observed EAD‐induced initiation of a reentrant rotor with a drifting core, emphasizing that APD gradients can couple triggered activity to reentry [19]. More recently, Alexander et al. proposed that triggered activity leading to premature ventricular contractions and TdP is better explained by LTCC reactivation originating in discrete regions with dynamically generated, extreme long‐short APD gradients, rather than by EADs [18]. They further suggested that low levels of LTCC blockade can suppress this activity. We observed no spontaneous increases in Ca^2+^ preceding voltage changes during aLQTS, providing no evidence for DAD‐mediated ectopic activity. However, our analyses primarily focused on voltage dynamics, and a contribution of secondary Ca^2+^ elevations to EAD formation, as demonstrated by Choi et al. and Chang et al. in rabbit aLQTS models [30, 50], or LTCC‐mediated triggered activity associated with steep repolarization gradients, as proposed by Alexander et al. [18], cannot be excluded and warrants dedicated Ca^2+^‐cycling analyses in future work.

As tissue started to accelerate, optical mapping showed increased localized dispersion of APD_80_, accompanied by progressive shortening of DI and a marked reduction in upstroke velocity, resulting in spatially heterogeneous repolarization gradients. These changes slowed conduction locally, promoted wavebreak, and enabled the emergence of short‑lived PS that manifested as focal rotors. Rotor trajectories were confined largely to the head‐neck junctions, regions where APD was longest and dV/dt lowest, suggesting that a combination of prolonged repolarization and impaired excitability is sufficient to generate re‑entrant circuits. The observations are consistent with classical mechanisms of ventricular tachycardia in which steep APD gradients [51] promote conduction block, wavebreak, and reentry. Intriguingly, sudden EAD formation preceded conduction block in regions with high and low APD gradients, creating “physical barriers” to wavebreak and rotor formation. Recent tissue‐scale modeling suggests that EADs in isolated cells are highly variable due to stochastic processes, leading to beat‐to‐beat APD variability; however, in cardiac tissue the fluctuations are largely dampened. EADs manifest as discontinuous alternans that lead to pronounced spatial heterogeneities, which are strongly arrhythmogenic, as the sudden onset of EADs on alternate beats in cardiac tissue promotes conduction block and reentry [52].

### 3.3 PS/Rotor Localization in EHTs

The concentration of PS/rotors near tissue heads/necks implicates EHT morphology and regional heterogeneity in the development of arrhythmogenic susceptibility that could arise from mechanical strain gradients, fiber orientation, tissue thickness, and other variables. In adult hearts, PS clustering occurs in a nonrandom spatial distribution and colocalizes with normal anatomic heterogeneities along epicardial vessels, ridges of endocardial trabeculae, and papillary muscle insertions [53]. These locations corresponded to areas of fiber apposition with different angulations and to intramural vessels. Similarly, rotational anisotropy and tissue boundaries play an important role in generating breakthrough waves during VF [54]. Mechanistically, fiber rotation induces curvature in scroll‐wave (3D rotor) fronts, weakening conduction and further facilitating wave break [55]. Additionally, dispersion of mechanical strain and electrical recovery have been described, underscoring mechanoelectrical feedback as an important part of arrhythmogenesis [56], which can cause stable spiral waves to deteriorate into turbulent wave patterns [57]. Interestingly, we observed APD gradients between the heads and shafts of EHTs, which under normal conditions did not trigger arrhythmic events and only generated rotors when a pro‐arrhythmic substrate was in place: APD dispersion within the head/neck leading to long‐ and short‐APD regions within the EHTs’ neck. These observations are consistent with anatomically realistic 3D ventricular models that incorporate physiological fiber orientation, which show that dynamical instability of propagation (e.g., restitution‐driven instability) is the primary trigger of wavebreak and VF. Cardiac geometry and anisotropic fibers modify the breakup threshold and shape filament folding, drift, and surface interactions, thereby increasing the likelihood and complexity of VF once instability is present. Conversely, when dynamical instability is sufficiently suppressed (e.g., by flattening restitution), organized electrical activity can persist despite structural heterogeneity. Thus, anatomy shapes fibrillatory dynamics but does not alone generate sustained VF without an unstable electrophysiological initiator [58].

The short‐lived VF‐like episodes observed in 6/28 aLQTS EHTs are notable as they display a surprisingly complex pattern (2–3 coexisting wavelets with intermittent phase singularities; Figures 6 and 7), indicating that proarrhythmic stressors can transiently drive multi‐wavelet dynamics even in EHTs. However, the short duration argues against sustained VF and supports wavebreaks that are generated but not maintained. Our EHTs are constrained to ∼4 mm in length, which may be insufficient to reproduce fibrillatory patterns due to space constraints in accordance with the critical mass hypothesis [59], and anatomical barriers along the transverse axis (∼0.5 mm) or at head‐shaft transitions may promote rotor annihilation around EHT heads. Moreover, for a rotor to remain stable, a critical minimum inter‐PS distance of approximately 4 mm is required during fibrillation in sheep hearts; if singularities of opposite chirality move closer than this threshold, they mutually annihilate [60].

### 3.4 Trigger and Substrate Arms of Tissue‐scale VT in EHTs

Our two interventions bracket the clinical trigger/substrate axis on a single tissue geometry. The aLQTS condition reproduces a trigger‐arm example: pharmacologic stress on structurally normal tissue generates EAD‐driven triggered activity and short‐lived functional reentry that self‐terminates within seconds under aLQTS. Cryoinjury reproduces one substrate arm: a fixed structural obstacle anchors reentry at the injury border, with some rotors persisting for the full recording window. This platform‐level dissociation indicates that rotor persistence on the milliPillar geometry is limited by substrate rather than by tissue mass. Cryoinjury is not a model of myocardial infarction or of ischemia–reperfusion injury, and we draw no parallel to post‐infarct or reperfusion arrhythmia, whose substrate is generated by surviving myocytes and by functional rather than structural conduction block [20, 61]. What the model reproduces is the acute arrhythmic consequence of a circumscribed region of cardiomyocyte loss, on a timescale of minutes [20]. The same workflow accepts commercially sourced hiPSC‐CM lines through the mass‐amplification protocol (as demonstrated here for line 2) and can be extended to patient‐specific LQTS or cardiomyopathy variants, positioning the platform to interrogate gene‐environment interactions in ventricular arrhythmia risk.

To benchmark rotor stability in our EHT models against human data, we compared per‐event rotation counts with those reported by Krummen et al. [62] during biventricular endocardial mapping of induced VF. In that study, ≥7 consecutive rotations separated sustained from self‐terminating VF; sustained episodes showed a mean maximum rotor stability of 17 ± 11 rotations (range 7–48), versus 1.1 ± 1.4 rotations (range 0–4) in self‐terminating VF. In Cryo EHTs, 5 of 62 rotor events, each from a different EHT, reached ≥7 rotations and 3 reached ≥17 rotations, with the three most persistent events accumulating 23, 25, and 21 rotations. In contrast, none of the 111 paced aLQTS rotor events reached 7 rotations, despite three persisting for ≥1.4 s. Thus, although differences in cycle length and mapping methodology preclude direct classification, Cryo EHTs generated a subset of rotors with stability comparable in rotation count to sustained human VF, whereas aLQTS rotors were predominantly short‐lived.

### 3.5 Limitations and Future Directions

Several limitations of the present work should be considered. The 3:1 cardiomyocyte‐to‐cardiac‐fibroblast ratio used here is empirically optimized in the parent milliPillar platform [15, 17, 23] and independently validated by Tiburcy et al.’s engineered human myocardium platform [24]. Future studies with different fibroblast composition, cell types, geometry, or post‐injury remodeled tissue could guide further optimization. Although the platform reproduces several tissue‐level electrophysiology parameters within adult mammalian range, the tissues are electrophysiologically intermediate rather than uniformly immature—action potential duration at 1 Hz lies within the adult human range while longitudinal conduction velocity remains approximately half of it; and we did not define the full maturation trajectory or perform longitudinal ultrastructural comparisons with human myocardium. The qPCR panel documents directional transcriptional maturation between day 7 and day 23, but it was not benchmarked against adult human ventricular tissue and therefore cannot establish the absolute maturation state. The goal of the study was not to titrate maturation conditions or timepoints but to demonstrate the tissue‐scale arrhythmogenic phenotype.

Donor genetics represents a further axis of variability that we did not cover; although the two hiPSC lines tested here differed in aLQTS susceptibility while showing comparable reentry responses to focal cryoinjury. Future studies can readily incorporate additional donor lines and extend the workflow to patient‐specific hiPSC lines carrying LQTS or cardiomyopathy variants to probe how inherited electrophysiological vulnerability shapes the tissue‐scale arrhythmia phenotype.

Both experimental models have defined scope: the aLQTS model may not generalize to other LQTS variants or VT etiologies, and focal cryoinjury reproduces the acute peri‐lesional electrophysiology of cardiomyocyte loss without the ischemia‐reperfusion cycle, inflammatory infiltrate, or chronic fibrotic remodeling characteristic of post‐infarct scar. Optical mapping provides a top‐surface‐weighted readout, and events below the imaging field cannot be captured.

Conservative PS/rotor detection criteria may underestimate event durations. Recording windows (total of 1–2 min divided into 10–20 s recordings) are set by voltage‐dye photobleaching kinetics and limit long‐term evaluation of transient arrhythmia events that self‐terminate within the window, whereas several cryoinjury rotors persist across the entire window and are reported as recording‐limited lower bounds.

Additionally, fluorescent dyes and blebbistatin electromechanical uncoupling can also influence electrophysiology; therefore, orthogonal validation using animal models or donor human heart sections would strengthen future observations. Finally, high‐cost mapping systems limit accessibility, though open‐source CMOS‐based alternatives [63, 64, 65] partially mitigate this barrier.

### 3.6 Concluding Remarks

In summary, hiPSC‐derived EHTs coupled with high‐resolution optical mapping reproduce baseline electrophysiological parameters within the range of the adult human ventricle for action potential duration, with conduction velocities approximately half of adult human longitudinal values and –under directed perturbations – the tissue‐scale mechanisms of both trigger‐ and substrate‐driven ventricular arrhythmias. The aLQTS condition generates EAD‐driven triggered activity and short‐lived functional rotors confined to head/neck geometric heterogeneities, whereas focal cryoinjury anchors prolonged rotors at the injury border. Together, these observations position EHTs as scalable, non‐animal platforms for mechanistic arrhythmia research and preclinical antiarrhythmic screening across trigger‐ and substrate‐driven arrhythmia classes, with a path toward gene‐environment extensions via patient‐specific hiPSC lines and more physiologic ischemic substrates.

## 4 Methods

### 4.1 Cell Sources and Culture

Human iPSC‐ventricular cardiomyocytes (hiPSC‐CMs) were derived from the hPSCreg line #TMOi001‐A (Gibco Episomal iPSC Line; A18945, iPSC line 1), differentiated and expanded according to the massive cardiomyocyte expansion protocol [66]. Only batches that formed confluent, spontaneously beating monolayers after four days of metabolic selection were used for further expansion. Cells underwent two passages in the presence of 2 µM CHIR‐99021 (Selleckchem S2924) for up to 35 days, followed by a one‐week recovery period without CHIR‐99021. Our cardiomyocyte population was ∼95% pure. We used Axol hiPSC‐CMs (Axol, ax2508) as iPSC line 2 and expanded them as described above. We obtained primary human ventricular fibroblasts (Lonza NHCF‐V), expanded them according to the manufacturer's protocol, and used them up to passage 5.

### 4.2 Silicone‐form Generation

milliPillar designs [17] were 3D‐printed with temperature‐resistant resin (Phrozen Sonic Mini 8K, TR300 Ultra‐High Temp 3D Printing Resin). Several modifications were introduced to the computer numerical control (CNC) milliPillar molds [17] to improve printing accuracy: (i) all modules were interconnected to stabilize the individual milliPillar units; (ii) the mold faces surrounding each milliPillar were raised by 200 µm, preserving pillar geometry while allowing flat sanding of both faces with 2000‐grit sandpaper; (iii) registration pins were replaced with M2×6 mm screws, (iv) the top pocket microchannel was eliminated from the top mold and replaced by a fence to facilitate handling of individual milliPillar modules. To prevent cure inhibition, printed molds were treated with 405‐nm UV light for 1 h while rotating continuously using a 3D printing curing station, followed by heat treatment at 100°C for 3 h and then at 65°C overnight. After two casting cycles, cure inhibition was no longer observed.

Ease Release 200 (Mann Release Technologies) was sparsely sprayed on mold faces to facilitate mold release. PDMS (base‐to‐curing‐agent 10:1, EMS 24236‐10) was degassed, poured into the printed molds, degassed again, then clamped on both faces and cured for 24 h at 65°C as described [17]. After unmolding, molds were cured for an additional 24 h, washed with MilliQ water, 70% ethanol, and MilliQ water, then dried at 65°C. The milliPillar modules were trimmed, tapped onto a very thin PDMS layer, and glued into 6‐well plates, then cured overnight at 45°C.

Each glued milliPillar module was plasma‐treated using a handheld corona plasma treater (Electro‐Technic Products BD‐20AC) equipped with a spring‐tip electrode to deliver corona plasma to the glued milliPillar module. A total of 15 s at low‐to‐medium dial intensity was sufficient to increase PDMS hydrophilicity. The plate was subsequently sterilized with 70% ethanol for 30 min, air‐dried, and UV‐C irradiated (UV Clave, Benchmark Scientific) for 20 min directly under UV bulbs (600 mJ/cm^2^) inside a biosafety cabinet. UV‐C indicator cards were used to monitor UV‐C exposure (B1450‐IC, Benchmark Scientific). After sterilization, milliPillar modules were blocked with KOSR (Gibco 10828‐028) for 1 h at 37°C.

### 4.3 EHT Plating

Cells were detached with TrypLE Select (Gibco 12563011), centrifuged (300 xg, 3 min), counted, and resuspended in plating medium RPMI (Gibco 11875093) supplemented with B27 (Gibco 17504044), 10% KOSR, 1X penicillin/streptomycin (Gibco 15140122), and 5 µM Y‐27632 (Selleckchem S1049). We plated EHTs in batches of at least 12, including at least 3 additional tissues to account for pipetting losses. Each EHT contained 550,000 viable cells at a 3:1 cardiomyocyte‐to‐fibroblast ratio. Cells were pelleted and resuspended in 15 µL of matrix solution per EHT, consisting of 12 µL of PureCol EZ Gel (BICO 5074, 5 mg/mL bovine atelocollagen) and 3 µL plain RPMI supplemented with 25 µM Y‐27632, to achieve a final collagen concentration of 4 mg/mL and 5 µM Y‐27632.

EHT suspension (15 µL) was homogeneously dispersed without introducing bubbles to each milliPillar module pocket near the pillar heads by sweeping the pipette tip across the pocket while dispensing to the opposite pillar. We spread the mixture around the upper fence to make sure the mixture remains around pillar heads by capillarity. Here we identified several problems that affected our plating: (i) excess PDMS used to glue inserts was detached by the pipetting motion during plating, incorporating PDMS into the EHT matrix; (ii) leakage between PDMS and the 6‐well plate bottom. These problems were solved, as described above, by spreading a very thin layer of PDMS on a flat surface of a 6‐well plate and then tapping the milliPillar onto the 6‐well plates. By looking under the base of the plate, we were able to determine that a continuous layer of PDMS was around the milliPillar module, without leaking into the pocket chamber; (iii) bubble incorporation, causing EHTs structural defects. Here, it is important to gently pipet the suspension without introducing bubbles. Plasma treatment significantly reduced bubble generation from air entrapment.

After plating, ∼25 µL plating medium was added along the well wall to prevent dehydration before incubation for 1 h at 37°C. Plating medium was added afterward, on the sides of the wall without disturbing the EHT mixture. On the following day, culture media was replaced with maturation medium containing higher Ca^2+^, lipids, low glucose, and supplements to match RPMI composition as described [25]. Maturation media was based on DMEM low glucose with GlutaMAX Supplement and sodium pyruvate (DMEM, Gibco 10567014) containing 1X B27, 1X penicillin/streptomycin, 10% KOSR, 5 µg/mL cobalamin (Thermo Scientific Chemicals A1489403), 0.82 µM biotin (Thermo Scientific Chemicals A1420703), 1X non‐essential amino acids (Gibco 11140–50), and 1X Normocin (InvivoGen ant‐nr‐2). Media were refreshed every 2–3 days. From day 7 to 21, 100 nM triiodothyronine (Millipore Sigma T2877) and 1 µM dexamethasone (Cell Signaling Technologies 14776S) were added [26].

### 4.4 Immunofluorescence

EHTs were fixed with methanol at −20°C for 20 min, followed by blocking and permeabilization in a solution containing 10% normal goat serum (Southern Biotech 0060‐01) and 0.5% Triton X‐100 in 1X phosphate‐buffered saline (PBS) for 1 h. Primary antibodies were incubated for 2 h at 30°C. Mouse anti‐sarcomeric α‐actinin (Invitrogen MAI‐22863), and rabbit anti‐vimentin (Cell Signaling 5741S) both 1:200 dilution, or rabbit anti‐sarcomeric α‐actinin (Invitrogen 701914, 1:200) and mouse anti‐cardiac troponin T (Abcam ab8295, 1:100). Additional primary antibodies were rabbit anti‐connexin 43 (Cell Signaling, 3512S; 1:50), mouse anti‐troponin I (Santa Cruz, sc‐133117; 1:50), and rabbit anti‐MLC2v (ProteinTech, 10906‐1‐AP; 1:200).

Secondary antibodies anti‐mouse Alexa Fluor Plus‐488 (Invitrogen A32723), anti‐rabbit Alexa Fluor Plus‐555 (Invitrogen A32732), anti‐rabbit Alexa Fluor Plus‐488 (Invitrogen A32731), and anti‐mouse Alexa Fluor 633 (Invitrogen A21052) were used at a dilution of 1:500 for 2 h at 30°C. DAPI was used for nuclear staining. After staining, EHTs were treated with the TrueView autofluorescence quenching kit (Vector Laboratories SP‐8400‐15) according to manufacturer instructions and mounted using Vectashield Vibrance mounting media (Vector Laboratories H‐1700‐2). Imaging was performed using a Nikon AXR/NSPARC confocal microscope, with a 4X and 60X oil objective.

### 4.5 Real‐Time PCR

RNA was extracted using the Quick‐RNA microprep kit (Zymo Research). RNA concentration was measured, and 500 ng of RNA was converted to cDNA using iScript Reverse Transcription Supermix (Bio‐Rad). We pre‐amplified with SsoAdvanced PreAmp Supermix (Bio‐Rad) using a pooled primer mix to enrich gene targets. RNA levels were assessed by semiquantitative PCR using iTaq Universal SYBR Green Supermix (Bio‐Rad) with a CFX Connect (Bio‐Rad). We analyzed relative transcript levels using CFX Maestro. Cq values were efficiency‐corrected per amplicon (primer efficiencies 90.0–106.8%, mean 96.5%) and normalized to the geometric mean of the reference genes (GAPDH, TBP, 18S). Relative expression was calculated by the efficiency‐corrected ΔΔCt (Pfaffl) method. Primer sequences were previously described [67], and primer efficiencies were calculated for this study and are described in Table S4.

### 4.6 Contraction Assessment

We used C‐Pace and 6‐well carbon electrodes (IonOptix) to deliver field electrical stimulation at 1, 2, and 3 Hz using biphasic 12 V, 2 ms pulses. Brightfield recordings were obtained with an inverted microscope equipped with a 4X objective and an IDS U3‐3130 CP camera controlled with the IDS Python library. Acquisition was performed at 200 fps at 1, 2, and 3 Hz with 60 s recording for each step. We calibrated pixel size using a stage micrometer. Contractility was quantified with BeatProfiler [27].

### 4.7 Optical Mapping Perfusion‐stage Mount

The perfusion stage comprises a base, tissue holder, clamps, and a slider to deliver point electrical stimulation (Figure 2C). The base was printed in two parts, including a perfusion bracket to hold a ThermoClamp (AutoMate Scientific) that was epoxied to the bottom part.

The tissue holder was printed in three parts, including the detachable tissue holder and two perfusion mounts. Perfusion mounts were used to connect 16‐gauge blunt‐end needles that were bent and shaped to optimize perfusion. The outlet opening was finished with a rounded 45° bevel to ensure smooth vacuum aspiration. The two inserts were secured with M2 × 6 mm nuts glued to the detachable tissue holder. The detachable tissue holder has a shallow central divot to hold a single milliPillar module containing an EHT, while two clamps prevent flotation during perfusion. A temperature probe was glued next to the tissue divot to monitor local temperature throughout the experiment.

We used a ThermoClamp perfusion pencil (AutoMate Scientific) to warm the bathing solution to 37°C throughout imaging. To maintain temperature during recording, when perfusion stops to avoid motion artifacts, we connected a heater under the detachable tissue holder. We glued a flat piece of scrap metal in place and attached a heating element with a feedback temperature probe (AutoMate Scientific). We then encapsulated the inner chamber in black electronics epoxy (MG Chemicals, 832B). Both the pencil and stage heater were controlled with a dual ThermoClamp 3.2 heating system (AutoMate Scientific). A setpoint of 45°C for both channels was sufficient to maintain 37°C within the EHT. Throughout all experiments, the temperature of the encapsulated heater remained below 45°C, and no deformation of the detachable tissue holder was observed. Nevertheless, users should independently verify the thermal behavior of their own setup to ensure that no overheating occurs under their specific operating conditions.

The mount was designed in Tinkercad (Autodesk) and printed using an Ender 3 V2 Neo 3D connected to a filament dryer box containing black polyethylene terephthalate glycol (PETG) 1.75 mm filament (Creality). STL files were converted to G‐code using UltiMaker Cura and printed at a layer height of 0.12 mm, 100% infill for the tissue holder and clamps and 20% for the base, 240°C for the nozzle and 70°C for the base, at a speed of 50 mm/s.

### 4.8 Solutions and Dye Loading

Tyrode's solution (135 mM NaCl, 5 mM KCl, 1.8 mM CaCl_2_, 1 mM MgCl_2_, 5.6 mM glucose, and 10 mM HEPES, buffered to pH 7.4 with 10 M NaOH) was employed for rinsing, loading, and perfusion. Blebbistatin (5 µM, Selleckchem S7099) was added to suppress contractions during optical mapping. The dual‐dye loading cocktail contained 30 µM RH‐237 (Invitrogen S1109), 10 µM Rhod‐2 AM (Invitrogen R1244), and 0.1 % Pluronic F127 (Invitrogen P6867). For pharmacological validation, 100 nM E‐4031 (MedChemExpress HY‐15551) was added to Tyrode's solution containing blebbistatin. All reagents, except E‐4031, were dissolved in dimethylsulfoxide (DMSO, Millipore Sigma D2650)

To model aLQTS, we used a low K^+^/Mg^2+^ modified Tyrode's solution (135 mM NaCl, 2 mM KCl, 1.8 mM CaCl_2_, 0.5 mM MgCl_2_, 5.6 mM glucose, and 10 mM HEPES, buffered to pH 7.4 with 10 M NaOH) plus 500 nM E‐4031. EHTs were individually loaded in 1 mL loading solution in a sealed microcentrifuge vial at 37°C for 20 min, rinsing with fresh Tyrode's Solution before and after loading.

### 4.9 Cryoinjury

We loaded EHTs with RH‐237 and Rhod‐2 AM and performed optical mapping before and after cryoinjury within 5 min. We achieved cryoinjury using a liquid nitrogen‐chilled 1 mm copper pipe. We flattened the tip to cover the surface between the lower‐left silicone edge and the center of the left EHT head. We positioned one edge of the frozen pipe on the silicone, then gently tilted it toward the EHT head until a freezing halo covering around 50% of the head was visible.

### 4.10 Optical Mapping Imaging Protocol

Loaded EHTs were positioned in the central divot, secured with EHT holders, and perfused at 37°C. Biphasic 2 ms pulses (4 V) were delivered via point electrodes overlapping one half of a pillar head. EHTs were paced from 1 to 3 Hz in 0.5‐Hz increments. For each frequency, perfusion continued for 45 s followed by a 15 s pause to minimize fluid‐induced motion. Dual‐channel voltage and Ca^2+^ images were acquired simultaneously at 1000 fps using a SciMedia MiCAM03 system with the lowest illumination intensity that provided adequate focus. After each recording, perfusion resumed, and tissue response was confirmed in SciMedia BV Workbench.

For imaging, a TTL generator programmed on an Arduino microcontroller triggered pacing for 1 min and during acquisition. We programmed a keypad to have protocols at different pacing frequencies for quicker experimentation. The microcontroller also controlled LED illumination and camera operation, while halting perfusion to avoid motion artifacts. An Arduino case was obtained from the Optogenetics and Neural Engineering (University of Colorado) website [68].

### 4.11 Statistical Analysis

We performed all statistical analyses on tissue‐level summary values (one value per tissue per condition), and we present data as boxplots with the center line denoting the median and the box spanning the interquartile range (25^th^‐75^th^ percentiles); sample sizes are reported in the figure legends. Normality was assessed using Shapiro‐Wilk. When normality assumptions were not met for any subpanel in a figure, we analyzed all subpanels in that figure using nonparametric tests to maintain a consistent statistical approach. We determined paired versus unpaired testing based on the data structure: experiments with complete within‐tissue measurements across levels were analyzed with paired methods, whereas datasets with incomplete pairing (e.g., heterogeneous frequency sets and exclusions for insufficient response) were treated as unpaired.

For paired experiments with >2 groups, differences were tested using the Friedman test, followed by paired Wilcoxon signed‐rank comparisons versus 1 Hz (Figure 1). Frequency‐response optical mapping data used the Kruskal‐Wallis test with Dunn's post‐hoc comparison vs 333 ms (Figure 2). In Figure S3, with normal distribution, differences were assessed using a paired two‐sided t‐test. In Figure 3, paired metrics were analyzed using a two‐sided paired Wilcoxon signed‐rank test because several paired differences deviated from normality. For Figure 4, some data did not meet normality assumptions, so we used a two‐sided unpaired Mann‐Whitney U test. These data were also treated as unpaired because recordings were not available at identical frequency sets for all tissues, as discussed in the results. A p < 0.05 was considered statistically significant.

### 4.12 Declaration of Generative AI and AI‐Assisted Technologies in the Writing Process

During the preparation of this work, the author(s) used OpenAI and Anthropic models to improve readability and grammar. After using this tool/service, the author(s) reviewed and edited the content as needed and took full responsibility for the content of the publication.

## Author Contributions

**Matthew Fiedler**: conceptualization, investigation, writing – original draft, formal analysis, writing – review and editing, visualization, validation, methodology. **Alejandra Vasquez Limeta**: conceptualization, investigation, writing – original draft, writing – review and editing, formal analysis, visualization, validation, methodology. **Ernesto Reyes Sanchez**: investigation, writing – review and editing, formal analysis, methodology. **Leah Carter**: investigation, writing – review and editing, formal analysis, methodology. **Francisco Altamirano**: conceptualization, investigation, funding acquisition, writing – original draft, writing – review and editing, methodology, supervision, formal analysis, validation, visualization.

## Conflicts of Interest

The authors declare no conflicts of interest.

## Supporting information




**Supporting File 1**: advs78429‐sup‐0001‐SuppMat.pdf.

**Supporting File 2**: advs78429‐sup‐0002‐SuppMat.zip.

**Supporting File 3**: advs78429‐sup‐0003‐VideoS1.mp4.

**Supporting File 4**: advs78429‐sup‐0004‐VideoS2.mp4.

**Supporting File 5**: advs78429‐sup‐0005‐VideoS3.mp4.

**Supporting File 6**: advs78429‐sup‐0006‐VideoS4.mp4.

**Supporting File 7**: advs78429‐sup‐0007‐VideoS5.mp4.

**Supporting File 8**: advs78429‐sup‐0008‐VideoS6.mp4.

**Supporting File 9**: advs78429‐sup‐0009‐VideoS7.mp4.

**Supporting File 10**: advs78429‐sup‐0010‐VideoS8.mp4.

**Supporting File 11**: advs78429‐sup‐0011‐VideoS8a.mp4.

**Supporting File 12**: advs78429‐sup‐0012‐VideoS8b.mp4.

**Supporting File 13**: advs78429‐sup‐0013‐VideoS8c.mp4.

**Supporting File 14**: advs78429‐sup‐0014‐VideoS9a.mp4.

**Supporting File 15**: advs78429‐sup‐0015‐VideoS9b.mp4.

## Data Availability

The data that support the findings of this study are available from the corresponding author upon reasonable request.

## References

[1] S. Omara , T. N. Jensen , L. Koeber , et al., “Ventricular Tachycardia Predicts all‐Cause Mortality and Nonsudden Cardiac Death in Nonischemic Cardiomyopathy,” JACC: Advances 4, no. 9 (2025): 102063, 10.1016/j.jacadv.2025.102063. PMC12362670 40811878

[2] L. Fabritz , S. N. Hatem , and S. Sossalla , “Arrhythmia‐induced Cardiomyopathy: Focus on Atrial Fibrillation,” Nature Reviews Cardiology 23 (2025): 197–207, 10.1038/s41569-025-01195-2. 40954334

[3] W. Zhu , X. Bian , and J. Lv , “From Genes to Clinical Management: A Comprehensive Review of Long QT Syndrome Pathogenesis and Treatment,” Heart Rhythm O2 5, no. 8 (2024): 573–586, 10.1016/j.hroo.2024.07.006. PMC11385408 39263612

[4] Z. Qu and J. N. Weiss , “Mechanisms of Ventricular Arrhythmias: From Molecular Fluctuations to Electrical Turbulence,” Annual Review of Physiology 77, no. 1 (2015): 29–55, 10.1146/annurev-physiol-021014-071622. PMC4342983 25340965

[5] B. B. Lerman , S. M. Markowitz , J. W. Cheung , G. Thomas , and J. E. Ip , “Ventricular Tachycardia Due to Triggered Activity,” JACC: Clinical Electrophysiology 10, no. 2 (2024): 379–401, 10.1016/j.jacep.2023.10.033. 38127010

[6] J. N. Weiss , A. Garfinkel , H. S. Karagueuzian , P. S. Chen , and Z. Qu , “Early Afterdepolarizations and Cardiac Arrhythmias,” Heart Rhythm 7, no. 12 (2010): 1891–1899, 10.1016/j.hrthm.2010.09.017. PMC3005298 20868774

[7] O. Baines , R. Sha , M. Kalla , et al., “Optical Mapping and Optogenetics in Cardiac Electrophysiology Research and Therapy: A State‐of‐the‐Art Review,” Europace 26, no. 2 (2024): euae017, 10.1093/europace/euae017. PMC10847904 38227822

[8] D. J. Blackwell , J. Schmeckpeper , and B. C. Knollmann , “Animal Models to Study Cardiac Arrhythmias,” Circulation Research 130, no. 12 (2022): 1926–1964, 10.1161/CIRCRESAHA.122.320258. PMC9202503 35679367

[9] S. M. Ahmed , R. V. Shivnaraine , and J. C. Wu , “FDA Modernization Act 2.0 Paves the Way to Computational Biology and Clinical Trials in a Dish,” Circulation 148, no. 4 (2023): 309–311, 10.1161/CIRCULATIONAHA.123.065585. PMC10902210 37486997

[10] M. B. Josvai , C. L. Anderson , and L. L. Eckhardt , “The Role of iPSC Research for Insight Into Inherited Arrhythmia Conditions,” Heart Rhythm 22, no. 11 (2025): 2983–2994, 10.1016/j.hrthm.2025.03.2005. PMC12554002 40456302

[11] L. Reilly , M. Josvai , M. Kalluri , et al., “Modeling Idiopathic Ventricular Fibrillation Using iPSC Cardiomyocytes and Computational Approaches,” JACC: Clinical Electrophysiology 10, no. 12 (2024): 2628–2632, 10.1016/j.jacep.2024.07.014. PMC11671283 39297840

[12] E. Karbassi , A. Fenix , S. Marchiano , et al., “Cardiomyocyte Maturation: Advances in Knowledge and Implications for Regenerative Medicine,” Nature Reviews Cardiology 17, no. 6 (2020): 341–359, 10.1038/s41569-019-0331-x. PMC7239749 32015528

[13] X. Wu , K. Swanson , Z. Yildirim , W. Liu , R. Liao , and J. C. Wu , “Clinical Trials in‐a‐dish for Cardiovascular Medicine,” European Heart Journal 45, no. 40 (2024): 4275–4290, 10.1093/eurheartj/ehae519. PMC11491156 39270727

[14] I. Goldfracht , S. Protze , A. Shiti , et al., “Generating Ring‐Shaped Engineered Heart Tissues From Ventricular and Atrial Human Pluripotent Stem Cell‐Derived Cardiomyocytes,” Nature Communications 11, no. 1 (2020): 75, 10.1038/s41467-019-13868-x. PMC6946709 31911598

[15] K. Ronaldson‐Bouchard , S. P. Ma , K. Yeager , et al., “Advanced Maturation of human Cardiac Tissue Grown From Pluripotent Stem Cells,” Nature 556, no. 7700 (2018): 239–243, 10.1038/s41586-018-0016-3. PMC5895513 29618819

[16] C. Tu , A. Caudal , Y. Liu , S. M. Narayan , and J. C. Wu , “Modeling Heart Rhythm Using Human Engineered Heart Tissues,” Nature protocols 21 (2025): 827–850, 10.1038/s41596-025-01217-w. PMC13132113 40750715

[17] M. A. Tamargo , T. R. Nash , S. Fleischer , et al., “milliPillar: A Platform for the Generation and Real‐Time Assessment of Human Engineered Cardiac Tissues,” ACS Biomaterials Science & Engineering 7, no. 11 (2021): 5215–5229, 10.1021/acsbiomaterials.1c01006. PMC9233181 34668692

[18] C. Alexander , M. J. Bishop , R. J. Gilchrist , F. L. Burton , G. L. Smith , and R. C. Myles , “Initiation of Ventricular Arrhythmia in the Acquired Long QT Syndrome,” Cardiovascular Research 119, no. 2 (2023): 465–476, 10.1093/cvr/cvac103. PMC10064840 35727943

[19] M. Maruyama , S. F. Lin , Y. Xie , et al., “Genesis of Phase 3 Early Afterdepolarizations and Triggered Activity in Acquired Long‐QT Syndrome,” Circulation: Arrhythmia and Electrophysiology 4, no. 1 (2011): 103–111, 10.1161/CIRCEP.110.959064. PMC3045276 21078812

[20] J. Brugada , L. Boersma , C. J. Kirchhof , V. V. Heynen , and M. A. Allessie , “Reentrant Excitation Around a Fixed Obstacle in Uniform Anisotropic Ventricular Myocardium,” Circulation 84, no. 3 (1991): 1296–1306, 10.1161/01.cir.84.3.1296. 1715818

[21] B. Joung , Z. Xu , I. Kim , M. H. Lee , and S. Kim , “The Effect of Cryoinjury on Ventricular Tachycardia in the swine Right Ventricle,” Yonsei Medical Journal 47, no. 5 (2006): 672–679, 10.3349/ymj.2006.47.5.672. PMC2687753 17066511

[22] M. J. Reiter , Z. Zetelaki , C. J. Kirchhof , L. Boersma , and M. A. Allessie , “Interaction of Acute Ventricular Dilatation and D‐sotalol During Sustained Reentrant Ventricular Tachycardia Around a Fixed Obstacle,” Circulation 89, no. 1 (1994): 423–431, 10.1161/01.cir.89.1.423. 7506639

[23] S. Fleischer , T. R. Nash , M. A. Tamargo , et al., “An Engineered Human Cardiac Tissue Model Reveals Contributions of Systemic Lupus Erythematosus Autoantibodies to Myocardial Injury,” Nature Cardiovascular Research 3, no. 9 (2024): 1123–1139, 10.1038/s44161-024-00525-w. PMC11399098 39195859

[24] M. Tiburcy , J. E. Hudson , P. Balfanz , et al., “Defined Engineered Human Myocardium With Advanced Maturation for Applications in Heart Failure Modeling and Repair,” Circulation 135, no. 19 (2017): 1832–1847, 10.1161/CIRCULATIONAHA.116.024145. PMC5501412 28167635

[25] D. A. M. Feyen , W. L. McKeithan , A. A. N. Bruyneel , et al., “Metabolic Maturation Media Improve Physiological Function of Human iPSC‐Derived Cardiomyocytes,” Cell Reports 32, no. 3 (2020): 107925, 10.1016/j.celrep.2020.107925. PMC7437654 32697997

[26] L. Wang , Y. Wada , N. Ballan , et al., “Triiodothyronine and Dexamethasone Alter Potassium Channel Expression and Promote Electrophysiological Maturation of human‐induced Pluripotent Stem Cell‐derived Cardiomyocytes,” Journal of Molecular and Cellular Cardiology 161 (2021): 130–138, 10.1016/j.yjmcc.2021.08.005. PMC9809541 34400182

[27] Y. Kim , K. Wang , R. I. Lock , et al., “BeatProfiler: Multimodal in Vitro Analysis of Cardiac Function Enables Machine Learning Classification of Diseases and Drugs,” IEEE Open Journal of Engineering in Medicine and Biology 5 (2024): 238–249, 10.1109/OJEMB.2024.3377461. PMC11008807 38606403

[28] Q. Lou , V. V. Fedorov , A. V. Glukhov , N. Moazami , V. G. Fast , and I. R. Efimov , “Transmural Heterogeneity and Remodeling of Ventricular Excitation‐contraction Coupling in human Heart Failure,” Circulation 123, no. 17 (2011): 1881–1890, 10.1161/CIRCULATIONAHA.110.989707. PMC3100201 21502574

[29] M. Hayashi , W. Shimizu , and C. M. Albert , “The Spectrum of Epidemiology Underlying Sudden Cardiac Death,” Circulation Research 116, no. 12 (2015): 1887–1906, 10.1161/CIRCRESAHA.116.304521. PMC4929621 26044246

[30] B. R. Choi , F. Burton , and G. Salama , “Cytosolic Ca2+ Triggers Early Afterdepolarizations and Torsade de Pointes in Rabbit Hearts With Type 2 Long QT Syndrome,” The Journal of Physiology 543, no. 2 (2002): 615–631, 10.1113/jphysiol.2002.024570. PMC2290501 12205194

[31] J. N. Weiss , Z. Qu , P. S. Chen , et al., “The Dynamics of Cardiac Fibrillation,” Circulation 112, no. 8 (2005): 1232–1240, 10.1161/CIRCULATIONAHA.104.529545. 16116073

[32] K. K. Aras , N. R. Faye , B. Cathey , and I. R. Efimov , “Critical Volume of Human Myocardium Necessary to Maintain Ventricular Fibrillation,” Circulation: Arrhythmia and Electrophysiology 11, no. 11 (2018): 006692, 10.1161/CIRCEP.118.006692. PMC7722395 30376733

[33] B. J. Boukens , M. S. Sulkin , C. R. Gloschat , F. S. Ng , E. J. Vigmond , and I. R. Efimov , “Transmural APD Gradient Synchronizes Repolarization in the human Left Ventricular Wall,” Cardiovascular Research 108, no. 1 (2015): 188–196, 10.1093/cvr/cvv202. PMC4571834 26209251

[34] V. V. Fedorov , A. V. Glukhov , C. M. Ambrosi , et al., “Effects of KATP Channel Openers Diazoxide and Pinacidil in Coronary‐perfused Atria and Ventricles From Failing and Non‐failing human Hearts,” Journal of Molecular and Cellular Cardiology 51, no. 2 (2011): 215–225, 10.1016/j.yjmcc.2011.04.016. PMC3124600 21586291

[35] S. A. George , J. A. Brennan‐McLean , K. A. Trampel , et al., “Ryanodine Receptor Inhibition With Acute Dantrolene Treatment Reduces Arrhythmia Susceptibility in human Hearts,” American Journal of Physiology‐Heart and Circulatory Physiology 325, no. 4 (2023): H720–H728, 10.1152/ajpheart.00103.2023. PMC12871874 37566110

[36] A. V. Glukhov , V. V. Fedorov , Q. Lou , et al., “Transmural Dispersion of Repolarization in Failing and Nonfailing human Ventricle,” Circulation Research 106, no. 5 (2010): 981–991, 10.1161/CIRCRESAHA.109.204891. PMC2842469 20093630

[37] C. Kang , Y. Qiao , G. Li , et al., “Human Organotypic Cultured Cardiac Slices: New Platform for High Throughput Preclinical Human Trials,” Scientific Reports 6 (2016): 28798, 10.1038/srep28798. PMC4928074 27356882

[38] I. Koncz , A. O. Verkerk , M. Nicastro , et al., “Acetylcholine Reduces IKr and Prolongs Action Potentials in Human Ventricular Cardiomyocytes,” Biomedicines 10, no. 2 (2022): 244, 10.3390/biomedicines10020244. PMC8869322 35203454

[39] D. Lang , K. Holzem , C. Kang , et al., “Arrhythmogenic Remodeling of β 2 Versus β 1 Adrenergic Signaling in the Human Failing Heart,” Circulation: Arrhythmia and Electrophysiology 8, no. 2 (2015): 409–419, 10.1161/CIRCEP.114.002065. PMC4608687 25673629

[40] M. K. Madrid , K. A. Trampel , B. Salman , et al., “Selective RyR2 Inhibition Reduces Arrhythmia Susceptibility in Human Cardiac Slices,” The Journal of Physiology 604, no. 5 (2026): 1809–1819, 10.1113/JP290283. PMC12953022 41609498

[41] I. Y. Shadrin , B. W. Allen , Y. Qian , et al., “Cardiopatch Platform Enables Maturation and Scale‐up of Human Pluripotent Stem Cell‐derived Engineered Heart Tissues,” Nature Communications 8, no. 1 (2017): 1825, 10.1038/s41467-017-01946-x. PMC5705709 29184059

[42] A. V. Glukhov , V. V. Fedorov , P. W. Kalish , et al., “Conduction Remodeling in Human End‐Stage Nonischemic Left Ventricular Cardiomyopathy,” Circulation 125, no. 15 (2012): 1835–1847, 10.1161/CIRCULATIONAHA.111.047274. PMC3351089 22412072

[43] E. Querdel , M. Reinsch , L. Castro , et al., “Human Engineered Heart Tissue Patches Remuscularize the Injured Heart in a Dose‐Dependent Manner,” Circulation 143, no. 20 (2021): 1991–2006, 10.1161/CIRCULATIONAHA.120.047904. PMC8126500 33648345

[44] S. He , K. Kou , C. O'Shea , et al., “A Dataset of Dual Calcium and Voltage Optical Mapping in Healthy and Hypertrophied Murine Hearts,” Scientific Data 8, no. 1 (2021): 314, 10.1038/s41597-021-01085-5. PMC8677726 34916511

[45] Y. Li , Z. Liu , C. O'Shea , et al., “Dual Calcium‐Voltage Optical Mapping of Regional Voltage and Calcium Signals in Intact Murine RyR2‐R2474S Hearts,” Journal of Molecular and Cellular Cardiology Plus 10 (2024): 100121, 10.1016/j.jmccpl.2024.100121. PMC11649530 39697246

[46] T. Yang and D. M. Roden , “Extracellular Potassium Modulation of Drug Block of I Kr,” Circulation 93, no. 3 (1996): 407–411, 10.1161/01.cir.93.3.407. 8565156

[47] J. N. Weiss , Z. Qu , and K. Shivkumar , “Electrophysiology of Hypokalemia and Hyperkalemia,” Circulation: Arrhythmia and Electrophysiology 10 (2017): 3, 10.1161/CIRCEP.116.004667. PMC5399982 28314851

[48] M. Wang , M. Tashiro , and J. R. Berlin , “Regulation of L‐Type Calcium Current by Intracellular Magnesium in rat Cardiac Myocytes,” The Journal of Physiology 555, no. 2 (2004): 383–396, 10.1113/jphysiol.2003.048538. PMC1664853 14617671

[49] D. M. Roden , “Drug‐Induced Prolongation of the QT Interval,” New England Journal of Medicine 350, no. 10 (2004): 1013–1022, 10.1056/NEJMra032426. 14999113

[50] P. C. Chang , H. T. Wo , H. L. Lee , et al., “Role of Sarcoplasmic Reticulum Calcium in Development of Secondary Calcium Rise and Early Afterdepolarizations in long QT Syndrome Rabbit Model,” PLoS ONE 10 (2015): 0123868, 10.1371/journal.pone.0123868. PMC4395163 25875599

[51] K. Campbell , C. J. Calvo , S. Mironov , T. Herron , O. Berenfeld , and J. Jalife , “Spatial Gradients in Action Potential Duration Created by Regional Magnetofection of hERG Are a Substrate for Wavebreak and Turbulent Propagation in Cardiomyocyte Monolayers,” The Journal of Physiology 590, no. 24 (2012): 6363–6379, 10.1113/jphysiol.2012.238758. PMC3533198 23090949

[52] J. Stein , D. Greene , F. Fenton , and Y. Shiferaw , “Mechanism of Arrhythmogenesis Driven by Early after Depolarizations in Cardiac Tissue,” PLOS Computational Biology 21, no. 4 (2025): 1012635, 10.1371/journal.pcbi.1012635. PMC12047796 40261939

[53] M. Valderrabano , P. S. Chen , and S. F. Lin , “Spatial Distribution of Phase Singularities in Ventricular Fibrillation,” Circulation 108, no. 3 (2003): 354–359, 10.1161/01.CIR.0000080322.67408.B4. 12835210

[54] T. Ashihara , T. Namba , T. Ikeda , M. Ito , M. Kinoshita , and K. Nakazawa , “Breakthrough Waves During Ventricular Fibrillation Depend on the Degree of Rotational Anisotropy and the Boundary Conditions: A Simulation Study,” Journal of Cardiovascular Electrophysiology 12, no. 3 (2001): 312–322, 10.1046/j.1540-8167.2001.00312.x. 11291805

[55] Z. Qu , J. Kil , F. Xie , A. Garfinkel , and J. N. Weiss , “Scroll Wave Dynamics in a Three‐Dimensional Cardiac Tissue Model: Roles of Restitution, Thickness, and fiber Rotation,” Biophysical Journal 78, no. 6 (2000): 2761–2775, 10.1016/S0006-3495(00)76821-4. PMC1300866 10827961

[56] R. L. Chen , D. J. Penny , G. Greve , and M. J. Lab , “Stretch‐induced Regional Mechanoelectric Dispersion and Arrhythmia in the Right Ventricle of Anesthetized Lambs,” American Journal of Physiology‐Heart and Circulatory Physiology 286, no. 3 (2004): H1008–H1014, 10.1152/ajpheart.00724.2003. 14766676

[57] R. H. Keldermann , M. P. Nash , H. Gelderblom , V. Y. Wang , and A. V. Panfilov , “Electromechanical Wavebreak in a Model of the human Left Ventricle,” American Journal of Physiology‐Heart and Circulatory Physiology 299, no. 1 (2010): H134–H143, 10.1152/ajpheart.00862.2009. 20400690

[58] F. Xie , Z. Qu , J. Yang , A. Baher , J. N. Weiss , and A. Garfinkel , “A Simulation Study of the Effects of Cardiac Anatomy in Ventricular Fibrillation,” Journal of Clinical Investigation 113 (2004): 686–693, 10.1172/JCI17341. PMC351312 14991066

[59] Z. Qu , “Critical Mass Hypothesis Revisited: Role of Dynamical Wave Stability in Spontaneous Termination of Cardiac Fibrillation,” American Journal of Physiology‐Heart and Circulatory Physiology 290, no. 1 (2006): H255–H263, 10.1152/ajpheart.00668.2005. PMC3644506 16113075

[60] J. Chen , R. Mandapati , O. Berenfeld , A. C. Skanes , R. A. Gray , and J. Jalife , “Dynamics of Wavelets and Their Role in Atrial Fibrillation in the Isolated Sheep Heart,” Cardiovascular Research 48, no. 2 (2000): 220–232, 10.1016/s0008-6363(00)00177-2. 11054469

[61] L. Wetstein , R. Mark , A. Kaplan , H. Mitamura , C. Sauermelch , and E. L. Michelson , “Nonarrhythmogenicity of Therapeutic Cryothermic Lesions of the Myocardium,” Journal of Surgical Research 39, no. 6 (1985): 543–554, 10.1016/0022-4804(85)90123-4. 4068693

[62] D. E. Krummen , J. Hayase , D. J. Morris , et al., “Rotor Stability Separates Sustained Ventricular Fibrillation From Self‐Terminating Episodes in Humans,” Journal of the American College of Cardiology 63, no. 24 (2014): 2712–2721, 10.1016/j.jacc.2014.03.037. PMC4396824 24794115

[63] F. J. Alibhai , A. Masoumi , H. J. Kim , et al., “Protocol for Dual‐optical Mapping of Voltage and Calcium Sensors in Human Pluripotent Stem Cell‐Derived Cardiomyocytes,” STAR Protocols 6, no. 3 (2025): 104044, 10.1016/j.xpro.2025.104044. PMC12397942 40849912

[64] P. Lee , L. Hou , F. J. Alibhai , et al., “A Fully‐Automated Low‐Cost Cardiac Monolayer Optical Mapping Robot,” Frontiers in Cardiovascular Medicine 10 (2023): 1096884, 10.3389/fcvm.2023.1096884. PMC10240081 37283579

[65] D. Rybashlykov , J. Brennan , Z. Lin , I. R. Efimov , and R. Syunyaev , “Open‐Source Low‐Cost Cardiac Optical Mapping System,” PLoS ONE 17, no. 3 (2022): 0259174, 10.1371/journal.pone.0259174. PMC8970595 35358183

[66] R. G. C. Maas , S. Lee , M. Harakalova , et al., “Massive Expansion and Cryopreservation of Functional Human Induced Pluripotent Stem Cell‐Derived Cardiomyocytes,” STAR Protocols 2, no. 1 (2021): 100334, 10.1016/j.xpro.2021.100334. PMC7881265 33615277

[67] M. Deogharia , L. Venegas‐Zamora , A. Agrawal , et al., “Histone Demethylase KDM5 Regulates Cardiomyocyte Maturation by Promoting Fatty Acid Oxidation, Oxidative Phosphorylation, and Myofibrillar Organization,” Cardiovascular Research 120, no. 6 (2024): 630–643, 10.1093/cvr/cvae014. PMC11074792 38230606

[68] “The Optogenetics and Neural Engineering (ONE) Core at the University of Colorado School of Medicine,” 2017, https://optogeneticsandneuralengineeringcore.gitlab.io/ONECoreSite/projects/Arduino%20TTL%20Pulse%20Generator%20and%20Controller/).

