# Supplementary material for: Human Engineered Heart Tissues Reproduce Trigger‐ and Substrate‐Driven Mechanisms of Ventricular Tachycardia
Source: Adv Sci (Weinh). 2026 Oct 26:e78429. Online ahead of print. doi: 10.1002/advs.78429 (PMC13710728; doi:10.1002/advs.78429)
Supplement: Supplementary file 1 [file ADVS-9999-e78429-s013.pdf]

SUPPLEMENTARY INFORMATION

Human engineered heart tissues reproduce trigger- and substrate-driven mechanisms of ventricular tachycardia

Matthew Fiedler[1,2], Alejandra Vasquez Limeta[1], Ernesto Reyes Sanchez[1], Leah Carter[1], Francisco Altamirano[1,2,3,§]

[1]Department of Cardiovascular Sciences, Houston Methodist Research Institute, Houston Methodist Hospital, Houston, TX, USA
[2]Program in Physiology, Biophysics & Systems Biology, Weill Cornell Medicine, NY, NY, USA
[3]Department of Cardiothoracic Surgery, Weill Cornell Medicine, NY, NY, USA

Short title: Engineered heart tissue electrophysiology

[§]**Correspondence to:**

Francisco Altamirano, Ph.D. Department of Cardiovascular Sciences, Houston Methodist Research Institute. 6565 Fannin Street, Houston, TX 77030

Disclosures: The authors have no conflicts to disclose.

SUPPLEMENTARY METHODS

Optical mapping data analysis: We performed all data processing, analysis, and figure generation in Python using NumPy, SciPy, OpenCV, scikit-image, Pillow, pybaselines, and matplotlib.

Denoising, baseline correction, and normalization: Baseline electrophysiology was processed sequentially with Gaussian spatial denoising (σ = 2), third-order Butterworth temporal band-pass filtering (0.1-25 Hz), and a 3-frame temporal median filter within a binary tissue mask. Baseline drift was removed by Asymmetric Least Squares (ASLS, p = 0.01), with a λ scaled inversely with frequency ($5 \cdot 10^8$ at 1 Hz to $5 \cdot 10^7$ at 3 Hz). After subtraction, a third-order Savitzky-Golay filter smoothed the signal using an adaptive window (51-15 ms for 1-3 Hz). We normalized signals using a percentile-based approach (1st to 99th) to minimize the impact of pixel outliers.

For CTL, aLQTS and cryoinjury recordings, a more aggressive pipeline was used: gaussian spatial denoising (σ = 4), low-pass filtering (25 Hz, third-order Butterworth), a 5-frame temporal median filter, ASLS baseline correction (p = 0.01) with a fixed smoothness $\lambda \approx 6.4 \cdot 10^8$ (derived from a 1 Hz baseline cutoff at a 1000 Hz sampling frequency), and Savitzky-Golay smoothing (9 ms window, order 3). ASLS suppressed signal drift and diastolic-floor shifts driven by increasing beating frequency; 200 ms of ASLS padding was applied to avoid edge artifacts. For CTL and aLQTS recordings, we then normalized signals per pixel to the 1st-to-99th percentile range mapped to [0, 1], and saved the normalized stack for downstream analysis.

For Cryo recordings, we used a two-pass scheme. In the first pass, we divided each pixel trace by a single denominator: the 70th percentile of pixel amplitudes across the tissue and clipped to [0, 1]. This kept low-amplitude injured pixels near zero, so the lesion stood out clearly. From this stack we built an injury mask. For each recording, we defined two healthy tissue references: the 80th-percentile amplitude and the median SNR across all pixels. A pixel was labeled injured when both its amplitude fell below 20 % of the reference amplitude, and its SNR fell below 50 % of the reference SNR; i.e., pixels that were both much dimmer and much noisier than typical tissue. Because a large lesion drags both references down, we recomputed them using only the pixels labeled healthy in the first pass and re-applied the criteria. We cleaned the resulting mask by removing components smaller than 50 pixels, closing small gaps, filling internal holes, and intersecting it with the tissue outline. CTL recordings skipped injury detection and used the full tissue as healthy.

For each EHT, the voltage- and calcium-channel injury masks were combined by union, and the healthy region was defined as the tissue minus this union. In the second pass, we renormalized the ASLS-corrected, Savitzky-Golay-smoothed recording per pixel to its 1st-to-99th percentile range, mapped to [0, 1], within this healthy region (excluding the injured area), producing the normalized stack used for downstream rotor analysis.

Parameter calculations in baseline electrophysiology recordings: Four equally spaced 7 × 7 pixels ROIs were placed along the longitudinal axis of each EHT and used to measure APD_{80}, CaD_{80}, tau, and CV. Resulting trace for each ROI was smoothed (Savitzky-Golay window = 21 frames, order = 3). Peaks were detected on the smoothed trace using "SciPy find peaks" with a height >0.3X median intensity, prominence ≥ 0.2, and minimum inter-peak distance of 150 ms; peaks were further pruned by valley depth < 40 % of the preceding peak and a relative height ≥ 40 % of that peak, followed by a hard 200 ms binning that retained only the tallest peak per window. Beats were assigned from ROIs that aligned within ± 200 ms with propagation direction starting from point stimulation (right side, ROI1). For each accepted beat: peak time, dV/dt_{max}, and D80 (interval between 20% peak and 80% return to baseline) for voltage recordings with additional tau and R^2 from a mono-exponential fit to the 20-80% segment for Ca^{2+} recordings. Pairwise ROI1-4 were converted to cm using a pixel size of 0.0445 px/µm and used to calculate velocities based on

activation times calculated by dV/dt_{max} time between extreme ROIs. All beat-level metrics from each ROI per beat were filtered using an interquartile-range outlier filter (k=3) and summary statistics were calculated.

Ensemble activation and APD_{80} and DF maps for baseline electrophysiology recordings: Pixel-wise activation times were obtained from the first derivative peak of each beat after frame-wise Gaussian filtering ($\sigma = 1$). Outliers were removed via IQR, then maps were normalized to the earliest activation, upsampled 4X with cubic interpolation, and median-filtered (9 × 9) for display. Ensemble activation maps were averages of normalized beat-wise maps. APD_{80} maps used a uniform box blur (size = 9) followed by 3 × 3 median filtering; APD_{80} was defined as the interval between 20 % upstroke and 80 % repolarization per pixel, with IQR outlier removal and beat-wise averaging. Dominant frequency (DF) maps were generated after Gaussian spatial filtering ($\sigma = 2$) and Savitzky-Golay temporal smoothing (51 ms, order 3); DF was the frequency of maximal FFT amplitude within 0.2-6 Hz for each masked pixel. Ensemble DF statistics and cumulative spectra were exported as CSV; DF maps were median-filtered (9 × 9) only for visualization.

Measurements of AP-CaT latency in baseline electrophysiology recordings: KairoSight[1] was used to determine AP-CaT latency on the first beat of recordings at a 500 ms CL.

Parameter quantifications in Kymograph in CTL and aLQTS recordings: Median fluorescence along a 3-pixel line spanning the longitudinal axis generated voltage and Ca^{2+} fluorescence time-distance maps. Kymographs were displayed using a robust normalization (rolling 4 s) to mitigate drift. Raw traces were used for calculations after a 7 ms temporal gaussian temporal smoothing for APD and DI and 9 ms for dV/dt. Beat-wise activation times were defined by dV/dt_{max} per distance column. CV was derived from linear fits of activation time versus distance. APD_{80} used a 20% upstroke to 80% repolarization threshold, and diastolic interval (DI) was the interval from the prior-beat's 80% repolarization to the next upstroke onset.

Beat-resolved activation, APD, dV/dt maps and DF in CTL and aLQTS recordings: Voltage signals were smoothed (Savitzky-Golay, 9 ms window, order 3). Activation time per pixel was calculated from maximum dV/dt times. To ensure that activation, repolarization, and upstroke metrics were extracted from the same physiological event across the field of view, a ROI-guided wave-tracking procedure was applied. ROI-guided wave tracking selected a single propagating wave: activation candidates from ROI traces defined expected arrival times, and the activation of each pixel was taken as the local dV/dt maximum within ±120 ms (±140 ms for APD). Activation times were reported in milliseconds and referenced to the earliest activated pixel (time zero). APD_{80} was defined as the time between 20% upstroke and 80% repolarization. The dV/dt max map reported the upstroke velocity magnitude. Outliers were removed with IQR (k = 1.5) and gaps filled; spatial blurring ($\sigma = 1$) was applied for display. DF maps were computed per masked pixel using Welch PSD (Hann window, ≈2 s segments, 50% overlap) after Savitzky-Golay smoothing (7 ms). The dominant frequency was the peak within 0.5-20 Hz; post-processing used IQR (k = 1.5) and $\sigma = 1$ blurring. Maps were exported as overlays and per-pixel tables.

Phase singularities (PS) and wavelets in CTL, aLQTS and Cryo recordings: The image stack was smoothed with an edge-safe masked Gaussian filter ($\sigma = 2$) to avoid blurring across the tissue boundary. Each pixel trace was detrended against a 0.8 s running-mean baseline, mean-centered, and standardized by its SD; pixels below an adaptive variability threshold (SD < $\max(10^{-4}, 0.10 \times$ median tissue SD)) were excluded. Instantaneous phase was obtained from the temporal Hilbert transform. PS were detected per frame using a winding-number method on 2×2 pixel cells inside the valid mask: candidates with $|\Sigma\Delta\varphi| < 1.8\pi$ were rejected, and the remainder were assigned topological charge ±1 from the wrap direction. To reduce grid bias, detection was repeated on a half-pixel-shifted lattice reconstructed by bilinear interpolation of $\cos\varphi$ and $\sin\varphi$.

A single PS was placed at the centroid of each connected component (≥ 1 px), and detections within 5 px were merged.

We built PS tracks using greedy nearest-neighbor association that respects chirality, allowing ≤ 20 px inter-frame displacement, gaps up to 20 ms, a minimum lifetime of 200 ms, and ≥ 0.5 rotations measured by unwrapping the mean Hilbert phase on a 3-pixel ring around the tip. Per-track metrics were duration, cumulative path length, net displacement, and mean speed, with pixel distances converted to μm using the spatial calibration (0.0445 px/μm). Tissue-level statistics were pooled medians and maxima of these metrics, rotor incidence per minute, and peak concurrency from overlapping intervals. PS tracks were then reviewed by manual inspection of per-recording overlay videos, and tracks judged to be artifacts were excluded before cohort aggregation.

Anatomy normalization and cohort-level dwell time and centroid mapping: Each tissue mask was rotated to align its first principal axis with a shared long axis. We then applied a piecewise-linear longitudinal warp anchored on shoulder and end landmarks and a per-column vertical warp that equalized each tissue's per-column half-width to a cohort template to bring all tissues onto a common canonical canvas, followed by a left–right mirror-fold about the center that enforces bilateral symmetry and averages the two tissue ends. PS coordinates were transformed into this canonical frame (0.0445 px/μm). We computed two complementary maps per tissue. Bin-dwell maps accumulated, per frame, a residence time $\Delta t = 1/FPS$ (ms) at the canvas bin occupied by each PS observation, weighted by the inverse local Jacobian of the warp so that dwell values reflect native-tissue area; per-tissue histograms were then summed and normalized by per-pixel coverage of rotor-positive tissues to give mean dwell. Centroid-prevalence maps placed a single point at each track's centroid on the canonical canvas, dilated that point by a fixed radius, and reported the percent of tissues whose dilated centroid covered each bin. Both maps were rendered within a canonical EHT silhouette derived from the ensemble of aligned tissue masks, restricted to bins present in at least 10 % of tissues.

Wavelet (wavefront component) quantification and PS-wavelet overlays: Normalized voltage recordings were renormalized using the 2nd-98th percentiles of masked pixels, and Gaussian-smoothed ($\sigma = 4$) to remove outlier and further reduce noise. Instantaneous phase was obtained using temporal Hilbert transform. At each frame, a wavefront mask comprised pixels with phase distance to 0 ≤ 0.40 rad within the tissue mask and valid phase. Wavelets were defined as contiguous blobs of wavefront pixels, where pixels were considered connected if they touched by an edge or a corner (8-connectivity) with a minimum area of ≥ 25 px. To avoid over-fragmentation, only components whose area was at least 25% of the largest component in that frame were retained. Wavelet counts per frame were obtained from the filtered set. Wavelet tracks were built by greedy one-to-one overlap matching: predecessor-successor pairs with intersection/smaller-component area >0.20 were linked. Tracks shorter than 30 ms were discarded; the resulting persistence-filtered wavelet count time series was aligned with PS data for overlay plots and Suppl. videos.

SUPPLEMENTARY FIGURE LEGENDS

Supplementary Figure 1. Modified milliPillar platform for resin 3D printing. **A**. 3D rendering of the STL file model showing raft, supports and modified milliPillar. Front (**B**) and back (**C**) views of the printed mold produced with a Phrozen Sonic Mini 8K using temperature-resistant resin (TR300, Phrozen). **D.** Raft and supports were easily removed from the modified molds.

Supplementary Figure 2. Increased flexibility for the milliPillar platform using individual modules. A. A thin PDMS layer was spread on a 6-well plate lid with a L-shaped cell spreader. **B.** Individual milliPillar modules were trimmed, handled with forceps and placed onto the PDMS film for ~1 s; a subtle contrast change was visible initially at the module base indicating wetting without affecting pillar heads. Proper, incomplete and overfilled interfaces between the module bottom and the 6-well plate are shown (**C**-**D**). **E**. Upside-down view of the 6-well plate and corresponding microscope close-ups demonstrate reproducible attachment across wells. **F**. Using modules as individual units increases versatility, allowing plating from 100 mm dishes to multi-well plates; the subfigure shows complete self-assembly of 40 tissues at 24 h after corona plasma/KOSR treatment without the need of needle scoring. Approximately 79% of tissues maintained shape and remained contracting and viable for at least 3 months. Scale bar in microscope images represent 500 μm.

Supplementary Figure 3. Cardiomyocyte purity used for EHT casting. An aliquot of the amplified cardiomyocyte suspension used for EHT casting was replated in parallel from each of two independent, consecutive differentiation batches (Batch 1 and 2), fixed 24 h post-plating, and stained for (**A**) myosin light chain 2v (MLC2v, ventricular subtype), (**B**) cardiac troponin T (cTnT), and (**C**) sarcomeric α-actinin; nuclei were counterstained with DAPI. Wells were imaged on a BioTek Cytation 1 high-content imager at 10× as 12 × 12 stitched montages (144 fields per batch; total of 288 fields per marker; nuclei classified: 163,239 MLC2v, 175,387 cTnT, 168,900 α-actinin). Nuclei were segmented per tile with Cellpose 4.2.1 (CPSAM model, diameter = 15 px); each mask was expanded by 10 px and the mean marker-channel intensity of the expanded region was recorded and used to classify positive and negative cells for each marker. Each panel (A–C) shows, a representative field (DAPI in blue, staining in green; negative cells ringed yellow followed zoomed marker^{+}/marker^{-} example cells. A spatial heatmaps across the 12x12 grid for each batch is shown. The %-positive cells for Batch 1 / Batch 2 were: MLC2v = 96.6 % / 92.8 %; cTnT = 93.7 % / 97.2 %; α-actinin = 95.8 % / 99.3 %. Scale bars: 100 µm (representative field), 20 µm (zoom).

Supplementary Figure 4. qPCR expression of cardiac genes in EHTs at day 7 (d7, blue) vs day 23 (d23, red). Tissues were treated with T3/Dex from day 7 to day 21. RNA was extracted on days 7 and 23, and cDNA was converted with iScript. Targets were pre-amplified for 20 cycles with SsoAdvanced PreAmp Supermix (Bio-Rad) using a pooled primer mix. Reactions were run with iTaq Universal SYBR Green Supermix on a Bio-Rad CFX Connect; Cq values were efficiency-corrected per amplicon (primer efficiencies 90.0–106.8%, mean 96.5%) and normalized to the geometric mean of the reference genes (GAPDH, TBP, 18S). Relative expression was calculated by the efficiency-corrected ΔΔCt (Pfaffl) method and rescaled so that the d7 geometric mean = 1 for each gene. Boxes show median and IQR with whiskers to 1.5×IQR; dots are individual EHTs. Group means were compared with a two-sided Student's t-test when both groups passed Shapiro-Wilk normality ($\alpha = 0.05$, equal variance assumed) and with a two-sided Mann-Whitney U test otherwise; p-values were adjusted across all tested genes by the Benjamini-Hochberg FDR procedure. Each panel reports the test used, raw p, and adjusted q (*$q < 0.05$, **$q < 0.01$, ***$q < 0.001$; ns, not significant). N=5 per group.

Supplementary Figure 5: Confocal immunofluorescence of Cx43 and Troponin I in EHTs. Tissues were fixed with 4% PFA and stained with primary antibodies against Cx43 and Troponin I. Confocal images were obtained from the superficial layers of the EHT and are shown as

maximum Z-projection. Nuclei were counterstained with DAPI. Top and bottom panels show representative 60x confocal fields from highly organized and less organized regions, respectively. Background was subtracted using a rolling-ball algorithm, followed by median and gaussian filters to remove salt-and-pepper noise. Scale bars: 25 µm.

Supplementary Figure 6: E-4031 prolongs APD in EHTs. **A.** Ensemble APD_{80} maps for a representative EHT paced at 2 Hz recorded before (control, top) and after treatment with 100 nM E-4031 (bottom). E-4031 induced a homogenous APD_{80} prolongation throughout the tissue. **B.** Corresponding ensemble activation maps for the same tissue before (top) and after (bottom) E-4031 treatment; note the scale difference between panels. **C.** Normalized voltage traces at each ROI before (blue) and after (orange) E4031 treatment reveals prolonged APD_{80}. **D.** Quantification of APD_{80} before and after E-4031 treatment. Box plots display the median and IQR with overlaid scatter points representing each individual EHT. N = 5 independent EHTs (across 2 batches). Data were normally distributed, so a paired, two-sided t-test was used.

Supplementary Figure 7: Effect of combined electrolyte alteration and APD prolongation promotes VT-like arrhythmias in aLQTS
A. Representative AP and Ca^{2+} traces obtained in Tyrode (CTL), low K+/Mg2+ Tyrode and low K+/Mg2+ Tyrode + E-4031 (aLQTS). B. Combined electrolyte alteration and APD prolongation in aLQTS significantly increased time spent at heart rates >120 and >140 bpm (**B**) and time exhibiting phase singularities (PS, **C**). **D**. All six aLQTS EHTs (6/6) developed at least one PS, versus none (0/6) in CTL or lowK^+/Mg^{2+}. Box-and-whisker plots show median and interquartile range; dots are individual EHTs. Statistics: pairwise Mann–Whitney U (**B, C**) or Fisher's exact test (**D**), Bonferroni-corrected across the three pairwise comparisons; N = 6 EHTs per condition.

Supplementary Figure 8. EAD-driven triggered activity propagates head-to-head or locally in aLQTS EHTs. **A**. Voltage (blue) and Ca^{2+} (orange) traces from a representative EAD-positive aLQTS EHT highlighting phase-3 EADs (red dotted arrows) and the "normal" propagation immediately before and coinciding with the EAD (green dotted arrows where applicable). **B**. Schematic representation of propagation direction for the marked beats from panel A (left), with corresponding voltage activation maps (right) showing EAD-driven triggered activity and bifocal activation. No EADs were observed in CTL recordings.

Supplemental Figure 1

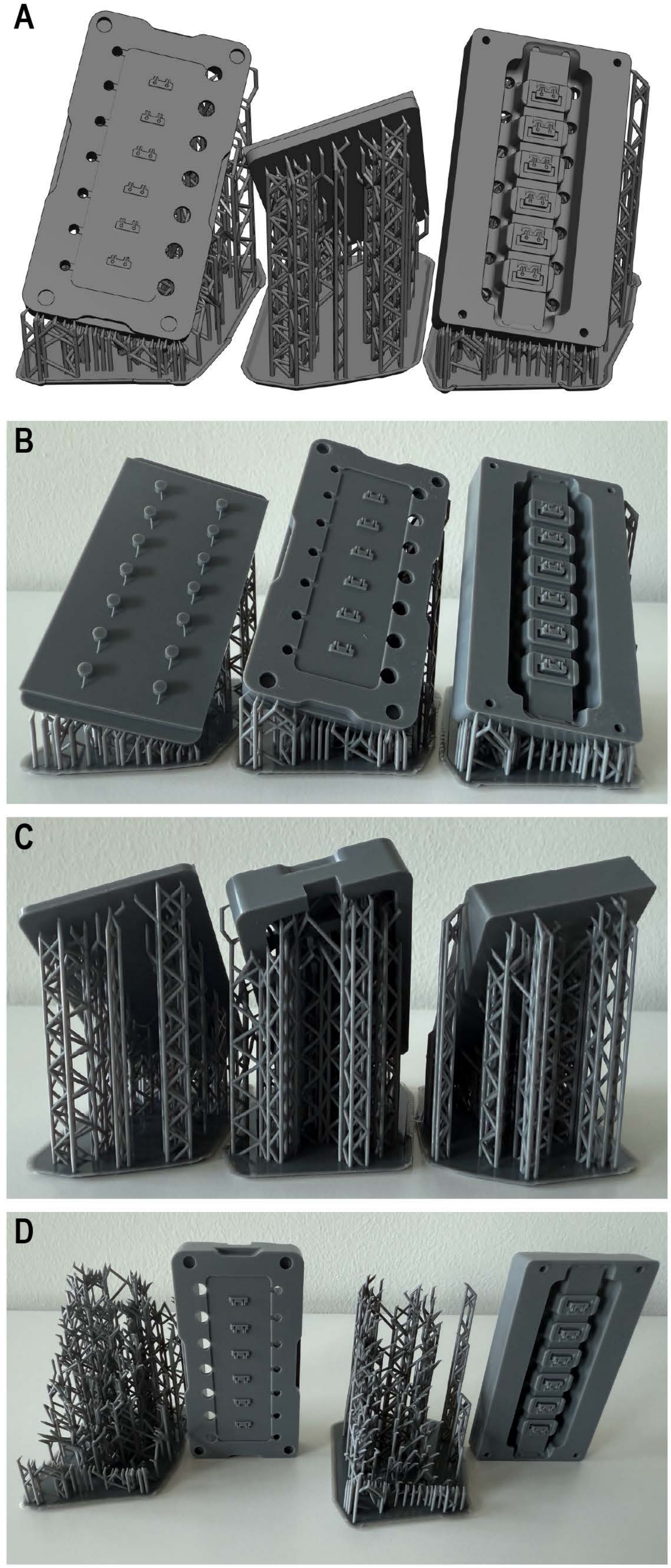

Supplemental Figure 2

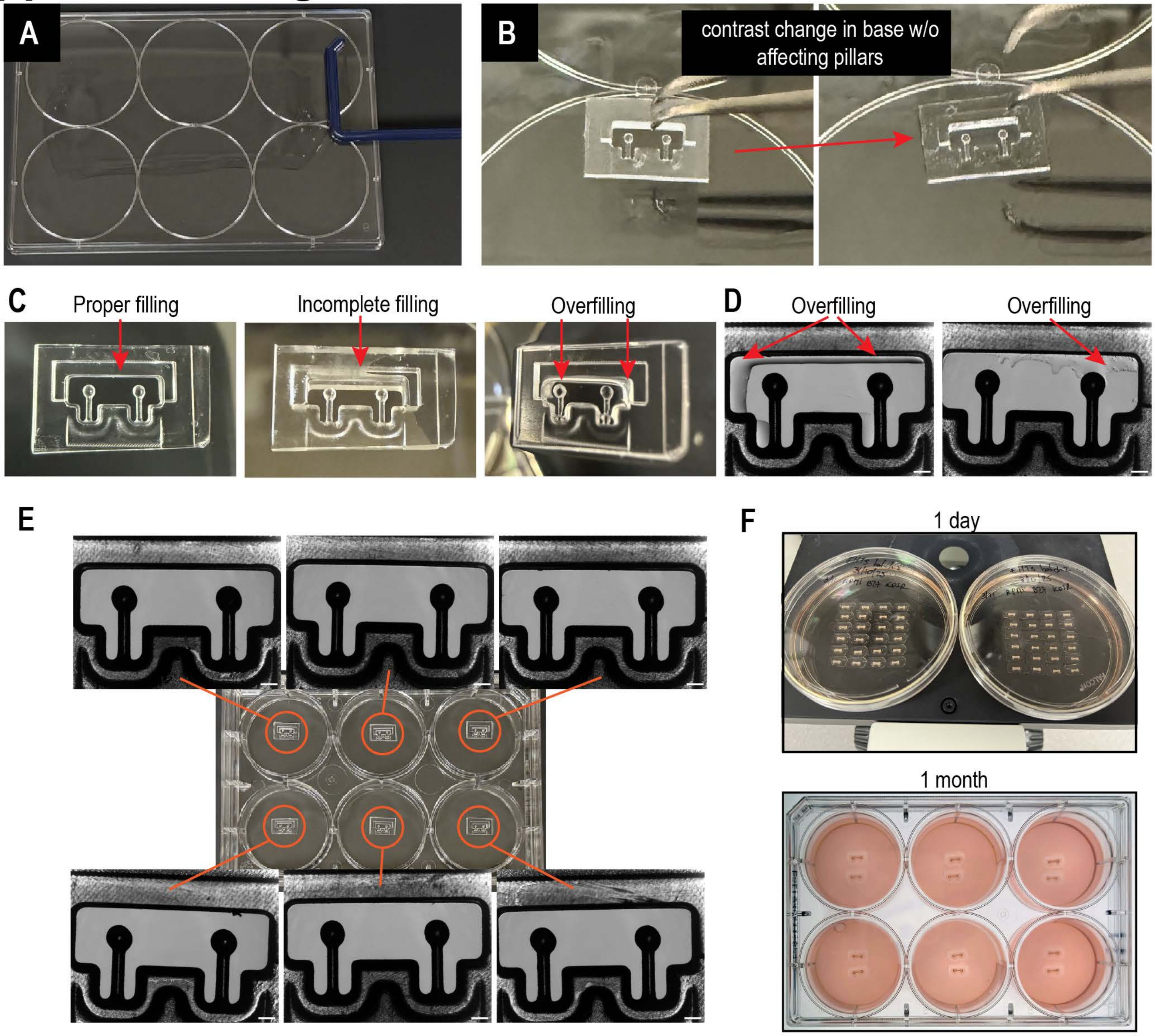

Supplemental Figure 3

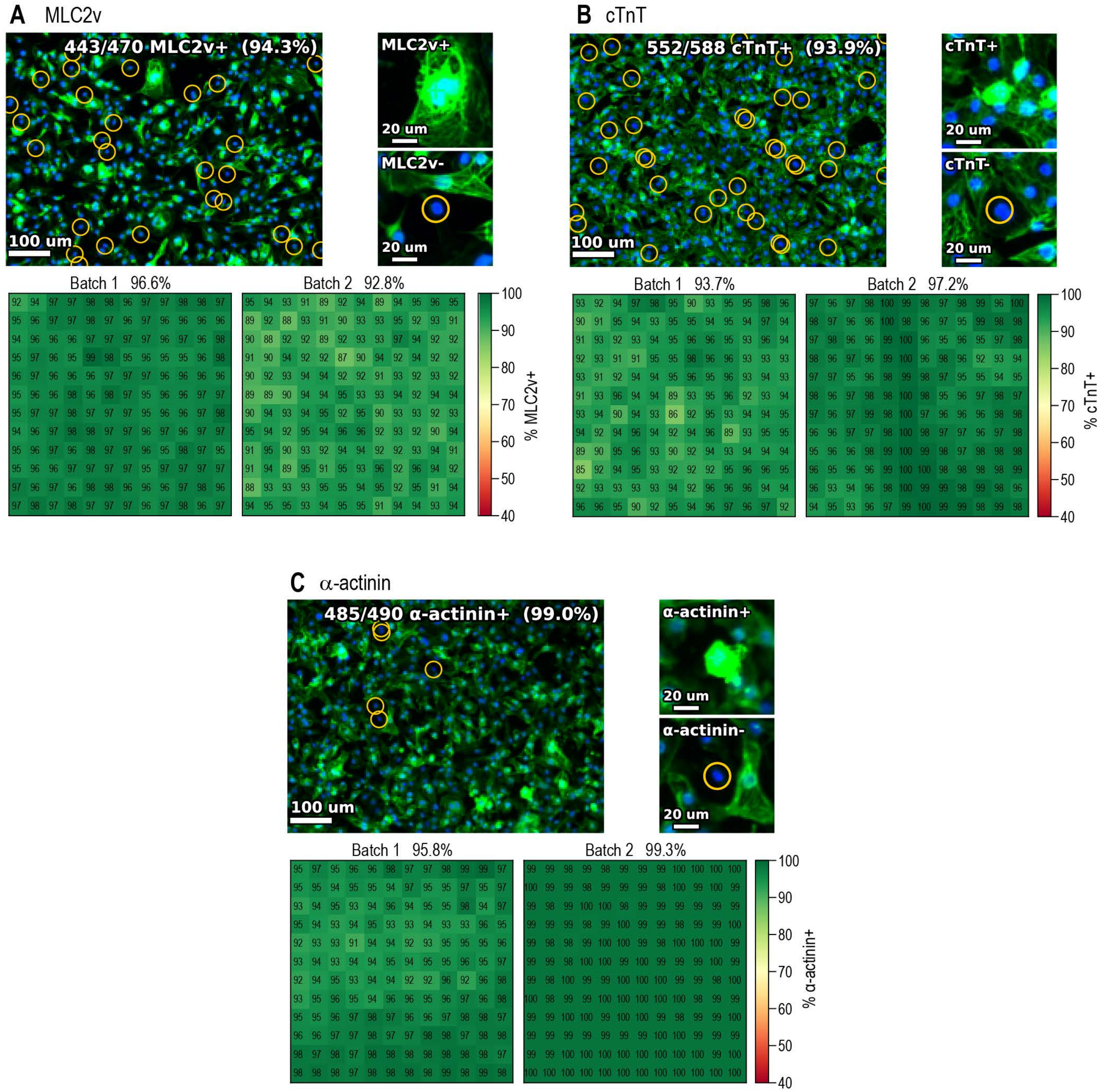

Supplemental Figure 4

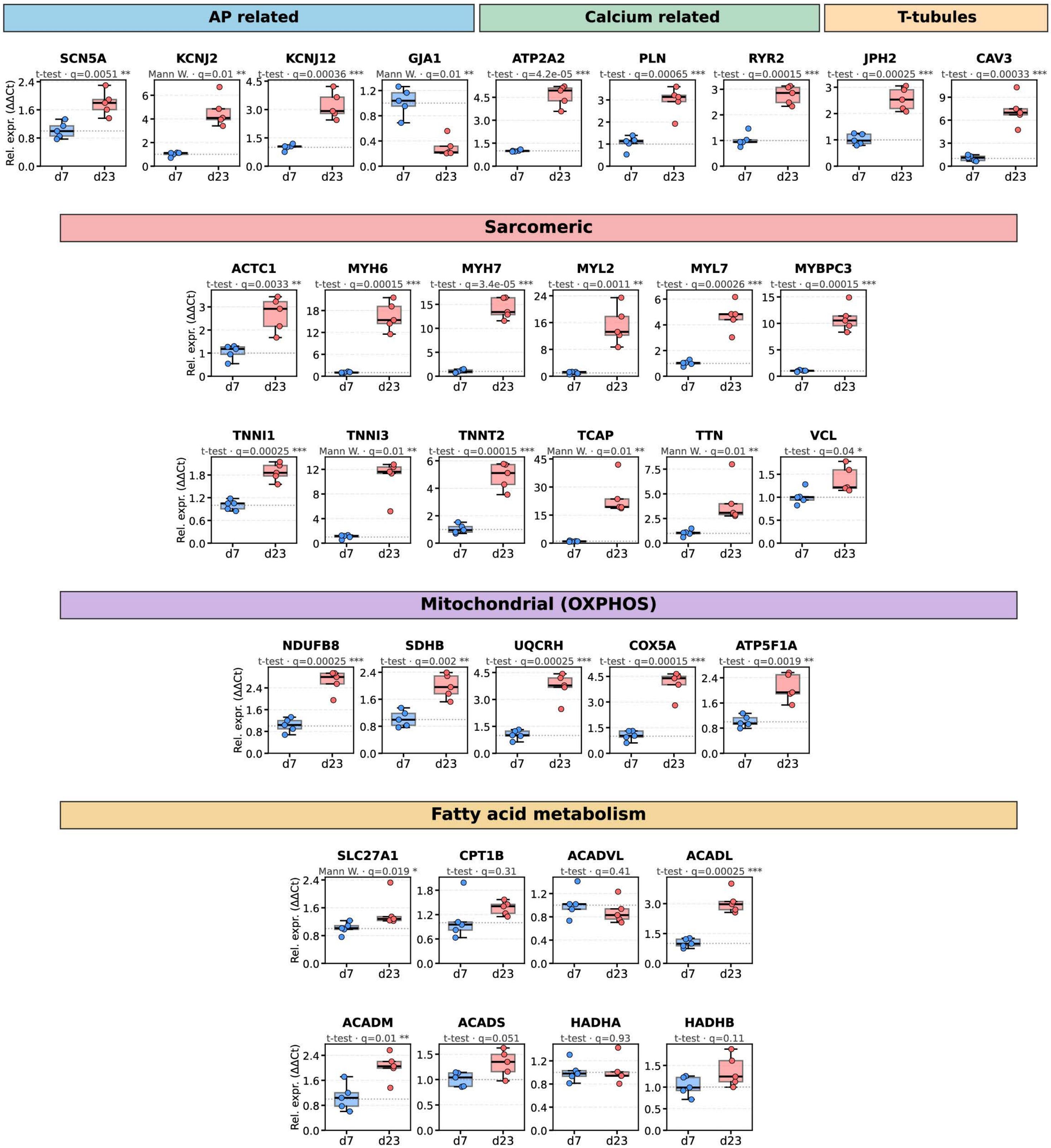

Supplemental Figure 5

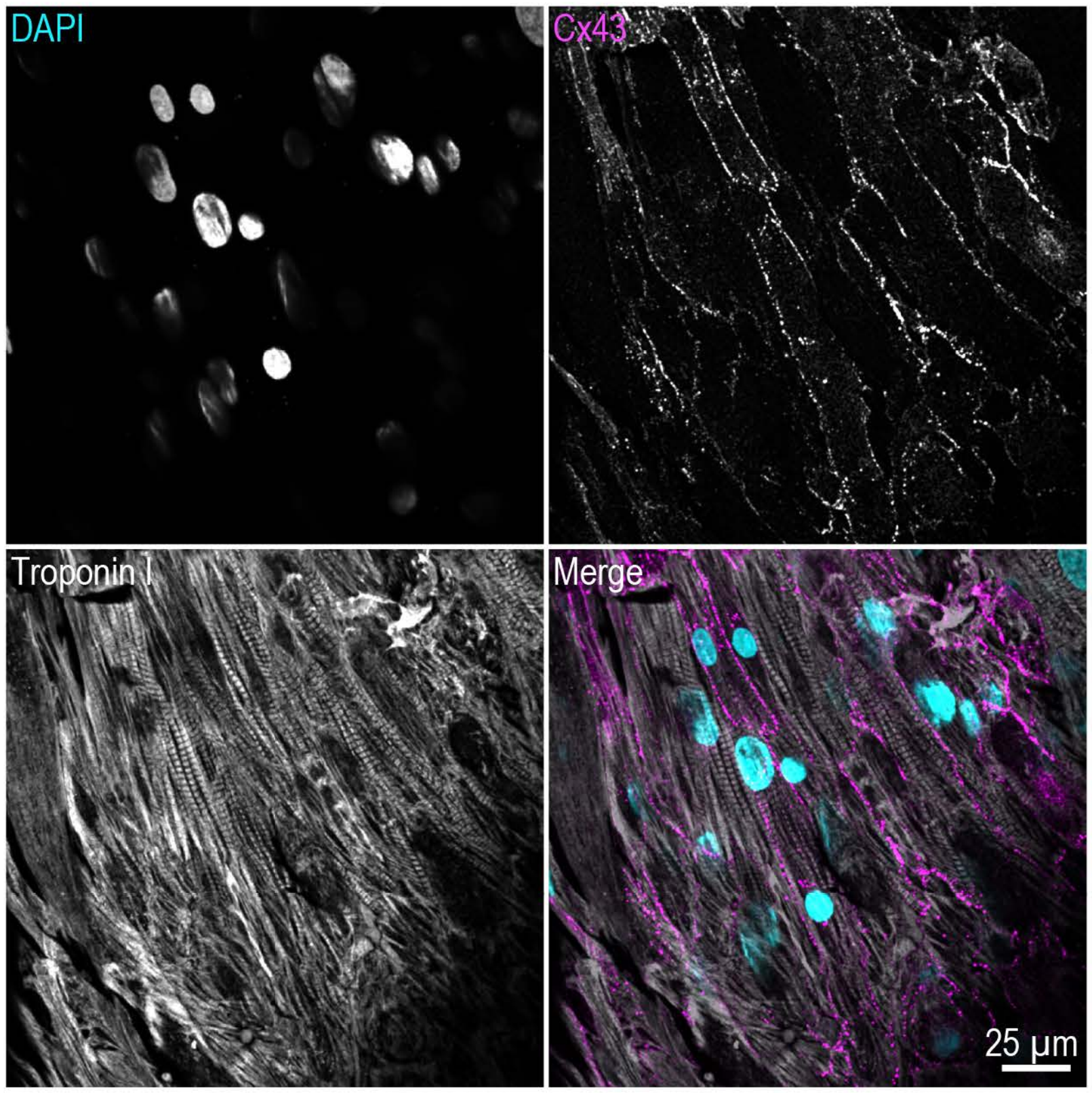

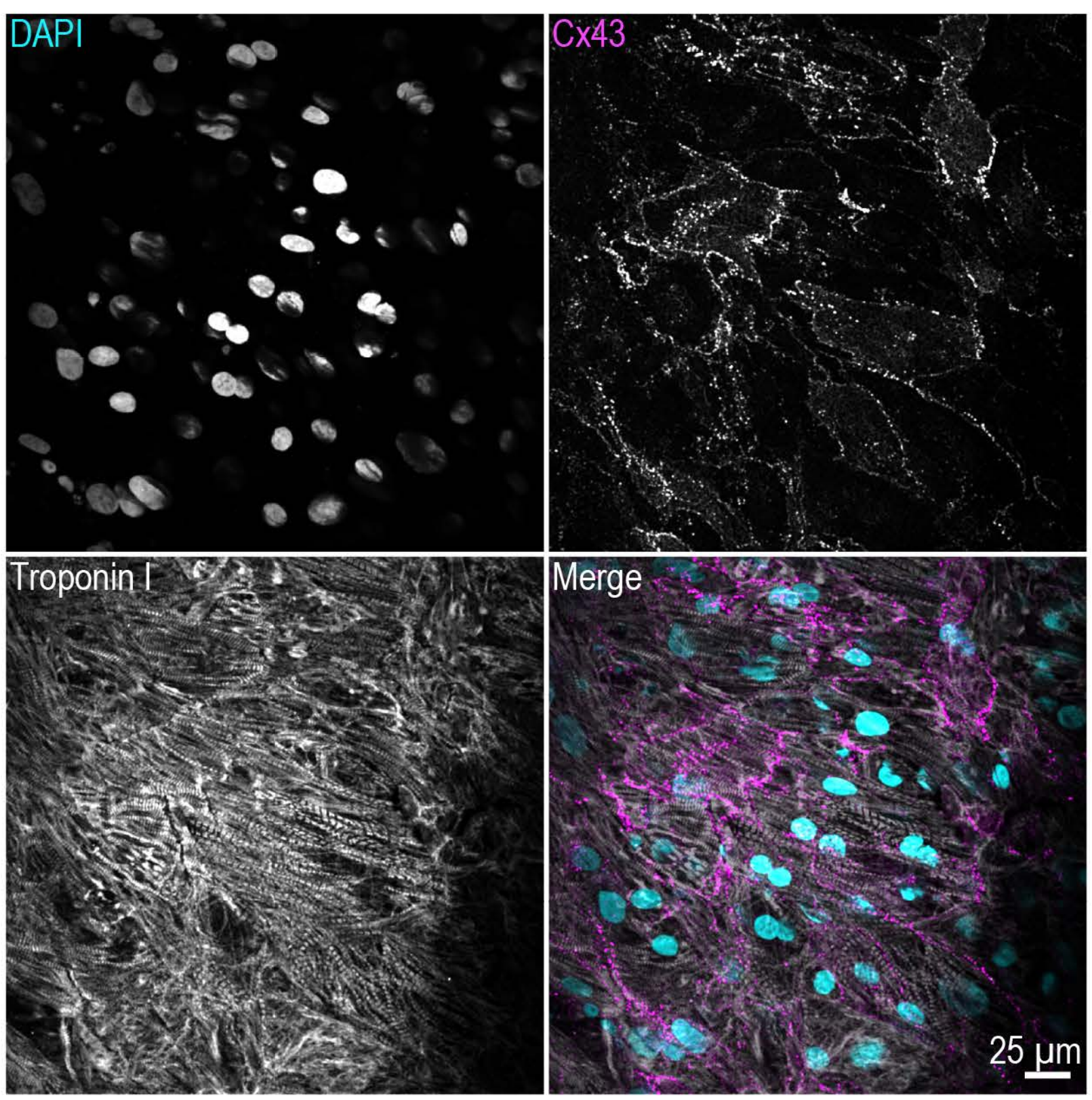

Supplemental Figure 6

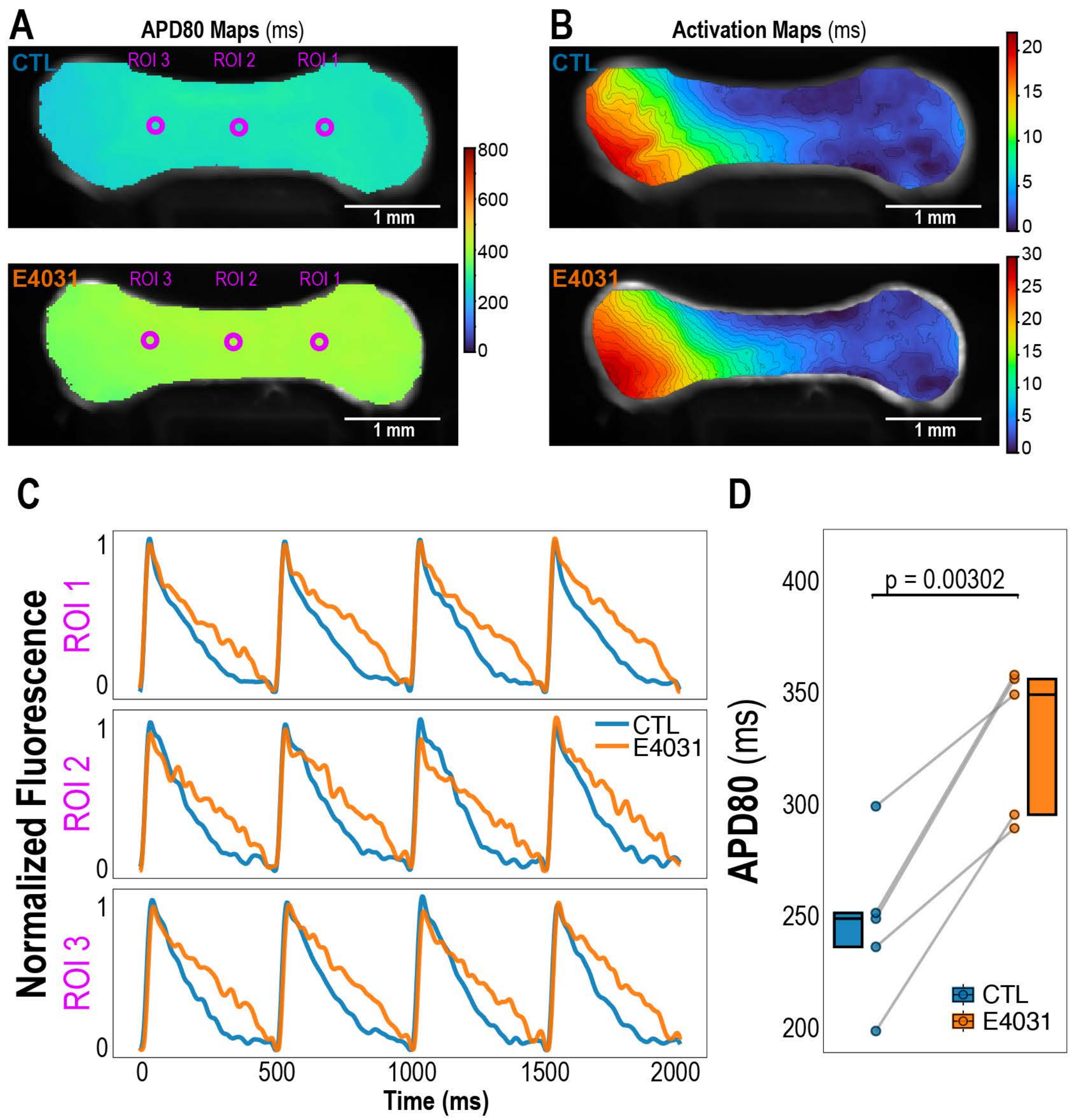

Supplemental Figure 7

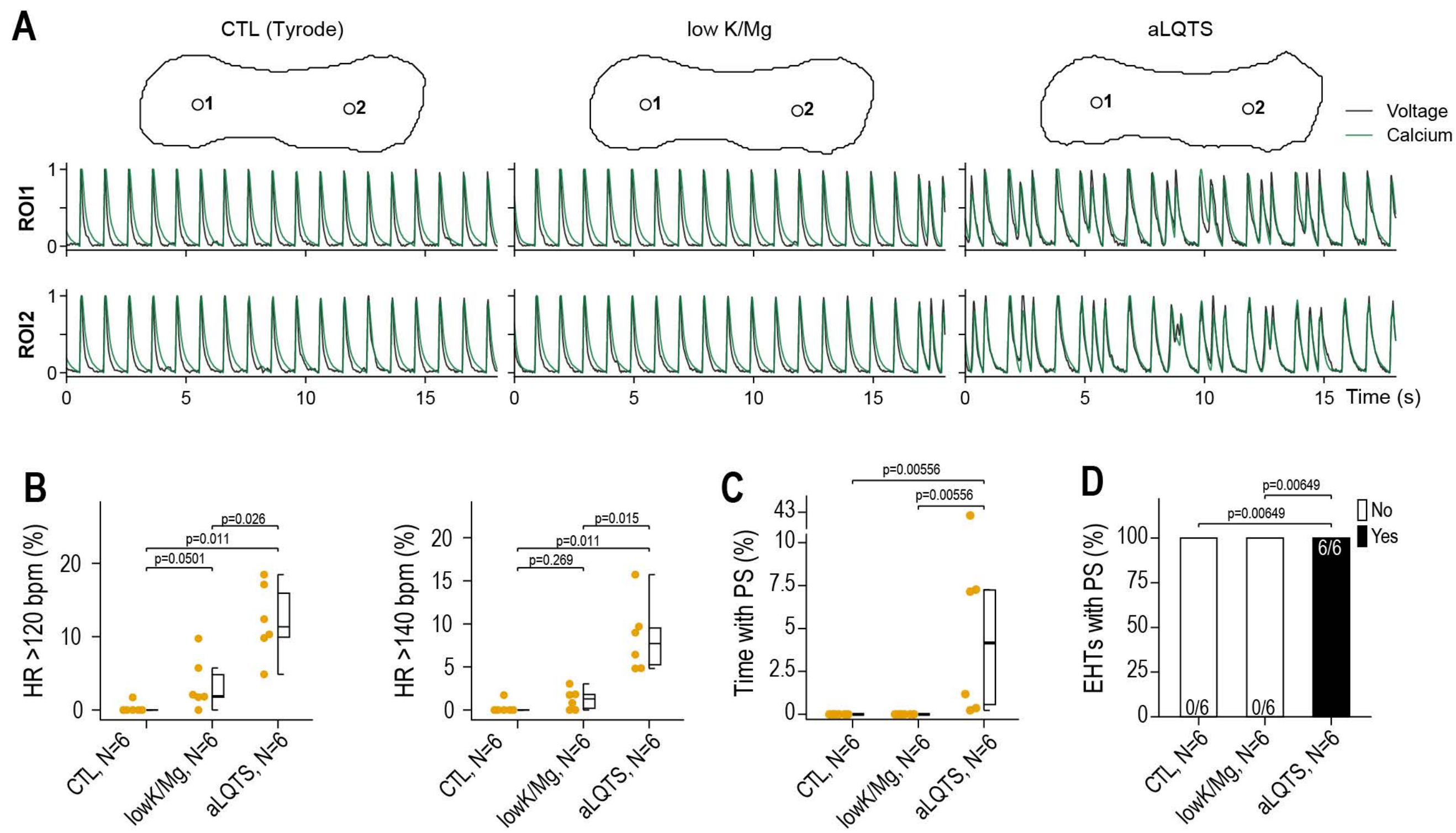

Supplemental Figure 8

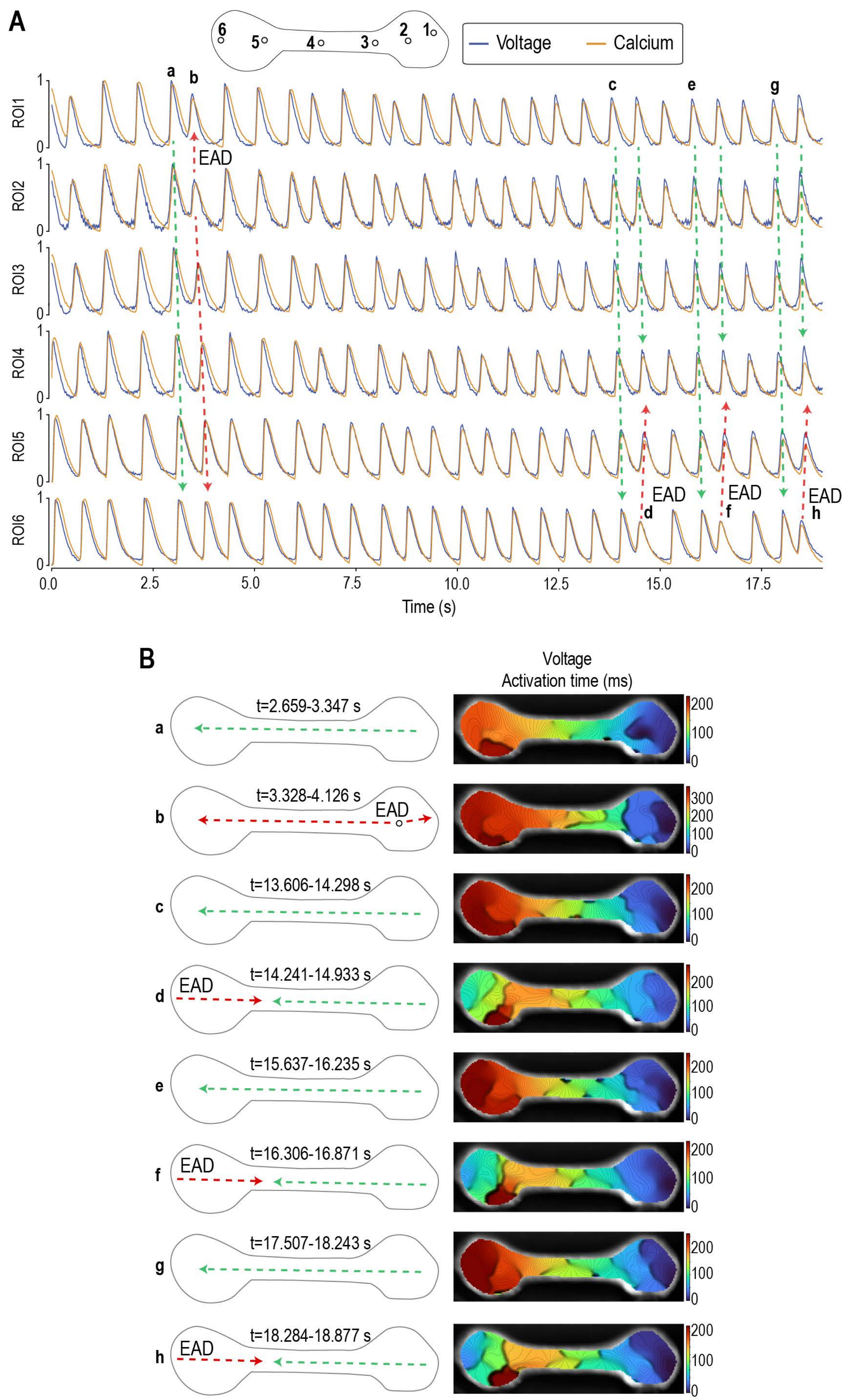

Supplementary Table 1. Summary of changes to milliPillar. The original CNC files from milliPillar publication[2] were slightly modified using Fusion 360 (Autodesk). The original and edited model renderings shown below were created in Fusion 360.

	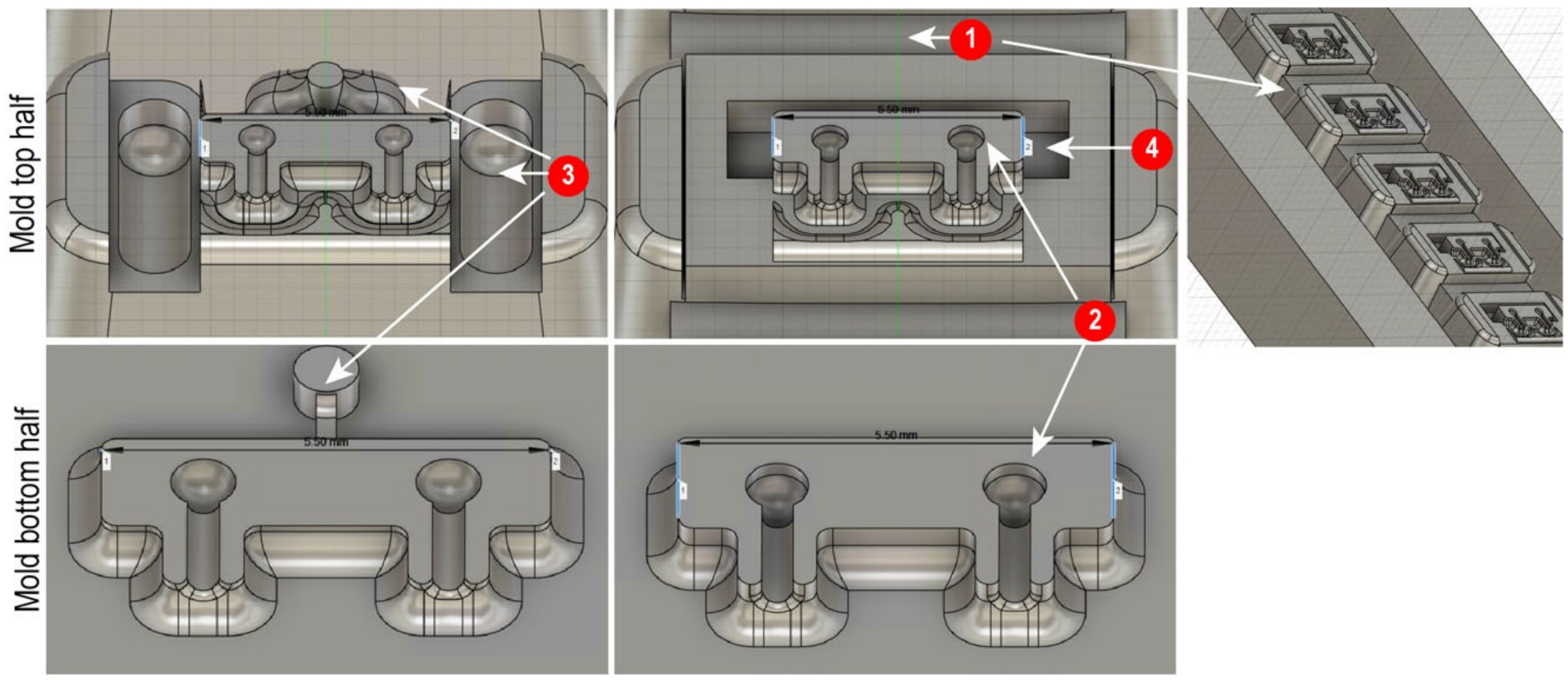 Rationale for changes:
1	Because of angular orientation in 3D printing, binding and detaching from FEP film on each layer caused deformation. Connecting all milliPillar modules reduced warping.
2	The top face around pillars was raised by 200μm. This allowed flat wet sanding of the print, eliminating 3D-printed lines that acted as micro-channels for PDMS bleed-through and generated a thin skin around the pillars. Registration pins were replaced for M2x6mm screws and holes.
3	Microfluidic port on the top of the milliPillar module and holes for carbon electrode bars were eliminated. This reduced the profile, allowing electrical stimulation with C-Pace (IonOptix) (Fig. 1F). Removal of microfluidics port was necessary because plasma treatment increased hydrophilicity, causing the EHT mixture to leak into this compartment and form a tissue-tissue strip that connected to the main EHT, altering its geometry and contraction.
4	A fence around the milliPillar pocket was added, allowing the EHT mixture to remain near the pillar heads by capillarity. The fence was used to grasp individual modules for gluing and handling.

Supplementary Table 2. Mold preparation

<table>
<tr><th></th><th>Step</th><th>Details</th></tr>
<tr><td>1</td><td>3D print mold</td><td>We used temperature-resistant resin. Phrozen Sonic Mini 8K using Phrozen TR300 resin with LycheeSlicer 7.4.500. The model was printed at 104% to account for resin contraction. Layer height was 30 μm. Print settings for burn and normal layers and anti-aliasing were as follows:

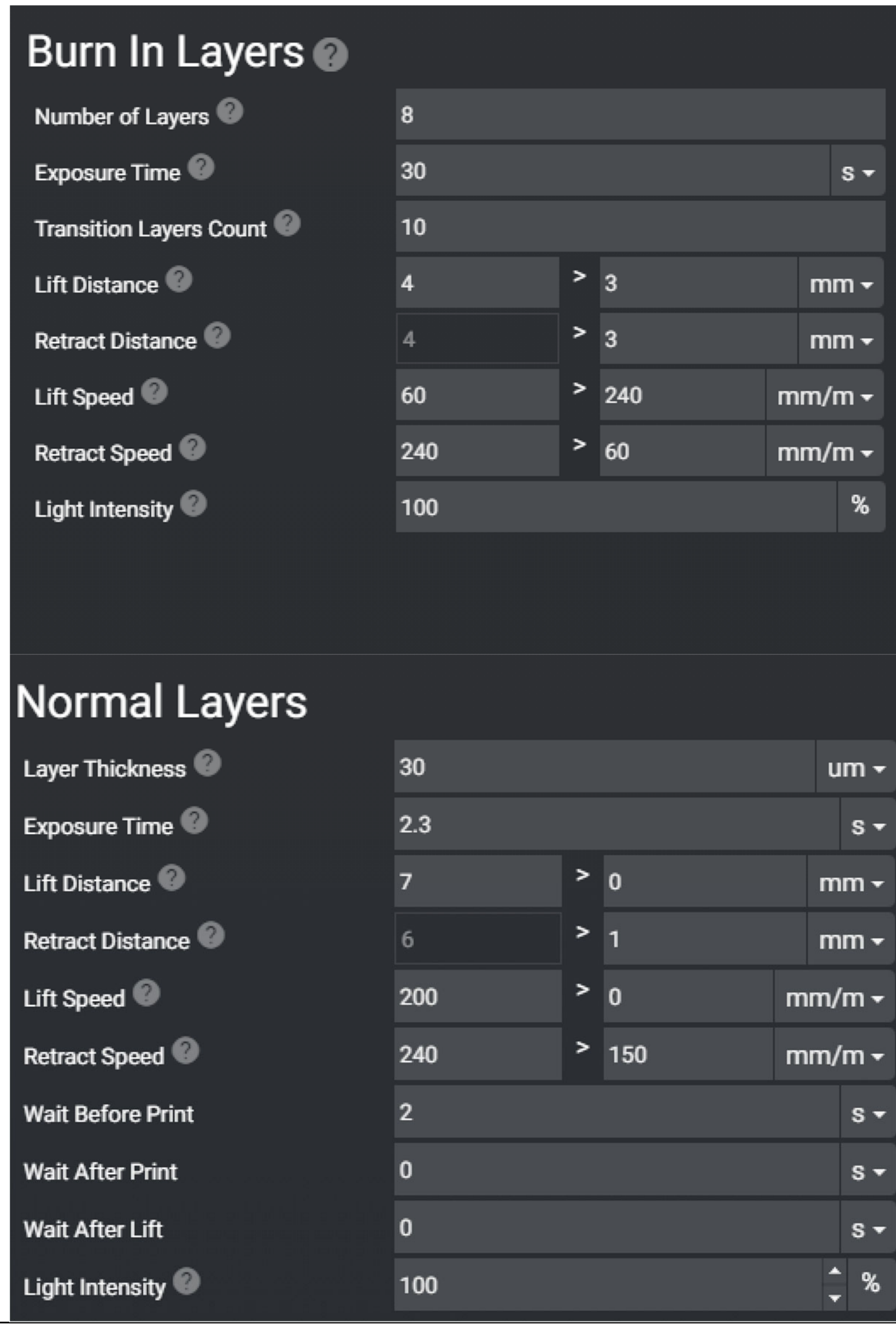

</td></tr>
</table>

<table>
<tr><td></td><td></td><td>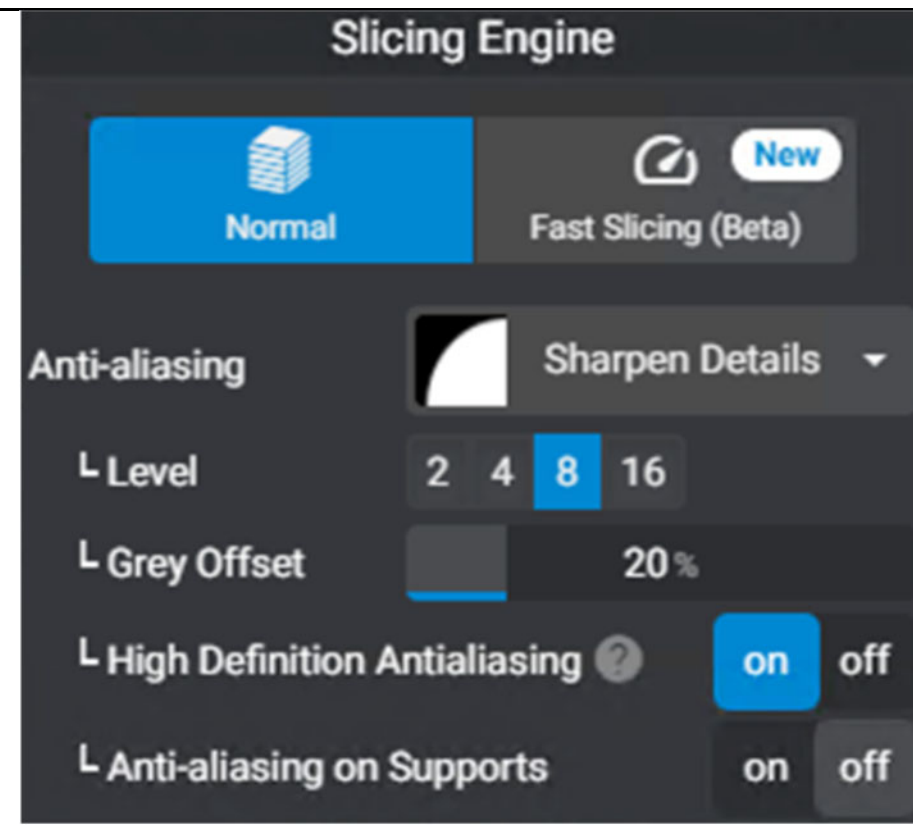

Prints were cleaned with isopropyl alcohol (IPA 91%) with a wash and cure station (e.g., ELEGOO Mercury Plus 2.0 Wash and Cure Machine)</td></tr>
<tr><td>2</td><td>Remove supports</td><td>The support and raft were removed by snapping it from the base side; due to the ~45° print tilt, the supports are shorter and more rigid at the base, allowing them to detach cleanly as one solid piece (Suppl. Fig. S1).</td></tr>
<tr><td>3</td><td>Clean thoroughly</td><td>Molds were submerged in IPA 91% and gently brushed to remove uncured resin from milliPillar modules. We incubated the mold for a maximum of 2 min in IPA (total) as an extended period can affect mold resistance.
The molds were air dried and cured using UV 405-nm with continuous rotation for 2 min (e.g., ELEGOO Mercury Plus 2.0 Wash and Cure Machine).</td></tr>
<tr><td>4</td><td>Flat sanding</td><td>Molds have a 200 μm added height on the pillar face. Opposing mold faces look as follows:
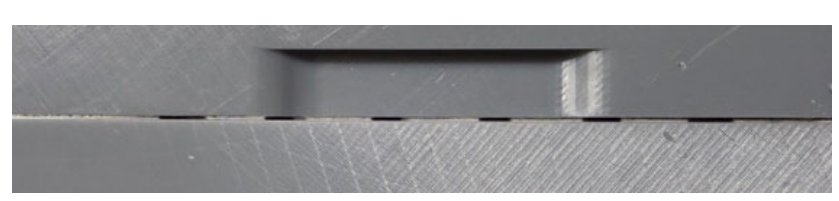
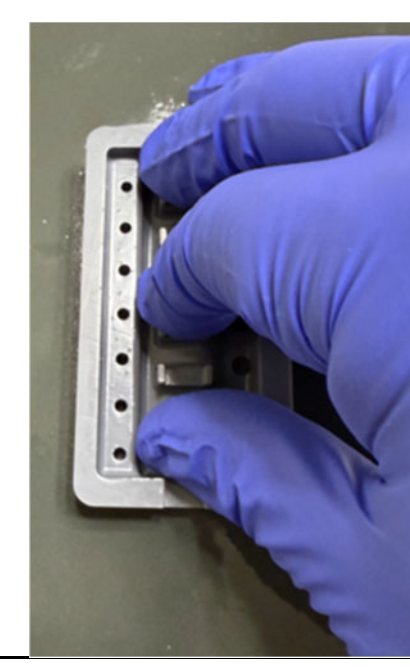
Wet 2000-grit sandpaper was used to remove excess material until the faces aligned perfectly (Fig. 1A).</td></tr>
<tr><td>5</td><td>Registration pins</td><td>Insert M2X6 mm screws were inserted to align the base plate, bottom, and top mold by applying gentle pressure.</td></tr>
<tr><td>6</td><td>Clean thoroughly</td><td>Sanding dust was removed from milliPillar modules by brushing and cleaning with IPA.</td></tr>
<tr><td>7</td><td>UV Cure</td><td>Molds were cured using UV 405 nm with continuous rotation for 1 h (e.g., ELEGOO Mercury Plus 2.0 Wash and Cure Machine); rotating the pieces after 30 min to allow homogeneous exposure.</td></tr>
</table>

8	Heat Cure	Heat cured for 3h at 100 °C using an oven inside the fume hood before reducing the temperature to 65 °C and left overnight. This treatment reduced cure inhibition.
9	Two rounds of PDMS casting	Ease Release 200 (Mann Release Technologies) was sparsely sprayed on each mold face. PDMS was degassed and cast as described in milliPillar paper.[2] Before closing and clamping the mold to cure overnight at 65 °C inside an oven in the fume hood.<br><br>After two rounds of casting, cure inhibition due to 3D printer resin was no longer observed.

Supplementary Table 3. PDMS for EHT preparation

	Step	Details
1	Clean mold thoroughly and apply release agent	Ease Release 200 (Mann Release Technologies) was sparsely sprayed on each mold face to help unmold PDMS.
2	Cast PDMS	PDMS was prepared with base-to-curing-agent 10:1 (Sylgard™ 184, #24236-10, Electron Microscopy Sciences), degassed, poured into the molds and degassed again.
3	Clamp and cure	Follow the milliPillar publication for details.[2] Briefly, molds were clamped using C-clamps (#1705A11, McMaster) and a 1/4" × 4" hex standoff (#91780A060, McMaster). Molds were topped off with PDMS and incubated at 65 °C overnight.
4	Unmold	Because of modifications, the PDMS platform is more prone to break compared to original milliPillar. Molds were gently removed by separating the sides of the platform with plastic pipet tips. The PDMS platform was removed and then further cured at 65 °C for additional 24 h.
5	Cleaning	After curing, molds were washed with MilliQ water, 70% ethanol and MilliQ water, then dried at 65 °C.
6	Cut and glue	Using a scalpel, modified milliPillar modules were cut and kept aligned on a clean and dust-free surface (**Fig. 1A, Suppl. Fig. 2**). Using a sterile L-shape cell spreader, a few drops of PDMS were spread on a clean sterile 6-well plate lid to make a thin film that was barely wet (**Suppl. Fig. 2A**). Then, forceps were used to grab the modified milliPillar fence (**Suppl. Fig. 2B**) and wet the bottom of each module with PDMS (without affecting pillars) and then transferred each module into a 6-well plate.<br><br>Spreading the thin layer of PDMS was the most effective and simple way to avoid excess PDMS wetting and filling between plate and milliPillar module. **Suppl. Fig. 2C** and **2D** show examples of incomplete filling and overfilling. Excess PDMS interfered with EHT plating, as these thin films detach when plating and get incorporated into the tissue or add extra material that deformed pillar heads (**Suppl. Fig. 2C**). Incomplete filling introduces an additional issue, as plasma treatment increases surface hydrophilicity, making the mixture prone to leaking underneath the milliPillar module.<br><br>To prevent plasticware deformation, the thin layer was cured overnight at 45 °C.
7	Corona plasma treatment	Corona plasma was delivered inside and around the milliPillar module using a spring-tip wire, and a Laboratory Corona Treater (#12201 and #BD-20AC, Electro-Technic Products).

8	Wash and decontamination	Wells were washed twice with 70% ethanol. Ethanol was incubated for 30 minutes inside the biosafety cabinet and then aspirated and air-dried.<br><br>Plates and lids were treated for 20 min with UV-C in a UV Clave (B1450, Benchmark Scientific; 600mJ/cm^2, 20 min cycle) inside the biosafety cabinet. UV-C indicator stickers (B1450-ISS, Benchmark Scientific) monitored UV-C efficiency and penetrance. UV-C went under milliPillar PDMS but did not cross the 6-well plastic lid. Therefore, both plate and lid were treated separately, with inner faces exposed directly to UV-C.
9	Blocking	KOSR blocking prevented collagen hydrogel sticking to milliPillar module, removing the need to needle-score each individual EHT at 1 and 24h post cure as originally described.[2] This made plating faster and EHT formation more reproducible.<br><br>A volume of 25 μL of KOSR was enough to coat the entire milliPillar pocket. After 1 h incubation at 37 °C, we aspirated and dried KOSR. It is important that no leakage is observed at this step between the interface of PDMS and plastic. Drying well KOSR before plating prevents cell-hydrogel mixture leak underneath by capillarity. However, it is recommended to properly glue milliPillar inserts as described above and in **Suppl. Fig. 2**.
10	Plating	Confluent hiPSC-CMs and cardiac fibroblasts were detached using TryPLE Select 10x supplemented with 5 μM Y-27632 for approximately 15 min. Equal volume of plating media (RPMI, 1x B27, 10% KOSR, 1X Pen Strep, 5 μM Y-27632) was added and pipetted up and down, spun and resuspended in 5-10 mL of fresh plating media.<br><br>A total of 550,000 cells (75% cardiomyocytes/25% fibroblasts) were used for each EHT. Batches of at least 12 EHTs were plated simultaneously. For each EHT, cells were pelleted and resuspended in 15 µL of matrix solution per EHT, consisting of 12 µL of PureCol® EZ Gel (BICO 5074, 5 mg/mL bovine atelocollagen) and 3 µL plain RPMI supplemented with 25 µM Y-27632, to achieve a final collagen concentration of 4 mg/mL and 5 µM Y-27632.<br><br>EHT solution (15 µL) was homogeneously dispersed without introducing bubbles and applied to each milliPillar module pocket near the pillar heads by sweeping the pipette tip across the pocket and dispensing on the opposite pillar. We also spread the mixture around the upper fence to ensure the

		mixture remained around pillar heads by capillarity. A 25 µL of plating media was added around the plastic well borders to add extra moisture and prevent dehydration.<br><br>Cell-collagen hydrogel mixture was incubated for 1 h at 37 °C to allow collagen polymerization and then 4 mL of fresh plating media was added against the wall to avoid disturbing the mixture.
11	Maturation	After 24 h of plating, media was replaced with maturation media based on DMEM low glucose containing 1X B27, 1X penicillin/streptomycin, 10% KOSR, 5 µg/mL cobalamin, 0.82 µM biotin, and 1X non-essential amino acids, 1X normocin. Media was refreshed every 2-3 days. From day 7 to 21, 100 nM triiodothyronine and 1 µM dexamethasone were added.[3]

Supplementary Table 4: Primers used in this study were described in Deogharia et al.[4]

Gene	Forward primer	Reverse primer	Efficiency (%)
18S	ATCGGGGATTGCAATTATTC	CTCACTAAACCATCCAATCG	101.3
GAPDH	CTTTTGCGTCGCCAG	TTGATGGCAACAATATCCAC	96.5
TBP	GCCAAGAGTGAAGAACAG	GAAGTCCAAGAACTTAGCTG	91.7
SCN5A	GAAGAGTTAGAGGAGTCTCG	TGATGGTGAGGTCAGTAAAC	99
KCNJ2	AGCCTATGGTTGTCTGGGTCT	TGGATGCTGGTTATCTTCTGC	97.5
KCNJ12	CCAGTGCAACATTGAGTTCGCC	CAGCGAGAAGATGAGCAGCATG	99.2
GJA1	ATCCTCCAAGGAGTTCAATC	AATGAAAAGTACTGACAGCC	98.8
ATP2A2	ATGGGGCTCCAACGAGTTAC	TTTCCTGCCATACACCCACAA	90.7
PLN	CAAGGCTACCTAAAAGAAGAC	TGGACTTTCTCCATGATACC	97.2
RYR2	ACAACAGAAGCTATGCTTGGC	GAGGAGTGTTCGATGACCACC	94.9
JPH2	AACACCATCCTCATCTGC	AACTTCTGGCTCTGGTAAG	103.6
CAV3	GACCCCAAGAACATTAACGAGG	GGACAACAGACGGTAGCACC	100.4
ACTC1	TAAGATTATTGCTCCCCCTG	GGAGAGAGAAGGCATCTTAG	92.7
MYH6	AGCAGAGAAACTTTGACAAG	TCCTGAAGGTTCTTGTTCTC	91.5
MYH7	GGATGAGGAGGAGATGAATG	TTTCACCTTGTTCTCTGTTG	95.7
MYL2	GTCCCTACCTTGTCTGTTAG	TTTGTCAGAGAAATGTCGTG	106.8
MYL7	GGAGTTCAAAGAAGCCTTCAGC	AAAGAGCGTGAGGAAGACGG	93.3
MYBPC3	CCATGACGCTGAGGTCAAATG	GCTTGGCACCGATGGACTC	93.6
TNNI1	AGAAGGAAGACACAGAGAAG	GGCATCAAACATCTTCTTCC	97.2
TNNI3	GCAAGAAAAAGTTTGAGAGC	TTTTTCAGCTCAGAGAGAAG	93.5
TNNT2	GGAGGAGTCCAAACCAAAGCC	TCAAAGTCCACTCTCTCTCCATC	92.8
TCAP	GTCGGAGGAGAACTGTGAGC	ATGCCCATCCGCATCATCAG	94.3
TTN	CCCCATCGCCCATAAGACAC	CCACGTAGCCCTCTTGCTTC	94.5
VCL	CTCGTCCGGGTTGGAAAAGAG	AGTAAGGGTCTGACTGAAGCAT	103.4
NDUFB8	ACAGGAACCGTGTGGATACAT	CCCCACCCAGCACATGAAT	98.4
SDHB	GACACCAACCTCAATAAGGTCTC	GGCTCAATGGATTTGTACTGTGC	98.7
UQCRH	GAGGACGAGCAAAAGATGCTT	CGAGAGGATACACGCTCATCA	91.5
COX5A	ATCCAGTCAGTTCGCTGCTAT	CCAGGCATCTATATCTGGCTTG	96.1
ATP5F1A	GTATTGCCCGCGTACATGG	AGGACATACCCTTTAAGCCTGA	98.8
SLC27A1	GGGGCAGTGTCTCATCTATGG	CCGATGTACTGAACCACCGT	100.8
CPT1B	AGAATTCCAGGACAAGACTG	CACTCACATAGTTACTTGCC	92.2
ACADVL	ACAGATCAGGTGTTCCCATACC	CTTGGCGGGATCGTTCACTT	98.6
ACADL	TGCAATAGCAATGACAGAGCC	CGCAACTACAATCACAACATCAC	99.1
ACADM	TGGATAACCAACGGAGGAAAAG	CTGGGGTATCTGCTTCCACA	93.5
ACADS	CGGCAGTTACACACCATCTAC	GCAATGGGAAACAACTCCTTCTC	90.2
HADHA	ACCGAGGACAGCAACAAGTG	TCAAGCTGCCCAGTCAAGTT	92.9
HADHB	CTGAACCTTGCTCCGAGAGG	GCCCATTTTGATGCAGTGGG	95.6

SUPPLEMENTARY VIDEO LEGENDS

Supplementary Video 1. Handling of milliPillar modules. Video shows how to detach milliPillar modules from multi-well plates using forceps.

Supplementary Video 2. Representative contractility trace. *Left panel*, contractility recording video. *Middle panel*, BeatProfiler-generated video showing pillar tracking over time. *Right panel*, shows quantification of force overtime. Videos were recorded at 1, 2 and 3 Hz, for 60 s each. Orange segmented line represents the baseline at 1 Hz

Supplementary Video 3. Dual voltage and Ca^{2+} optical mapping with point stimulation at 1 Hz (CL = 1000 ms). *Top panel*, Voltage and Ca^{2+} maps showing wave propagation across the EHT. A jet-style color scale (blue to red) depicts normalized signal intensity over time. *Bottom panel*, simultaneous normalized signal quantification at ROI1, 2 and 3. Point electrical stimulation was applied on the right side of the EHT at 1 Hz.

Supplementary Video 4. Dual voltage and Ca^{2+} optical mapping with point stimulation at 3 Hz (CL = 333 ms). Same as described for **Suppl. Video 3**.

Supplementary Video 5. Representative CTL recording shows synchronized capture throughout the entire longitudinal axis due to field stimulation. *Left panel*, Normalized voltage and Ca^{2+} fluorescence (normalized 0-1 in turbo color scale), *Right panel,* phase calculated from these recordings (scale $-\pi$ to π, in turbo color scale). The video corresponds to the ~15-19 s interval from the Kymograph on **Fig. 4C** top panel is shown

Supplementary Video 6. Events occurring between ~15-19 s from the representative aLQTS recording shown in **Fig. 4C**, which shows activation originating from the top-left corner of the EHT that progressively slows as the beating frequency accelerates before wavebreak, with partial recovery towards the end of the recording. *Left panel*, Normalized voltage and Ca^{2+} fluorescence (normalized 0-1 in turbo color scale), *Right panel,* phase calculated from these recordings (scale $-\pi$ to π, in turbo color scale).

Supplementary Video 7. Top panel, normalized voltage and Ca^{2+} fluorescence (normalized 0-1 in turbo color scale). Bottom panel, animated version of **Fig. 5** showing EAD propagation in a representative aLQTS EHT.

Supplementary Video 8. Events occurring between ~10-14 s from the representative aLQTS recording shown in **Fig. 6A**. Video shows from beats 12-19 and the formation of rotors on both EHT heads as described in **Fig. 6I**. *Left panel*, Normalized voltage and Ca^{2+} fluorescence (normalized 0-1 in turbo color scale), *Right panel,* phase calculated from these recordings (scale $-\pi$ to π, in turbo color scale).

Supplementary Video 9: Wavelet and PS localization from **Fig. 7** showing events occurring between ~0-6 s. *Top panel*, voltage signal (normalized 0-1 in turbo color scale), *middle panel*, phase calculations (scale $-\pi$ to π, in turbo color scale) with wavelet outline overlap in white (kept) or orange (excluded because they did not meet size or time criteria as described in Methods), *bottom panel*, binary wavelet mask. PS are shown as circles when they met 1.8π criteria as described in the Methods section. **Supplementary Video 9**a was obtained in iPSC 1 and **Supplementary Video 9**b in iPSC 2.

Supplementary Video 10–12. PS tracks after cryoinjury from Figure 8G a–c. Side-by-side dual-camera renderings of matched control (*left panel*, "Before Cryoinjury") and cryoinjured (*right panel*, "After Cryoinjury") from 3 independent EHTs with the longest rotor tracks. Each half shows,

top-to-bottom: voltage signal (V) and calcium signal (Ca), rescaled to a 0–1, excluding the cryoinjured area; the corresponding Hilbert phase maps for Voltage and Calcium; a bottom row with PS trajectories on the voltage channel and a table of absolute rotor times for the recording. Phase-singularity (PS) locations are overlaid as black-and-white halo circles on both voltage panels (signal and phase); each halo is annotated with its per-recording track ID, matching the numbering in the table legend, a. recording 1, 0 rotor tracks in the matched control, 5 rotor tracks after cryoinjury. b. recording 2, 0 rotor tracks in the matched control, 2 rotor tracks after cryoinjury. c. recording 3, 0 rotor tracks in the matched control, 3 rotor tracks after cryoinjury.

SUPPLEMENTARY STL FILES

STL file 1. Modified milliPillar mold including rafts and supports.
STL file 2. Perfusion stage (part 1/6). Perfusion heater holder for ThermoClamp pencil.
STL file 3. Perfusion stage (part 2/6). Base to hold perfusion heater holder and tissue perfusion mount. Use epoxy to glue perfusion heater holder to base.
STL file 4. Perfusion stage (part 3/6). Tissue perfusion mount.
STL file 5. Perfusion stage (part 4/6). Perfusion holder to mount blunt needles.
STL file 6. Perfusion stage (part 5/6). Clamp to hold EHTs.
STL file 7. Perfusion stage (part 6/6). Electrical stimulation slider.

REFERENCE FOR SUPPLEMENTARY INFORMATION

1. Cooper, B.L., Gloschat, C., Swift, L.M., Prudencio, T., McCullough, D., Jaimes, R., and Posnack, N.G. (2021). KairoSight: Open-Source Software for the Analysis of Cardiac Optical Data Collected From Multiple Species. Front Physiol *12*, 752940. 10.3389/fphys.2021.752940.

2. Tamargo, M.A., Nash, T.R., Fleischer, S., Kim, Y., Vila, O.F., Yeager, K., Summers, M., Zhao, Y., Lock, R., Chavez, M., et al. (2021). milliPillar: A Platform for the Generation and Real-Time Assessment of Human Engineered Cardiac Tissues. ACS Biomater Sci Eng *7*, 5215–5229. 10.1021/acsbiomaterials.1c01006.

3. Wang, L., Wada, Y., Ballan, N., Schmeckpeper, J., Huang, J., Rau, C.D., Wang, Y., Gepstein, L., and Knollmann, B.C. (2021). Triiodothyronine and dexamethasone alter potassium channel expression and promote electrophysiological maturation of human-induced pluripotent stem cell-derived cardiomyocytes. J Mol Cell Cardiol *161*, 130–138. 10.1016/j.yjmcc.2021.08.005.

4. Deogharia, M., Venegas-Zamora, L., Agrawal, A., Shi, M., Jain, A.K., McHugh, K.J., Altamirano, F., Marian, A.J., and Gurha, P. (2024). Histone demethylase KDM5 regulates cardiomyocyte maturation by promoting fatty acid oxidation, oxidative phosphorylation, and myofibrillar organization. Cardiovasc Res *120*, 630–643. 10.1093/cvr/cvae014.
